# Gas-Phase Peroxyl Radical Recombination Reactions: A Computational Study of Formation and Decomposition of Tetroxides

**DOI:** 10.1021/acs.jpca.2c01321

**Published:** 2022-06-16

**Authors:** Vili-Taneli Salo, Rashid Valiev, Susi Lehtola, Theo Kurtén

**Affiliations:** † Department of Chemistry, Faculty of Science, University of Helsinki, Helsinki FI-00014, Finland; ‡ Molecular Sciences Software Institute, Blacksburg, Virginia 24061, United States

## Abstract

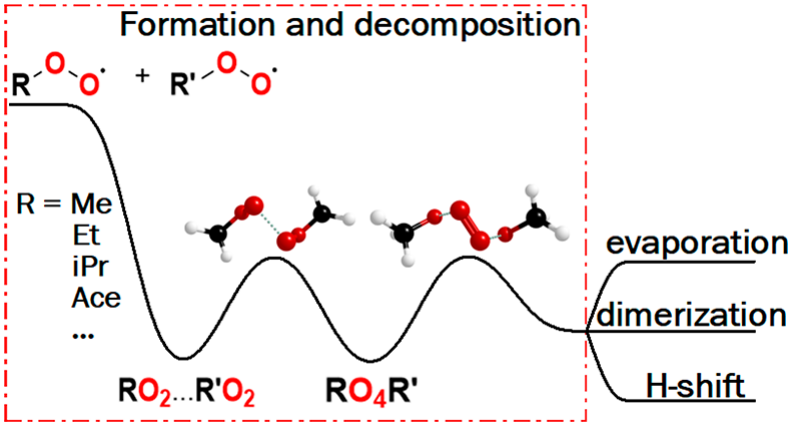

The recombination (“dimerization”) of peroxyl radicals (RO_2_•) is one of the pathways suggested in the literature for the formation of peroxides (ROOR′, often referred to as dimers or accretion products in the literature) in the atmosphere. It is generally accepted that these dimers play a major role in the first steps of the formation of submicron aerosol particles. However, the precise reaction pathways and energetics of RO_2_• + R′O_2_• reactions are still unknown. In this work, we have studied the formation of tetroxide intermediates (RO_4_R′): their formation from two peroxyl radicals and their decomposition to triplet molecular oxygen (^3^O_2_) and a triplet pair of alkoxyl radicals (RO•). We demonstrate this mechanism for several atmospherically relevant primary and secondary peroxyl radicals. The potential energy surface corresponds to an overall singlet state. The subsequent reaction channels of the alkoxyl radicals include, but are not limited to, their dimerization into ROOR′. Our work considers the multiconfigurational character of the tetroxides and the intermediate phases of the reaction, leading to reliable mechanistic insights for the formation and decomposition of the tetroxides. Despite substantial uncertainties in the computed energetics, our results demonstrate that the barrier heights along the reaction path are invariably small for these systems. This suggests that the reaction mechanism, previously validated at a multireference level only for methyl peroxyl radicals, is a plausible pathway for the formation of aerosol-relevant larger peroxides in the atmosphere.

## 1 Introduction

Peroxyl radicals are primary intermediates in the oxidation of volatile organic compounds (VOC), such as hydrocarbons, in the atmosphere.^1,2^ These compounds and their downstream oxidation and aggregation products play an essential role in the formation of tropospheric secondary organic aerosols (SOA).^3,4^ Not only do tropospheric aerosols and other fine particulate matter pose severe respiratory and cardiovascular health risks in polluted urban areas,^5,6^ but they also modulate climate on a global scale.^7^

Hydrocarbons are emitted into the atmosphere via both anthropogenic and biogenic processes.^8,9^ The main emitted hydrocarbons include methane, ethane, other volatile higher alkanes, alkenes, alkynes, ketones, aldehydes, etc. Simple aliphatics, aromatics, and alkenes are mainly of anthropogenic origin; sources include incomplete fossil fuel burning, leaking or evaporation of volatile organic compounds in industrial processes, and human-controlled biological processes.^9^ Biogenic hydrocarbon emissions can be divided into two subcategories: emissions due to microbial activity and emissions of biosynthetic VOCs by higher plants. The bulk of the biogenic emissions are composed of methane from microbial methanogenesis,^10^ and of isoprene and other volatile terpenes and terpenoids from plants.^11^ The biogenic C_10_ compound family known as monoterpenes has especially received much attention in SOA studies, as some of the C_10_ compounds’ oxidation products likely have sufficiently low volatilities to participate in new-particle formation.

Once peroxyl radicals have been formed, a plethora of further reactions may occur. An important pathway with respect to aerosol formation is autoxidation, in which the peroxyl radical moiety undergoes an intramolecular hydrogen-shift to form a hydroperoxide alkyl radical, which may again react with molecular oxygen to form a more oxygenated and complex peroxyl radical compound.^12^ This kind of reaction pathway can rapidly increase the oxygen content and the mass of the molecule, giving rise to so-called highly oxygenated molecules (HOM).^13^ The radical propagation mechanism can terminate, for example, when the radical hydrogen abstraction occurs at a carbon containing a hydroperoxide group, as the •C–OOH group rapidly rearranges into a closed-shell carbonyl compound and a free hydroxyl radical.^14^

In addition to intramolecular reactions, peroxyl radicals also react via intermolecular pathways. In high NO_*x*_ concentrations, peroxyl radicals can react with NO• to produce alkoxyl radicals and NO_2_•, or with NO_2_• to produce peroxynitrates. In low NO_*x*_, peroxyl radicals react mainly with HO_2_• to form alkyl hydroperoxides.^15^

**Scheme 1:**
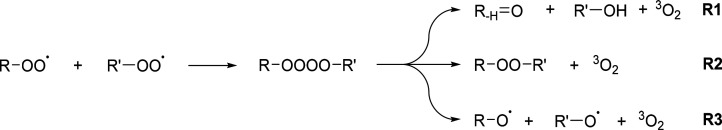
Intermolecular Self- and Cross-Reactions of Peroxyl Radicals

Although they are not the main channel for peroxyl radical reactions, self- and cross-reactions with other peroxyl radicals may play a central role in SOA formation.^16,17^ These reactions can be categorized into propagating reactions and terminating reactions. In the former, the radical character of the molecule(s) is retained and propagates to the next stage, while in the latter only closed-shell molecules are eventually produced, and the reaction chain thereby terminates. These two types of mechanisms are visualized in Scheme 1.

The current consensus is that the majority of peroxyl radical cross-reactions occur through tetroxide intermediates as seen in Scheme 1.^18−22^ Few studies have elucidated the mechanisms involved in the formation of the tetroxide intermediate (RO_4_R′) and in the subsequent decomposition into various product channels. The original study of tetroxide intermediates by Russell suggested a cyclic, concerted decomposition transition state into aldehyde/ketone, alcohol, and molecular oxygen (Scheme 1, R1).^18^ The end products of this mechanism have been experimentally verified to be the main products of primary and secondary peroxyl radical self- and cross-reactions in the liquid phase (R1 is not possible for tertiary alkoxyl radicals due to absence of α-oxyl hydrogens). In the gas phase, both the alcohol + carbonyl (Scheme 1, R1) and alkoxyl radicals (Scheme 1, R3) have substantial yields.^23−25^ However, the Russell mechanism itself has been deemed unlikely, as it is inconsistent with thermodynamic experiments and computational studies.^26−29^

**Scheme 2:**
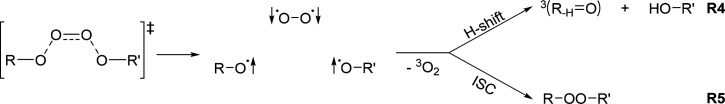
Sequential Decomposition of a Tetroxide Intermediate into Triplet Molecular Oxygen and Alkoxyl Radicals, and the Two Product Channels: H-Shift and ISC

Seminal theoretical work by Ghigo et al.^30^ investigated the decomposition reaction of dimethyltetroxide (MeO_4_Me) with the CASSCF (Complete Active Space Self-Consistent Field) method.^31^ The initial effort in that study was to elucidate the cyclic decomposition of the tetroxide into alcohol and carbonyl products (Scheme 1, R1), but transition states corresponding to such a mechanism were not found. The possibility to decompose into singlet molecular oxygen and two alkoxyl radicals coupled into a singlet was deemed unlikely on thermodynamical grounds and not investigated further. Such a mechanism could still explain the experimental observation of singlet molecular oxygen. This led to the suggestion that the tetroxide may decompose first into triplet molecular oxygen and two doublet alkoxyl radicals, adding up to a total singlet multiplicity (Scheme 1, R3, and Scheme 2). Then, after the initial decomposition, both the Russell-product channel (Scheme 2, R4) and dimerization channel (Scheme 2, R5) are available via intermolecular H-shift and intersystem crossing (ISC), respectively. Alkoxyl radical formation (Scheme 1, R3) simply corresponds to dissociation of the product complex.

The original study by Ghigo et al.^30^ suggested that ISC is important but did not provide any theoretical results on the matter. This motivated our previous work on thorough investigation of the intersystem crossings for the MeO_4_Me model system.^32^ In later work, we used this approach for H-shifts and dimerization reactions for ^3^(RO···R′O) complexes corresponding to several atmospherically relevant and model RO_2_• + R′O_2_• systems.^33^

In this work, we aim to complement our previous discoveries by completing the reaction path from the beginning. We elucidate the reaction pathway from the separate peroxyl radicals to the tetroxide intermediate and finally to the decomposition of the tetroxide via the mechanism illustrated in the left-hand-side reaction of Scheme 2; the reaction pathway downstream of this decomposition is already covered by our previous work.^33^

## 2 Theoretical Methods

### 2.1 Conformational Sampling of Tetroxides, Peroxyl Radicals, and Alkoxyl Radicals

The investigated tetroxides, and most peroxyl radicals and alkoxyl radicals, have many conformational isomers. Systematic sampling of the conformational space was therefore carried out with the Spartan 16 program.^34^ In the sampling, the torsional degrees of freedom (excluding the rotation of methyl groups) were scanned as a function of the potential energy calculated with the Merck Molecular Force Field (MMFF94) level of theory.^35^ To avoid erroneous description of peroxyl radicals and alkoxyl radicals as anions instead of neutral radicals, the keyword ffhint=”Ox∼∼6” (x is the number of the terminal oxygen) was used to force the terminal oxygen to be a neutral radical. Unique local potential energy minimum conformers resulting from the sampling were then collected. More accurate single-point energies for the conformers were obtained at the B3LYP/6-31+G(d)^36−42^ level of theory with Gaussian 16.^43^ At this stage, a 5 kcal/mol threshold compared to the lowest-energy conformer was used as a cutoff for pruning the list of conformers. Molecular geometries of the remaining conformers were optimized at the B3LYP/6-311+G(d)^37,44^ level of theory. Duplicates based on close or identical total energies and dipole moment values were removed from the conformer list, and a cutoff of 2 kcal/mol was used to further limit the number of conformers. Finally, the remaining conformers were reoptimized at the ωB97X-D/aug-cc-pVTZ^45−47^ level of theory with Gaussian 16. The obtained global minimum conformers were used in further calculations.

The choice of the functionals and the corresponding basis sets in the conformational sampling follow the trend of increasing accuracy (and cost) as the number of conformers decreases throughout the sampling. This sampling methodology is based on the cost-effective scheme for unimolecular peroxyl radical reactions developed by Møller et al.^48^ and has been successfully employed in our past research on bimolecular peroxyl radical reactions.^32,33^

### 2.2 Geometry Optimization of the Stationary Points along the Reaction Coordinate with CASSCF/6-311++G(d,p)

The active spaces in the CASSCF optimizations are constructed similarly to our previous studies, where we used the (10,8) active space for investigating the methyl peroxyl self-reaction.^32^ A detailed description of how the active space was set up can be found in the Supporting Information (SI, Section S2). All CASSCF calculations were done with the Firefly QC package,^49^ which is partially based on the GAMESS-US source code.^50^ We used the state-specific formalism of the theory on a singlet potential energy surface.

In the geometry optimization, very tight numerical thresholds were used in all parts of the calculations. This choice was made because in Firefly, only numerical Hessian evaluations are available for MCSCF. The maximum allowed asymmetry in the Lagrangian matrix was set to 1.0 × 10^–8^ a.u. instead of the default 1.0 × 10^–5^ a.u. The SCF energy convergence criteria were changed from the default 1.0 × 10^–10^ a.u. to 1.0 × 10^–12^ a.u. Convergence criteria for the molecular geometry, defined in terms of the largest component of the gradient, were also tightened from the default value 1.0 × 10^–4^ a.u. to 7.0 × 10^–6^ a.u. All convergence criteria were relaxed to the default values for the 15 Å separated pair of RO_2_• radicals and for the 2RO• + ^3^O_2_ product complex. The presence of a frozen cordinate in the former system means that optimized structures would in any case not correspond to minima on the potential energy surface, so vibrational analyses were skipped altogether in this case. Convergence criteria were relaxed for the latter system due to difficulties in reaching geometry convergence (presumably at least partially due to a flat potential energy surface caused by the very limited interaction of O_2_ with the alkoxyl radicals).

To obtain consistent and comparable vibrational frequencies for the non-interacting RO_2_• + RO_2_• peroxyl radical pair, the (10,8) active space was split in half into (5,4) active spaces for each RO_2_•. The active space orbitals were chosen carefully to match the active space orbitals localized onto each of the RO_2_• fragments in the (10,8) RO_2_• + RO_2_• pair. Furthermore, to confirm that the 2 × (5,4) active space was size-consistent with the (10,8) active space, CASSCF(5,4)/6-311++G(d,p) single-point energies for all the RO_2_• monomer geometries in the RO_2_• + RO_2_• pairs were calculated and compared against CASSCF(10,8) energies. Afterward, geometry optimization and vibrational frequencies for the RO_2_• structures were calculated with CASSCF(5,4)/6-311++G(d,p) using the same tightened criteria as described previously. The results of the size-consistency analysis, which indicates that the CASSCF “split active space” results are size-consistent while the corresponding XMC-QDPT2 (Extended Multi-Configurational Quasi-Degenerate Perturbation Theory at Second Order of Perturbation) results are not, can be found from the SI).

For transition state calculations, fully numerical Hessians were calculated at the beginning of all first-order saddle-point optimizations. For both minima and transition states, the full numerical Hessian was calculated at the end of the geometry optimization to verify the nature of the obtained stationary points. Minima were identified by positive-definite Hessian matrices. In a few cases, due to the numerical nature of Hessian analysis, some imaginary frequencies persisted, but they were verified to correspond to either translational or rotational modes via Sayvetz condition analysis.^51^ The corresponding structures were exclusively either loosely bound reactant complexes or 15 Å separated peroxyl radicals. Transition state structures were also identified by their Hessian matrices, having only one negative eigenvalue (all others non-negative) with a related eigenvector corresponding to motion along the reaction coordinate. The corresponding imaginary vibrational frequencies can be found for all transition states in the SI section listing the optimized geometries from CASSCF(10,8) and CASSCF(5,4) calculations (SI, Section S10). The numerical Hessians were calculated with doubled displacements (0.005 a.u. displacement in both positive and negative direction, 7-point stencil) in all three Cartesian directions to reach better accuracy.

### 2.3 XMC-QDPT2(10,8) Single-Point Calculations

XMC-QDPT2(10,8)/6-311++G(d,p) optimizations for the CH_3_O_2_• + CH_3_O_2_• system were carried out in our previous study.^32^ Due to the computationally demanding nature of XMC-QDPT2(10,8)/6-311++G(d,p) calculations,^52^ further geometry optimizations were not conducted at this level of theory in this study. Instead, single-point total energy calculations were carried out on the CASSCF(10,8) optimized geometries of all stationary points to better take into account dynamic electron correlation effects within the investigated systems. SCF convergence criteria were relaxed in a similar manner to the CASSCF calculations, for the 15 Å separated RO_2_• pairs and 2 RO• + ^3^O_2_ structures. XMC-QDPT2 calculations were done with the Firefly QC package.^49^

### 2.4 ASCI-SCF/PT2 Single-Point Calculations

The adaptive sampling configuration interaction (ASCI) method^53^ enables accurate multiconfigurational calculations in large active spaces. We employed the ASCI-SCF (ASCI self-consistent field) method^54^ to further validate the CASSCF(10,8) method. Full-valence ASCI-SCF calculations were carried out for the HO_4_H, MeO_4_H, and MeO_4_Me systems at the corresponding CASSCF geometries in the 6-311++G(d,p), cc-pVDZ, and the cc-pVTZ basis sets.^47^ ASCI-SCF orbital optimization was carried out with ASCI wave functions containing 10^5^ determinants. These orbitals were then employed in further ASCI calculations. The ASCI and ASCI-SCF calculations were carried out with a development version of Q-Chem versions 5.4 and 5.4.1.^55^

The effect of the truncation of the ASCI wave function was studied in two ways: by increasing the number of variationally determined determinants from 10^5^ to 5 × 10^6^ and/or by examining perturbative corrections to the ASCI energy. The results appear to be converged beyond the required accuracy, especially when the perturbative correction is used. The ASCI results, which support our central findings, are discussed in the SI (Section S7).

### 2.5 Coupled-Cluster Single-Point Calculations for Tetroxides, Peroxyl Radicals, and Alkoxyl Radicals

Coupled-cluster single-point total energies were calculated for structures that were reasonably well described with single reference wave functions. These structures were the free peroxyl radicals, the intermediate tetroxides, the free alkoxyl radicals, and triplet molecular oxygen. The density functional theory (DFT)-optimized global minimum conformer geometries were used for all coupled-cluster calculations. The level-of-theory for coupled-cluster calculations was CCSD(T)-F12a/cc-pVDZ-F12,^56,57^ as implemented in Molpro.^58^ Some structures were too large to be calculated with canonical coupled-cluster, so those were calculated with DLPNO-CCSD(T)-F12/cc-pVTZ-F12,^59^ as implemented in ORCA-4.2.1.^60,61^ The DLPNO-CCSD(T)-F12 parameters were tuned to match the canonical CCSD(T)-F12a energies as closely as possible. This was done by calculating both the CCSD(T)-F12a/cc-pVDZ-F12 and DLPNO-CCSD(T)-F12 energies for the largest system, where CCSD(T)-F12a was still applicable. The only change that had any appreciable effect was the increase of the basis set size from the cc-pVDZ-F12 to cc-pVTZ-F12 for the DLPNO calculations.

A great deal of care was taken when choosing the Hartree-Fock (HF) reference wave function for coupled-cluster calculations. It is a well-known problem that HF calculations may converge to only local minima or even saddle-point solutions instead of the global minimum.^62,63^ To find the global minimum HF wave function, the original SCF solution was perturbed with a series of orbital mixings, and the perturbed wave function was then reoptimized.^62^ The lowest energy HF wave function was then used for the coupled-cluster calculations. Further details on this method can be found in the SI (Section S1).

### 2.6 Thermochemistry

The thermochemical parameters were computed for all tetroxides, peroxyl radicals, alkoxyl radicals, as well as triplet and singlet molecular oxygen, at the ωB97X-D/aug-cc-pVTZ level of theory. Vibrational analyses were carried out for the DFT-optimized global minimum structures, and the subsequent thermochemical data was calculated at 298.15 K and 1 atm pressure. Total Gibbs energies were calculated by adding the Gibbs energy correction from the thermochemistry calculation to the total energy calculated with CCSD(T)-F12a/cc-pVDZ-F12 or DLPNO-CCSD(T)-F12/cc-pVTZ-F12 level of theory. For the smallest systems, namely, HO•, HO_2_•, MeO•, MeO_2_•, and ^3^O_2_, improved total energies were also calculated with the W2–F12 thermochemical recipe.^64^ The vibrational frequencies used in the Gibbs energy correction term were scaled by a factor of 0.957, as is suggested for the ωB97X-D/aug-cc-pVTZ in the CCCDBD (Computational Chemistry Comparison and Benchmark Database) vibrational scaling factor database.^65^

In addition to DFT thermochemistry, vibrational analyses were carried out with CASSCF(5,4)/6-311++G(d,p) for RO_2_• and with CASSCF(10,8)/6-311++G(d,p) for the RO_2_···R′O_2_ cluster, the tetroxide formation transition state (ROO···OOR′)^‡^, the tetroxide minimum RO_4_R′, and the decomposition transition state (RO···O_2_···OR′)^‡^. All CASSCF thermochemical calculations were conducted using the corresponding CASSCF-optimized geometries at 298.15 K and 1 atm pressure. The vibrational frequencies used in the Gibbs energy correction term were scaled with a factor of 0.906, which is the suggested value for HF/6-311+G(3df,2pd), the closest level of theory to CASSCF/6-311++G(d,p) available in the CCCDBD database.^65^

## 3 Results and Discussion

### 3.1 Model Systems

The model systems for this study were chosen by considering various atmospheric reaction pathways that produce primary and secondary peroxyl radicals. Tertiary peroxyl radicals are believed to have slow self- and cross-reaction rates^17^ and are therefore not studied in this work. The question on the universality of the commonly assumed trend of slow rates for tertiary systems will be investigated in a follow-up study.

Simple alkylperoxyl radicals such as methyl-, ethyl-, and *i*-propylperoxyl radicals (MeO_2_•, EtO_2_•, *i*PrO_2_•) are derived from alkanes by hydrogen abstraction by HO• or NO_3_•, followed by the addition of O_2_. Acyl- and acetonyl peroxyl radicals (AcO_2_•, AceO_2_•) are similarly formed by hydrogen abstraction followed by O_2_ addition from acetaldehyde and acetone, respectively. The two hydroxylated peroxyls, *R-* and *S-*1-hydroxy-butan-2-ylperoxyl radicals (hereinafter denoted as *R*-BuOH-O_2_• and *S*-BuOH-O_2_•), are produced by HO• addition to the terminal unsaturated carbon of a 1-butene molecule, and subsequent addition of molecular oxygen to the second carbon, leading to formation of an enantiomeric mixture of *R* and *S* isomers. The two peroxyl radicals containing a nitrate group are similarly formed by addition of a nitrate radical to the terminal unsaturated carbon of 1-propene and subsequent addition of molecular oxygen, also producing an enantiomeric mixture of *R*- and *S-2-*peroxyl-propyl nitrate (hereinafter denoted as *R*-PrNO_3_-O_2_• and *S*-PrNO_3_-O_2_•). The hydroperoxyl radical (HO_2_•) is known to react predominantly via mechanisms other than the tetroxide pathway in the atmospheric conditions, but it was studied as the smallest possible model compound for tetroxide formation. The last of the studied systems, the allylperoxyl radical (AllylO_2_•), is not necessarily an atmospherically relevant model compound, but it is studied to see whether unsaturation near the peroxyl moiety makes a difference in the reactivity. Most of the studied tetroxides and the decomposition products thereof are formed from two identical peroxyl radicals, but three unsymmetric tetroxides, MeO_4_H, AcO_4_Me, and AceO_4_-*S*-BuOH, are also investigated. In addition, both *R,R-* and *R,S*-tetroxide products of *R*-BuOH-O_2_•, *S*-BuOH-O_2_•, *R*-PrNO_3_-O_2_•, and *S*-PrNO_3_• are considered.

### 3.2 Thermodynamics of Tetroxide Formation and Decomposition

The thermodynamics of the overall reaction was investigated by calculating ωB97X-D/aug-cc-pVTZ global minimum geometries and the corresponding vibrational frequencies for all RO•, RO_2_•, RO_4_R′, ^3^O_2_, and ^1^O_2_ structures (Table 1). Total energies for more accurate Gibbs energies were calculated with either CCSD(T)-F12a/cc-pVDZ-F12 or DLPNO-CCSD(T)-F12/cc-pVTZ-F12. Two decomposition channels were considered, one in which molecular oxygen is formed in the ground triplet state and another in which molecular oxygen is formed in an excited singlet state.

**Table 1:** Gibbs Energy Changes in the Tetroxide Formation and Decomposition^a^

		Gibbs energy change (Δ*G*), kcal/mol		
R^b^	R′^b^	RO_2_• + R′O_2_•	RO_4_R′	RO• + R′O• + ^3^O_2_
H	H	0.00	–5.15	3.43
Me	H	0.00	–2.52	–2.05
Me	Me	0.00	–1.48	–7.53
Et	Et	0.00	–1.31	–4.72
*i*Pr	*i*Pr	0.00	–1.08	–3.91
Ac	Me	0.00	–8.36	–12.70
Ac	Ac	0.00	–12.22	–17.88
Allyl	Allyl	0.00	–1.27	–8.15
Ace	Ace	0.00	–2.90	–9.67
Ace	Ace	0.00^c^	1.29^c^	–10.43^c^
Ace	*S*-BuOH	0.00^c^	–0.48^c^	–9.48^c^
*R*-BuOH	*R*-BuOH	0.00^c^	0.79^c^	–8.52^c^
*R*-BuOH	*S*-BuOH	0.00^c^	–0.09^c^	–8.52^c^
*R*-PrNO_3_	*R*-PrNO_3_	0.00^c^	0.25^c^	–8.17^c^
*R*-PrNO_3_	*S*-PrNO_3_	0.00^c^	0.01^c^	–8.24^c^

a Gibbs energies were calculated by adding Gibbs energy corrections (ωB97X-D/aug-cc-pVTZ, 298 K, 1 atm reference pressure) to the CCSD(T)-F12a/cc-pVDZ-F12 total energies.

b Data for R or R′ = H is presented for reference and comparison only: the dominant reaction pathway in these systems is not the mechanism studied here.

c Total energies have been calculated with DLPNO-CCSD(T)-F12/cc-pVTZ-F12 instead of CCSD(T)-F12a/cc-pVDZ-F12 for computational reasons.

The relative energy differences in the thermodynamic profile of the overall reaction (Table 1) were obtained by comparing the Gibbs energies of two separate peroxyl radicals to the energy of the tetroxide and to the total energy of two alkoxyl radicals and molecular oxygen. Thus, the energies listed in Table 1 describe the thermodynamic picture in the limit of no interaction between the various fragments. However, if molecular oxygen were to form in a singlet state, then the two alkoxyl radicals would form as doublets coupled into a singlet, thus allowing the possible direct formation of ROOR′ products, substantially lowering the total energy part of the Gibbs energy. This reaction route is still highly unlikely for all the studied systems because the decomposition into singlet molecular oxygen is very endergonic (^1^O_2_ is above the ^3^O_2_ in Gibbs energy by 29.61 kcal/mol at CCSD(T)-F12a/cc-pVDZ-F12 and by 32.10 kcal/mol at DLPNO-CCSD(T)-F12/cc-pVTZ-F12). In comparison, every reaction producing triplet molecular oxygen (except HO_2_• + HO_2_• → HO• + HO• + ^3^O_2_) is exergonic and therefore very likely to be the main reaction pathway.

The formation of the tetroxide intermediate is also thermodynamically feasible. For all systems where the total energy is calculated with the CCSD(T)-F12a method, the relative Gibbs energy change is negative for the tetroxide formation. For one tetroxide structure (AceO_4_Ace), both CCSD(T)-F12a and DLPNO-CCSD(T)-F12 total energies were calculated, and the canonical CCSD(T)-F12a suggests exergonic formation while DLPNO–CCSD(T)-F12 indicates slightly endergonic formation. As canonical CCSD(T)-F12a results are more accurate than DLPNO-CCSD(T)-F12 (DLPNO converges to canonical results if threshold values are infinitely tightened), this suggests that formation of tetroxide intermediates are an exergonic process for all the studied systems.

More accurate Gibbs energy changes were calculated for the three smallest systems with a modified explicitly correlated W2–F12 thermochemical protocol. The tetroxide intermediates were not calculated with this method. Results from these calculations are illustrated in Table 2 along with Gibbs energy changes calculated at the ωB97X-D/aug-cc-pVTZ and CCSD(T)-F12a/cc-pVDZ-F12 levels of theory. Increasing the accuracy appears to decrease the change in the Gibbs energy (i.e., the reaction free energies become less negative), except for the 2 HO_2_• → 2 HO• + O_2_ reaction, where such a trend is not observed (Table 2).

**Table 2:** Gibbs Energy Change of the Overall Reaction, Comparison of Methods

	Gibbs energy change (Δ*G*), kcal/mol		
reaction^a^	ωB97X-D/aug-cc-pVTZ	CCSD(T)-F12a/cc-pVDZ-F12^b^	W2–F12^c^
HO_2_• + HO_2_• → HO• + HO• + ^3^O_2_	5.94	3.43	4.88
MeO_2_• + HO_2_• → MeO• + HO• + ^3^O_2_	–2.17	–2.05	–0.84
MeO_2_• + MeO_2_• → MeO• + MeO• + ^3^O_2_	–10.28	–7.53	–6.56

a Data for reactions with HO_2_• is presented for reference and comparison only: the dominant reaction pathway in these systems is not the mechanism studied here.

b Thermal corrections to CCSD(T)-F12a/cc-pVDZ-F12 total energies were calculated at ωB97X-D/aug-cc-pVTZ.

c Geometries and frequencies were calculated with ωB97X-D/aug-cc-pVTZ instead of B3LYP/cc-pV(T+d)Z. Gibbs energy correction was scaled with a factor of 0.957.

Another motivation for producing CCSD(T)-F12a/cc-pVDZ-F12 total energies at ωB97X-D/aug-cc-pVTZ optimized geometries for peroxyl radicals and alkoxyl radicals is the apparent discrepancy between CASSCF and XMC-QDPT2 results (Sections 3.3 and 3.4) for separated peroxyl radicals and for the dissociation products of tetroxides. CASSCF predicts much smaller electronic energy differences between separated peroxyl radicals and tetroxides than coupled cluster does. Because peroxyl radicals and alkoxyl radicals are well described in the single reference DFT-formalism, the coupled-cluster corrected DFT results are probably more accurate than those from CASSCF or even from XMC-QDPT2 for the thermodynamics of the overall reaction. These energies are shown for all the studied systems in the SI (Section S9).

### 3.3 CASSCF(10,8)/6-311++G(d,p) Optimized Reaction Pathway

The whole reaction pathway starting from two separated peroxyl radicals (separated by 15 Å) all the way to the decomposition into two alkoxyl radicals and triplet molecular oxygen was studied at the CASSCF(10,8)/6-311++G(d,p) level of theory. The computational details, including the setup of the active space, are discussed in the SI (Section S2), and the results from the reaction pathway optimizations are shown in Table 3.

**Table 3:** CASSCF(10,8) Optimized Stationary Points and XMC-QDPT2(10,8) Single-Point Energies along the Reaction Coordinate^a^

		relative energy difference, kcal/mol					
R^b^	R′^b^	RO_2_• + R′O_2_•^c^	RO_2_···R′O_2_	[ROO···OOR′]^‡^	RO_4_R′	[RO···O_2_···OR′]^‡^	RO• + R′O• + ^3^O_2_
H	H	3.92 **8.42** (19.17)	–0.32 **2.95**	4.19 **4.13**	0.00	2.34 **1.71**	–12.46 **9.57** (33.10)
Me	H	3.09 **10.88** (16.72)	–1.23 **4.06**	3.87 **4.65**	0.00	1.21 **1.03**	–16.63 **12.62** (27.22)
Me	Me	0.88 **3.72** (16.19)	–1.27 **–0.56**	1.40 **–3.00**	0.00	2.31 **–3.42**	–17.41 **3.98** (23.26)
Et	Et	0.69 **10.71** (15.14)	–1.73 **5.38**	1.53 **–0.30**	0.00	1.21 **–0.74**	–18.37 **13.64** (24.41)
*i*Pr	*i*Pr	–0.47 **6.98** (15.24)	–2.92 **1.23**	1.06 **–3.69**	0.00	2.97 **–5.40**	–17.17 **12.08** (25.06)
Ac	Me	7.48 **19.37** (22.33)	4.66 **15.82**	d	0.00	1.09 **–0.44**	–20.33 **14.30** (23.49)
Ac	Ac	12.23 **32.04** (27.43)	8.66 **26.33**	d	0.00	0.05 **–0.99**	–23.94 **16.59** (22.68)
Allyl	Allyl	1.31 **14.30** (16.52)	–1.06 **10.41**	2.73 **3.67**	0.00	0.71 **0.32**	–20.17 **18.02** (23.51)
Ace	Ace	2.36 **19.65** (17.94)	–0.73 **14.24**	3.41 **5.10**	0.00	0.91 **0.72**	–22.92 **18.66** (22.75)
Ace	*S*-BuOH	3.67 **21.28** (16.67)^e^	–1.64 **13.48**	3.26 **3.58**	0.00	1.35 **–2.82**	–21.49 **45.69** (22.30)^e^
*R*-BuOH	*R*-BuOH	3.02 **21.55** (14.56)^e^	–2.40 **13.45**	6.64 **6.76**	0.00	1.11 **–3.25**	–21.86 **47.58** (21.80)^e^
*R*-BuOH	*S*-BuOH	3.57 **20.00** (15.24)^e^	–1.57 **14.26**	3.52 **2.52**	0.00	1.36 **–3.65**	–20.87 **18.70** (22.49)^e^
*R*-PrNO_3_	*R*-PrNO_3_	3.44 **28.03** (15.27)^e^	–1.82 **18.61**	3.72 **5.08**	0.00	1.46 **–5.11**	–22.52 **50.56** (22.02)^e^
*R*-PrNO_3_	*S*-PrNO_3_	4.02 **28.40** (14.94)^e^	–1.32 **17.50**	2.73 **2.14**	0.00	2.18 **–4.59**	–21.65 **25.09** (21.78)^e^

a Lightfaced values correspond to CASSCF energies, and bold values correspond to XMC-QDPT2 single-point energies. For comparison, values in brackets correspond to CCSD(T)-F12a/cc-pVDZ-F12 single-point total energies at ωB97X-D/aug-cc-pVTZ optimized geometries.

b Data for R or R′ = H is presented for reference and comparison only: the dominant reaction pathway in these systems is not the mechanism studied here.

c In the geometry optimizations, the distance between the two terminal oxygen atoms of the peroxyl moieties were frozen to 15 Å.

d Barrierless formation reaction.

e DLPNO-CCSD(T)-F12/cc-pVTZ-F12 used instead of CCSD(T)-F12a/cc-pVDZ-F12.

All the systems studied have local minima corresponding to a loosely bound peroxyl radical pair (reactant complex; RO_2_···R′O_2_) which is lower in electronic energy than the separated pair of peroxyl radicals (RO_2_• + R′O_2_•). At the CASSCF level, this loosely bound cluster is lower in energy than the tetroxide intermediate in all systems, except for the AcO_2_···AcO_2_ and AcO_2_···MeO_2_ clusters, where the tetroxides are lower in energy by 8.66 and 4.66 kcal/mol, respectively. These two systems are also unique in that the formation reaction of the tetroxide from the peroxyl pair cluster appears to be barrierless. A relaxed surface scan starting from the tetroxide intermediate and lengthening the inner O–O bond showed that the electronic energy increases as a function of the separation of the oxygen atoms. This behavior was found originally only for AcO_2_• + AcO_2_•; the nonidentical pair of AcO_2_• + MeO_2_• was studied afterward to see whether only one acylperoxyl radical is enough to make the formation reaction barrierless. The prereactive complex of these two systems corresponds to an inflection point on the potential energy surface rather than a true minimum point in a potential well. AcO_2_• + AcO_2_• and AcO_2_• + MeO_2_• also have the highest excess energy in comparison to the tetroxide intermediate (i.e., the tetroxide is the most strongly bound compared to the free reactants). For all the other studied systems, a transition state for the formation of the tetroxide was found, with barrier heights (total energies, compared to the reactant complex) ranging from 2.67 to 9.04 kcal/mol. The highest barrier corresponds to the formation of the *R*-BuOH-O_4_-*R*-BuOH—all other barrier heights range from 2.67 to 5.54 kcal/mol.

**Figure 1:**
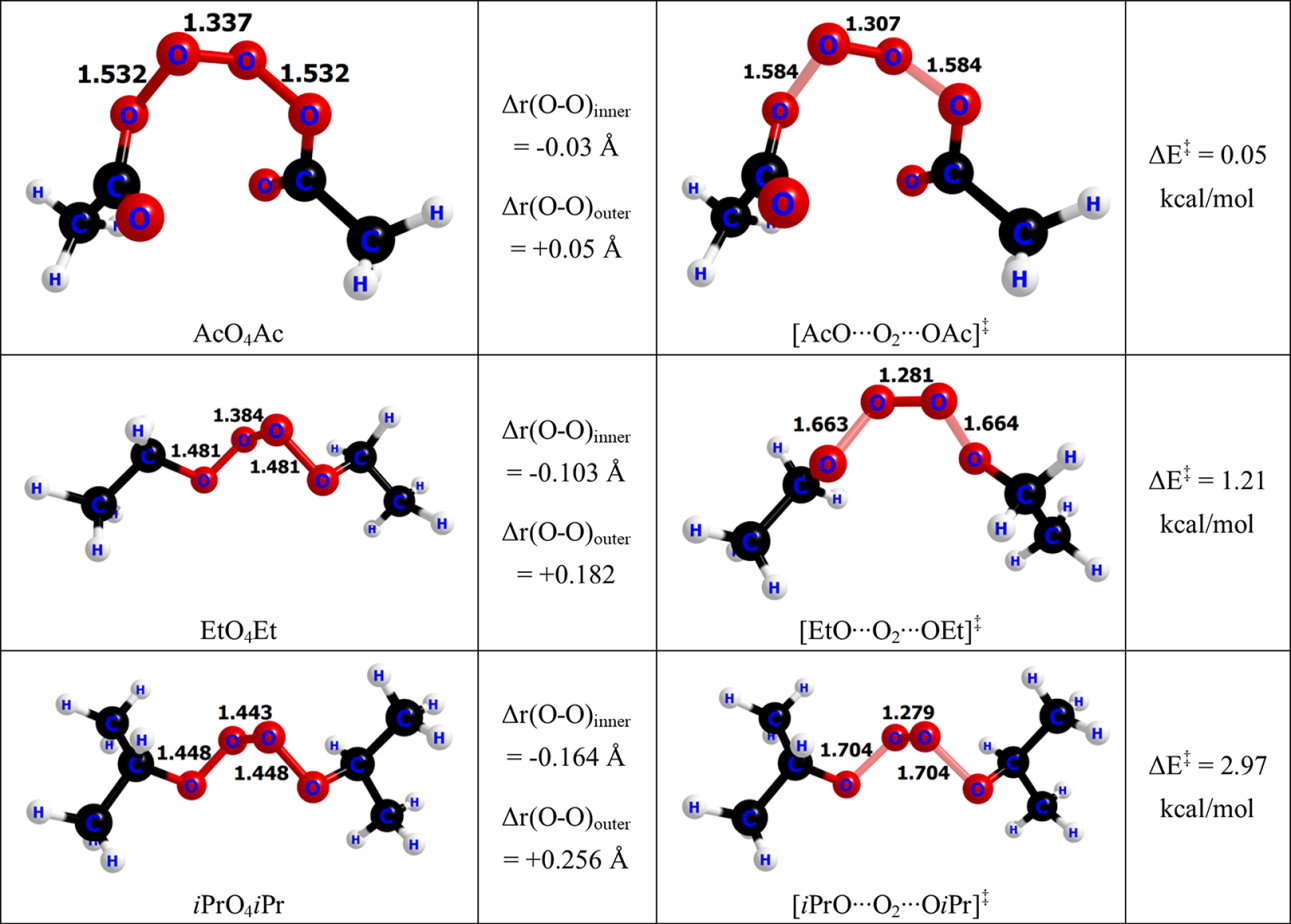
Changes in the different O–O bond lengths in the tetroxide decomposition and their relation to the CASSCF barrier height.

The barrier heights for the decomposition reaction are smaller than those for the formation reaction. The decomposition barriers range from 0.05 to 2.97 kcal/mol. The lowest decomposition barrier is found for [AcO···O_2_···OAc]^‡^, while the highest barrier is found for [*i*PrO···O_2_···O*i*Pr]^‡^. Differences between the barrier heights can be attributed to changes in O–O bond lengths that the tetroxide undergoes to decompose. For example, the O–O bond lengths in the AcO_4_Ac change very little when going from the tetroxide intermediate to the decomposition transition state geometry [AcO···O_2_···OAc]^‡^, whereas the changes are more pronounced between the *i*PrO_4_*i*Pr tetroxide and the [*i*PrO···O_2_···O*i*Pr]^‡^ transition state geometry. This trend is displayed in Figure 1.

**Figure 2:**
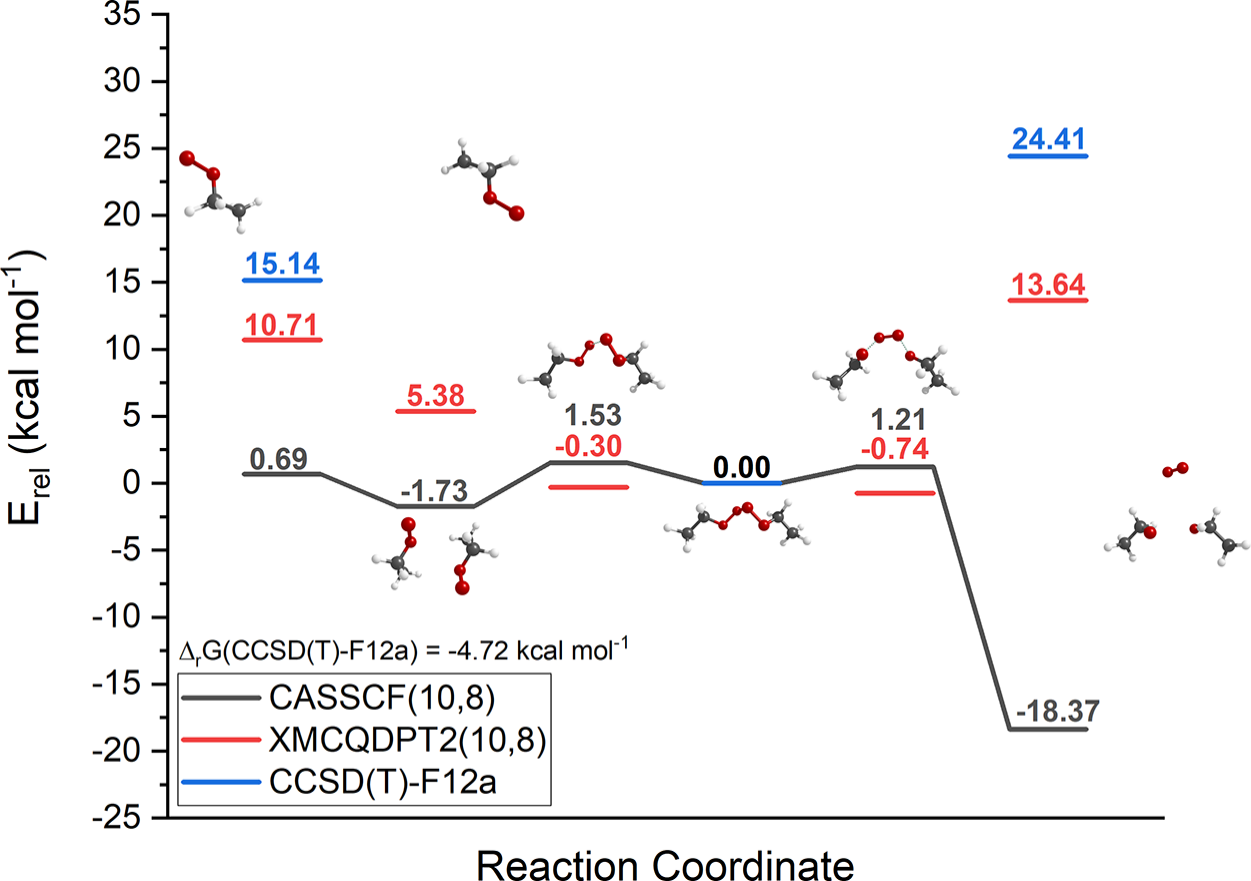
CASSCF optimized reaction pathway (gray) with XMC-QDPT2 single-point energies (red) and CCSD(T)-F12a single-point energies (blue) (CCSD(T)-F12a energies are calculated on DFT-optimized geometries). Reaction of EtO_4_Et shown as an example.

The steep decrease in relative total energies from the tetroxides to the RO• + R′O• + ^3^O_2_ products at the CASSCF level is not replicated in XMC-QDPT2 and coupled-cluster calculations (see Table 3 and Figure 2). Both XMC-QDPT2 and coupled-cluster results suggest a rise in total energy in comparison to the decomposition transition state. The decomposition of a tetroxide into two radicals and molecular oxygen is most likely an endothermic process, so the coupled-cluster and XMC-QDPT2 total energies for the products are arguably also more chemically reasonable. However, the entropic boost of forming three product molecules from two reactants still drives the overall reaction to be exergonic, as demonstrated in the previous section (Table 1). Additionally, it has been demonstrated in our previous studies that the subsequent reactions of the alkoxyl radicals, including both H-shifts, dissociation, and recombination following intersystem crossings (Scheme 1, R1–R3) all have extremely high reaction rates.

### 3.4 XMC-QDPT2(10,8)/6-311++G(d,p) Single-Point Energies

**Figure 3:**
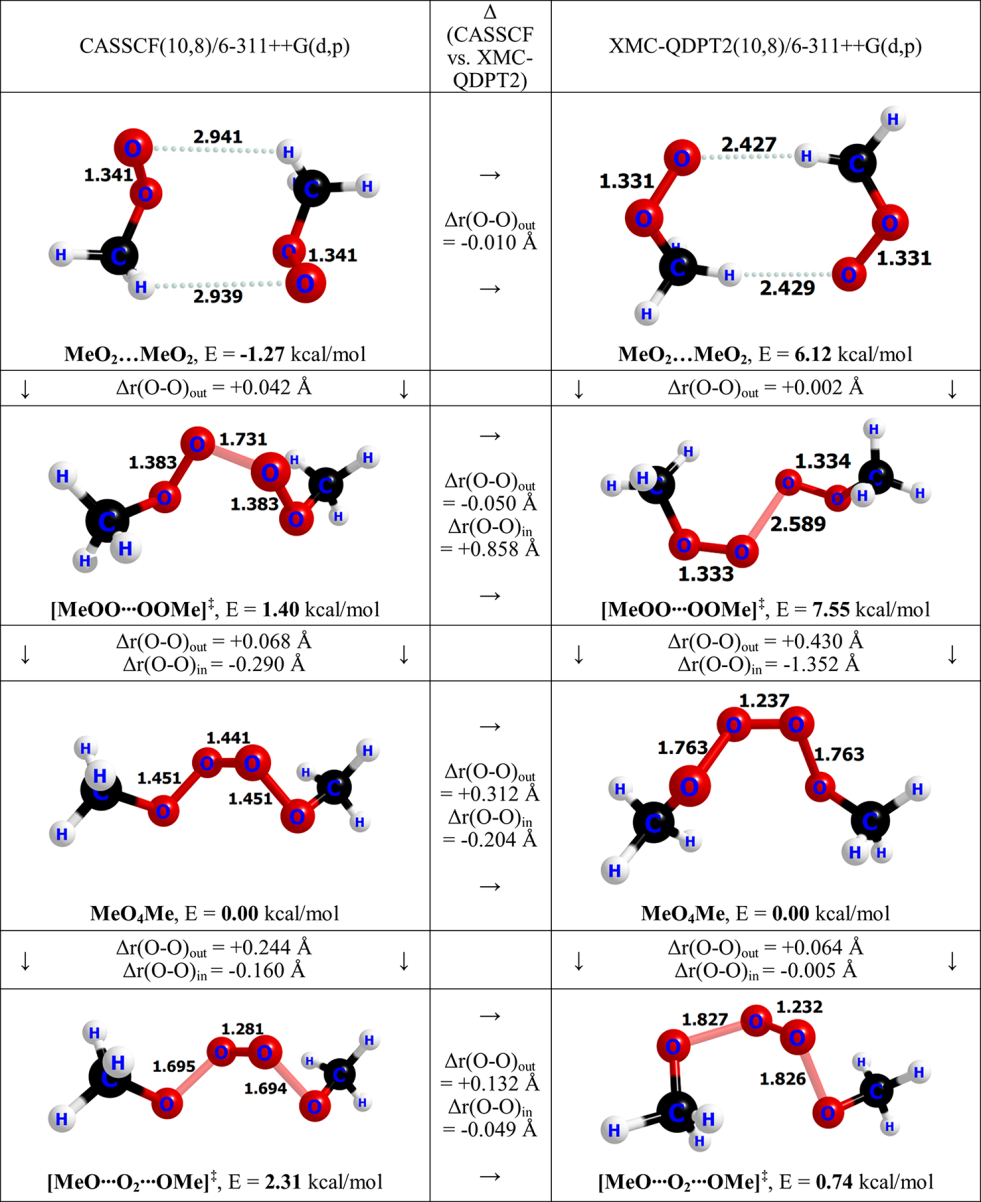
Comparison of geometry optimizations using CASSCF and XMC-QDPT2. XMC-QDPT2 structures and energies are reproduced from our previous work.^32^

CASSCF energies were corrected with XMC-QDPT2 single-point energy calculations using the CASSCF-optimized molecular geometries. The motivation for calculating XMC-QDPT2 energies was to alleviate the lack of dynamical correlation in CASSCF. As discussed above, although the XMC-QDPT2 corrections do not appear to be fully compatible with CASSCF geometries, they provide useful further insights into the reactions. The XMC-QDPT2 single-point energies predict the transition states, for both the formation and the decomposition of the tetroxide, to be lower in energy than the tetroxide itself, in multiple studied systems (see boldfaced values in Table 3, and Figure 2). This result is in apparent contradiction with our previous work, in which we found qualitatively similar reaction pathways for MeO_4_Me formation and decomposition at the XMC-QDPT2(10,8)/6-311++G(d,p) and CASSCF(10,8)/6-311++G(d,p) levels of theory. The crucial difference is that in our previous work, the molecular geometries of the MeO_4_Me were also optimized at the XMC-QDPT2(10,8)/6-311++G(d,p) level of theory. When this is done, the barrier heights are comparable between the methods, with formation barriers of 2.67 and 1.43 kcal/mol and decomposition barriers of 2.31 and 0.74 kcal/mol using CASSCF and XMC-QDPT2, respectively (Figure 3). Unfortunately, the extremely high cost of XMC-QDPT2 geometry optimizations prevents us from carrying them out for the larger systems studied here.

Even though the XMC-QDPT2 single-point transition states are lower in total energy than the tetroxide intermediates, the formation transition state is consistently higher in energy than the decomposition transition state, across all studied systems. This agrees qualitatively with both the CASSCF optimized reaction pathways and the XMC-QDPT2 optimized reaction pathway for the MeO_4_Me reaction. All obtained results thus indicate that the formation of the tetroxide is the rate-limiting step in the total reaction pathway, and the barrier for this formation step is unlikely to be insurmountably high at least for primary and secondary peroxyl radicals. The latter finding is further verified by ASCI-SCF single-point energy calculations (SI Section S7) on the MeO_4_Me system.

The XMC-QDPT2 optimizations reported in our previous study suggest that the loosely bound MeO_2_···MeO_2_ reactant complex is higher in energy than the tetroxide, while the CASSCF results of this work indicate that the reactant complexes are generally lower in energy than the tetroxide (Figures 2 and 3).

The absolute differences in relative energies with respect to the tetroxide intermediate (Table 3) between CASSCF and XMC-QDPT2 are largest for the stationary points where interactions are noticeably different from those in the tetroxide, due to either the presence of long-range interactions (RO_2_···R′O_2_ and RO···O_2_···R′O) or the complete separation of the fragments (RO_2_• + R′O_2_•). For RO_2_• + R′O_2_•, RO_2_···R′O_2_, and RO···O_2_···R′O, the average of energy differences between the methods are 14.15, 11.56, and 41.78 kcal mol^–1^, respectively. For the two transition states, [ROO···OOR′]^‡^ and [RO···O_2_···OR′]^‡^, the relative difference between CASSCF and XMC-QDPT2 is much smaller, 1.48, and 2.73 kcal/mol, respectively.

The difference between CASSCF and XMC-QDPT2 for describing the loosely bound structures can be attributed to the lack of dispersion in CASSCF.^66^ Differences in the transition state energies and tetroxide energies may be caused by the differences in optimal O–O bond lengths between the methods. The comparison of the CASSCF optimized molecular geometries from this study and the XMC-QDPT2 optimized molecular geometries from our previous study for MeO_4_Me formation and decomposition^32^ reveal drastically different O–O bond lengths along the reaction pathway (Figure 3).

Additionally, the slight differences in optimal bond lengths and in the description of long-range interactions between the methods lead to different conformations in the optimized structures. To provide more rigorous comparison of the methods, we tried to reoptimize CASSCF-geometries for MeO_4_Me stationary points with XMC-QDPT2 and *vice versa*. Unfortunately, no convergence to directly comparable structures were obtained by either approach.

### 3.5 Bimolecular Reaction Rates

The overall bimolecular reaction rate coefficients were calculated using elementary transition state theory (TST) and CASSCF energetics. The computed reaction rate coefficients are predominantly smaller than the available experimental values (SI Table S4). The AcO_4_Ac system is the exception to this trend, as the calculated rate slightly exceeds the experimental values. (The calculated rate also exceeds the gas-kinetic collision rate, implying that the conventional TST rate expression is not applicable for this case). For the other studied systems, the smaller rate coefficients may be due to the substantial underestimation of the excess total energies of the peroxyl radicals (as compared to both the tetroxides and especially the formation transition states) at the CASSCF level of theory. As can be seen in Table 3, the coupled-cluster calculations suggest systematically higher electronic energies for the peroxyl radicals, which in turn would lower the Δ*G*^‡^ correspondingly and enhance the reaction rate.

As discussed above, our results strongly indicate that the rate-limiting step for the studied RO_2_ + R′O_2_ reactions is the formation of either the RO_4_R′ tetroxide or the RO_2_···R′O_2_ complex. While the transition state corresponding to decomposition of the RO_4_R′ cannot be modeled without multireference methods, the transition state for tetroxide formation can be qualitatively described also by DFT, for example. Thus, reasonable estimates of the overall reaction rates could plausibly be obtained by combining master equation modeling with coupled-cluster corrected DFT results on the RO_4_R′ tetroxide and its formation TS, as well as by long-range transition state theory^67^ for treating RO_2_···R′O_2_ formation. We performed such modeling on systems for which experimental data is available and for which accurate canonical CCSD(T)-F12a energy calculations could be performed, using the MESMER program (see details in SI section S5).^68^ The overall reaction rates obtained by this approach (SI Table S5) are generally even closer to the experimental values than those obtained using CASSCF energetics, and the ordering of the rates is fully correct: *k*(iPrO_4_iPr) < *k*(EtO_4_Et) < *k*(MeO_4_Me) < *k*(AceO_4_Ace) < *k*(AcO_4_Me) < *k*(AcO_4_Ac). The errors are largest for the fastest reactions, presumably due to the limitations in modeling barrierless formation of tetroxides from the RO_2_···R′O_2_ complexes. We demonstrated in a very recent study^69,70^ that the experimental rates for RO_2_ + R′O_2_ reactions with submerged barriers can be predicted surprisingly well simply based on RO_2_···R′O_2_ complex lifetimes estimated from nonreactive classical molecular dynamics simulations. These two sets of results now provide a complete toolbox for estimating at least order-of-magnitude accuracy rates for overall RO_2_···R′O_2_ reactions in both the presence and the absence of substantial barriers for tetroxide formation. Unfortunately, predicting quantitative branching ratios for the various product channels will still require substantial further work.

## 4 Conclusions and Atmospheric Implications

We have investigated the formation and decomposition pathways for tetroxide intermediates formed in the recombination of multiple atmospherically relevant primary and secondary peroxyl radical model compounds at the CASSCF/6-311++G(d,p) level of theory. We showed that the barrier heights for both formation and decomposition are small in comparison to the excess energy of the peroxyl radicals, suggesting that the studied reaction mechanism is a plausible pathway for the formation and decomposition of tetroxide intermediates in the atmosphere. XMC-QDPT2 single-point energy corrections agree with CASSCF in that the formation transition state is higher in energy than the decomposition transition state, which in turn suggests that the formation of the tetroxide (or in some cases that of the RO_2_···R′O_2_ complex) is likely the rate-limiting step of the total reaction.

We observed several qualitative discrepancies between CASSCF results and XMC-QDPT2 single-point energies. A comparison of optimized reaction pathways for MeO_4_Me suggests that the discrepancies are likely due to the lack of XMC-QPDT2 optimizations caused by computational limitations. In future research on similar systems, it would be beneficial to employ methods with a better description of both static and dynamical correlation, and especially weak interactions such as H-bonds, in the geometry optimizations. At the time of this study, full geometry optimizations with, for example, XMC-QDPT2, ASCI-SCF(PT2), or multireference DFT methods are not yet widely applicable for large systems.

Thermodynamic calculations at the DFT+CCSD(T) level indicate that even though the product alkoxyl radicals are likely to be higher in total energy than the tetroxide intermediates, the overall reaction is spontaneous with respect to the Gibbs energy change for all studied systems, except for the model system HO_2_• + HO_2_•, for which other reaction channels are in any case known to dominate. We have also demonstrated in our previous research that the alkoxyl radical products react readily via hydrogen-shift or recombination reactions, which terminate the radicals and lower the total energies considerably. Furthermore, based on the mechanistic insights gained from our multireference calculations—in particular, the rate-limiting nature of the tetroxide formation step—we were able to estimate bimolecular overall reaction rate coefficients also at the DFT+CCSD(T) level of theory. These were consistent and comparable to experimental data, which further substantiates the studied mechanism.

## Supporting Information Available

Details of orbital rotation method for coupled-cluster calculations, active space generation and orbital visualization, size-consistency analysis of CASSCF and XMC-QDPT2, thermodynamic data for bimolecular reaction rate coefficients and elementary TST rate coefficient calculations, master equation modeled reaction rates, discussion of ASCI-SCF results, reaction coordinate graphs, *xyz*-coordinates, and total energy of various studied systems (PDF)

## Notes

**Data Available**: The data underlying this study (output-files of quantum chemical calculations) are openly available in “Supplementary Data for ‘Gas-phase Peroxyl Radical Recombination Reactions: A Computational Study of Formation and Decomposition of Tetroxides’” at 10.5281/zenodo.6517406.
