# Supplementary material for: Gas-Phase Peroxyl Radical Recombination Reactions: A Computational Study of Formation and Decomposition of Tetroxides
Source: J Phys Chem A. 2022 Jun 16;126(25):4046–56. doi: 10.1021/acs.jpca.2c01321 (PMC9251773; doi:10.1021/acs.jpca.2c01321)
Supplement: Supplementary file 1 — jp2c01321_si_001.pdf [file jp2c01321_si_001.pdf]

Supporting Information for Publication

Gas-phase Peroxyl Radical Recombination Reactions: A Computational Study of Formation and Decomposition of Tetroxides

Vili-Taneli Salo,[*,†] Rashid Valiev,[†] Susi Lehtola, [†,‡] Theo Kurtén[*,†]

[†]Department of Chemistry, Faculty of Science, University of Helsinki, Helsinki FI-00014, Finland

[‡]Molecular Sciences Software Institute, Blacksburg, Virginia 24061, United States

Table of Contents

S1. Global minimum HF-SCF solution for coupled-cluster calculations: Orbital rotation method S7

S1.1. Orbital rotations for CCSD(T)-F12/cc-pVDZ-F12 calculations S7

S1.2. Orbital rotations for DLPNO-CCSD(T)-F12/cc-pVTZ-F12 calculations S7

S2. Generation of the CASSCF active spaces for the studied structures along the reaction coordinate S8

S3. Size-consistency analysis of CASSCF and XMCQDPT2 ....S10

Table S1. Size-consistency analysis of CASSCF and XMCQDPT2. .... S10

S4. Thermodynamic data for bimolecular rate coefficient calculations....................S11

Table S2. Gibbs energy changes, calculated with CASSCF/6-311++G(d,p). S11

Table S3. Reaction enthalpy changes, calculated with CASSCF/6-311++G(d,p). S11

S5. TST reaction rates based on CASSCF energetics S13

Table S4. Bimolecular reaction rate coefficients, calculated from CASSCF Gibbs energy changes. S13

S6. Master equation modeling of selected reaction rates based on CCSD(T) and DFT energetics S15
S6.1. General simulation parameters S16
Table S5. Type definitions of the stationary points in MESMER simulations. S16
S6.2. Barrierless association (**ILT**) S16
S6.3. Unimolecular isomerization (**RRKM**) S17
S6.4. Irreversible dissociation (***r*ILT**) S18
S.6.5. Simulated reaction profiles (**ILT+RRKM+*r*ILT**) S18
Table S6. Bimolecular reaction rate coefficients from MESMER simulations. S18
S7. ASCI-SCF(PT2) results S19
Table S7. ASCI-SCF(PT2)/cc-pVDZ relative electronic energies in kcal mol^{-1} for HO_4H, MeO_4H, and MeO_4Me S19
Table S8. ASCI-SCF(PT2)/cc-pVTZ relative electronic energies in kcal mol^{-1} for HO_4H, MeO_4H, and MeO_4Me S20
Table S9. ASCI-SCF(PT2)/6-311++G(d,p) relative electronic energies in kcal mol-1 for HO_4H, MeO_4H, and MeO_4Me. S20
S8. Reaction coordinate graphs S25
S9. ωB97X-D / aug-cc-pVTZ Optimized Geometries S33
S9.1. Molecular oxygen S33
$^3O_2$ ($^3\Sigma_g^-$) S33
$^1O_2$ ($^1\Delta_g$) S33
S9.2. Alkoxyl radicals S33
HO (hydroxyl) S33
MeO (methoxyl) S33
EtO (ethoxyl) S33
*i*PrO (isopropoxyl) S34
AcO (acetyloxyl) S34
AceO (acetonyloxyl) S34
AllylO (allyloxyl) S35
R-BuOH-O (*R*-1-hydroxy-butan-2-yloxyl) S35
S-BuOH-O (*S*-1-hydroxy-butan-2-yloxyl) S35
R-PrNO3-O (*R*-2-oxyl-propyl nitrate) S36
S-PrNO3-O (*S*-2-oxyl-propyl nitrate) S36
S9.3. Peroxyl radicals S37
HO_2 (hydroperoxyl) S37
MeO_2 (methylperoxyl) S37
EtO_2 (ethylperoxyl) S37
iPrO_2 (isopropylperoxyl) S37
AcO_2 (acetylperoxyl) S38
$AceO_2$ (acetonylperoxyl) S38
$AllylO_2$ (allylperoxyl) S38
R-BuOH-O_2 (*R*-1-hydroxy-butan-2-ylperoxyl) S39
S-BuOH-O_2 (*S*-1-hydroxy-butan-2-ylperoxyl) S39

R-$PrNO_3$-O_2 (*R*-2-peroxyl-propyl nitrate) S39
S-$PrNO_3$-O_2 (*S*-2-peroxyl-propyl nitrate) S40

S9.4. Pre-reactive complexes........ S40

MeO_2…MeO_2 .... S40
EtO_2…EtO_2........ S41
iPrO_2…iPrO_2 .... S41
AceO_2…AceO_2........ S42

S9.5. Formation transition state structures S42

[MeOO···OOMe]‡ S42
[EtOO···OOEt]‡........ S43
[iPrOO···OOiPr]‡ S43
[AceOO···OOAce]‡........ S44

S9.6. Tetroxides........ S44

HO_4H (tetraoxidane)........ S44
MeO_4H (1-methyltetraoxidane)........ S44
MeO_4Me (1,4-dimethyltetraoxidane) S45
EtO_4Et (1,4-diethyltetraoxidane)........ S45
*i*PrO_4*i*Pr (1,4-diisopropyltetraoxidane)........ S45
AcO_4Me (1-(methyltetraoxidaneyl)ethan-1-one) S46
AcO_4Ac (1,4-diacetyltetraoxidane) S46
AllylO_4Allyl (1,4-diallyltetraoxidane)........ S47
AceO_4Ace (1,1’-tetraoxidanediylbis(propan-2-one)) S47
AceO_4-*S*-BuOH (*S*-1-((1-hydroxybutan-2-yl)tetraoxidaneyl)propan-2-one) S48
R-BuOH-O_4-*R*-BuOH ((2*R*,2’*R*)-2,2’-tetraoxidanediylbis(butan-1-ol))........ S49
R-BuOH-O_4-*S*-BuOH ((2*R*,2’S)-2,2’-tetraoxidanediylbis(butan-1-ol)) S49
R-$PrNO_3$-O_4-*R*-$PrNO_3$ ((2*R*,2’*R*)-tetraoxidanediylbis(propane-2,1-diyl) dinitrate)........ S50
R-$PrNO_3$-O_4-*S*-$PrNO_3$ ((2*R*,2’*S*)-tetraoxidanediylbis(propane-2,1-diyl) dinitrate) S51

S10. CASSCF / 6-311++G(d,p) Optimized Geometries S52

HO_4H (tetraoxidane)........ S52

HO_2 .... S52
HO_2 + HO_2........ S52
HO_2…HO_2 .... S52
[HOO···OOH]‡........ S52
HO_4H S53
[HO···O_2···OH]‡ S53
HO…O_2…HO S53

MeO_4H (1-methyltetraoxidane)........ S54

MeO_2........ S54
HO_2 .... S54
MeO_2 + HO_2 .... S54
MeO_2…HO_2........ S54
[MeOO···OOH]‡ S55
MeO_4H........ S55
[MeO···O_2···OH]‡ S55
MeO…O_2…HO S56

MeO_4Me (1,4-dimethyltetraoxidane) S56

MeO_2 ... S56
$MeO_2 + MeO_2$ ... S56
$MeO_2 \ldots MeO_2$ ... S56
[MeOO···OOMe]‡ ... S57
MeO_4Me ... S57
[MeO···O_2···OMe]‡ ... S58
MeO...O_2...MeO ... S58

EtO_4Et (1,4-diethyltetraoxidane) ... S59

EtO_2 ... S59
$EtO_2 + EtO_2$ ... S59
$EtO_2 \ldots EtO_2$ ... S59
[EtOO···OOEt]‡ ... S60
EtO_4Et ... S60
[EtO···O_2···OEt]‡ ... S61
EtO…O_2…EtO ... S61

*i*PrO$_4$*i*Pr (1,4-diisopropyltetraoxidane) ... S62

iPrO_2 ... S62
iPrO_2 + iPrO_2 ... S62
iPrO_2…*i*PrO_2 ... S63
[*i*PrOO···OO*i*Pr]‡ ... S63
*i*PrO$_4$*i*Pr ... S64
[*i*PrO···O_2···O*i*Pr]‡ ... S64
*i*PrO…O_2…*i*PrO ... S65

AcO_4Me (1-(methyltetraoxidaneyl)ethan-1-one) ... S66

AcO_2 ... S66
MeO_2 ... S66
$AcO_2 + MeO_2$ ... S66
$AcO_2 \ldots MeO_2$ ... S66
AcO_4Me ... S67
[AcO···O_2···OMe]‡ ... S67
AcO…O_2…MeO ... S67

AcO_4Ac (1,4-diacetyltetraoxidane) ... S68

AcO_2 ... S68
$AcO_2 + AcO_2$ ... S68
$AcO_2 \ldots AcO_2$ ... S68
AcO_4Ac ... S69
[AcO···O_2···OAc]‡ ... S69
AcO…O_2…AcO ... S70

$AllylO_4Allyl$ (1,4-diallyltetraoxidane) ... S70

$AllylO_2$ ... S70
$AllylO_2 + AllylO_2$ ... S70
$AllylO_2 \ldots AllylO_2$ ... S71
[AllylOO···OOAllyl]‡ ... S71
$AllylO_4Allyl$ ... S72
[AllylO···O_2···OAllyl]‡ ... S72
AllylO…O_2…AllylO ... S73

$AceO_4Ace$ (1,1’-tetraoxidanediylbis(propan-2-one)) ... S73

$AceO_2$ S73
$AceO_2$ + $AceO_2$ S74
$AceO_2$…$AceO_2$ S74
[AceOO···OOAce]‡ S75
$AceO_4Ace$ S75
[AceO···O_2···OAce]‡ S76
AceO…O_2…AceO S77

$AceO_4$-*S*-BuOH (*S*-1-((1-hydroxybutan-2-yl)tetraoxidaneyl)propan-2-one) S77

$AceO_2$ S77
S-BuOH-O_2 .... S77
$AceO_2$ + *S*-BuOH-O_2 .... S78
$AceO_2$…*S*-BuOH-O_2 .... S78
[AceOO···OO-*S*-BuOH]‡ S79
$AceO_4$-*S*-BuOH S79
[AceO···O_2···O-*S*-BuOH]‡ S80
AceO…O_2…*S*-BuOH-O S81

R-BuOH-O_4-*R*-BuOH ((2*R*,2’*R*)-2,2’-tetraoxidanediylbis(butan-1-ol)) S81

R-BuOH-O_2 .... S81
R-BuOH-O_2 + *R*-BuOH-O_2 .... S82
R-BuOH-O_2…*R*-BuOH-O_2 .... S83
[*R*-BuOH-OO···OO-*R*-BuOH]‡ S83
R-BuOH-O_4-*R*-BuOH S84
[*R*-BuOH-O···O_2···O-*R*-BuOH]‡ S85
R-BuOH-O…O_2…*R*-BuOH-O S86

R-BuOH-O_4-*S*-BuOH ((2*R*,2’S)-2,2’-tetraoxidanediylbis(butan-1-ol)) S86

R-BuOH-O_2 .... S86
S-BuOH-O_2 .... S86
R-BuOH-O_2 + *S*-BuOH-O_2 .... S86
R-BuOH-O_2…*S*-BuOH-O_2 .... S87
[*R*-BuOH-OO···OO-*S*-BuOH]‡ S88
R-BuOH-O_4-*S*-BuOH S89
[*R*-BuOH-O···O_2···O-*S*-BuOH]‡ S89
R-BuOH-O…O_2…*S*-BuOH-O S90

R-$PrNO_3$-O_4-*R*-$PrNO_3$ ((2*R*,2’*R*)-tetraoxidanediylbis(propane-2,1-diyl) dinitrate) S91

R-$PrNO_3$-O_2 .... S91
R-$PrNO_3$-O_2 + *R*-$PrNO_3$-O_2 .... S91
R-$PrNO_3$-O_2…*R*-$PrNO_3$-O_2 .... S92
[*R*-$PrNO_3$-OO···OO-*R*-$PrNO_3$]‡ S93
R-PrNO3-O_4-*R*-$PrNO_3$ S94
[*R*-$PrNO_3$-O···O_2···O-*R*-$PrNO_3$]‡ S94
R-$PrNO_3$-O…O_2…*R*-$PrNO_3$-O S95

R-$PrNO_3$-O_4-*S*-$PrNO_3$ ((2*R*,2’*S*)-tetraoxidanediylbis(propane-2,1-diyl) dinitrate) S96

R-$PrNO_3$-O_2 .... S96
S-$PrNO_3$-O_2 .... S96
R-$PrNO_3$-O_2 + *S*-$PrNO_3$-O_2 .... S96
R-$PrNO_3$-O_2…*S*-$PrNO_3$-O_2 .... S97
[*R*-$PrNO_3$-OO···OO-*S*-$PrNO_3$]‡ S97

R-$PrNO_3$-O_4-*S*-$PrNO_3$ S98
[*R*-$PrNO_3$-O···O_2···O-*S*-$PrNO_3$]‡ S99
R-$PrNO_3$-O…O_2…*S*-$PrNO_3$-O S100
References S101

List of Figures

Figure S1. Active space orbitals visualized along the reaction coordinate, MeO_2• + MeO_2• → MeO• + MeO• + $^3O_2$ as an example. Orbital occupation numbers are from MCSCF populations for natural orbitals of the optimized stationary points........ 9
Figure S2. ASCI results for HO_4H. 22
Figure S3. ASCI results for MeO_4H. 23
Figure S4. ASCI results for MeO_4Me........ 24
Figure S5. Reaction coordinate of the formation and decomposition of HO_4H. 25
Figure S6. Reaction coordinate of the formation and decomposition of MeO_4H. 26
Figure S7. Reaction coordinate of the formation and decomposition of MeO_4Me. 26
Figure S8. Reaction coordinate of the formation and decomposition of EtO_4Et. 27
Figure S9. Reaction coordinate of the formation and decomposition of $iPrO_4iPr$. 27
Figure S10. Reaction coordinate of the formation and decomposition of AcO_4Me. 28
Figure S11. Reaction coordinate of the formation and decomposition of AcO_4Ac. 28
Figure S12. Reaction coordinate of the formation and decomposition of $AllylO_4Allyl$. 29
Figure S13. Reaction coordinate of the formation and decomposition of $AceO_4Ace$. 29
Figure S14. Reaction coordinate of the formation and decomposition of $AceO_4$-S-BuOH. 30
Figure S15. Reaction coordinate of the formation and decomposition of *R*-BuOH-O_4-*R*-BuOH. 30
Figure S16. Reaction coordinate of the formation and decomposition of *R*-BuOH-O_4-*S*-BuOH. 31
Figure S17. Reaction coordinate of the formation and decomposition of *R*-$PrNO_3$-O_4-*R*-$PrNO_3$. 31
Figure S18. Reaction coordinate of the formation and decomposition of *R*-$PrNO_3$-O_4-*S*-$PrNO_3$. 32

S1. Global minimum HF-SCF solution for coupled-cluster calculations: Orbital rotation method

As stated in the main article, Hartree-Fock calculations do not necessarily automatically converge to the true global minimum state but can land into a local minimum instead. This issue was circumvented by applying a series of perturbations to the found HF orbitals in a way that allows exploring a wider range of potential solutions, following the procedure suggested by Vaucher and Reiher.[1] In practice, this was done by selecting 15 of the highest occupied orbitals and 15 of the lowest unoccupied orbitals and doing 10 random rotations between these orbitals (rotating an occupied orbital with an unoccupied orbital). The rotation for each orbital pair was performed by a random angle between 0 and 90 degrees. A set of 100 calculations with 10 pairs of random orbital rotations each was prepared and carried out at R(O)HF/cc-pVDZ-F12 level of theory.

S1.1. Orbital rotations for CCSD(T)-F12/cc-pVDZ-F12 calculations

All canonical coupled-cluster calculations were carried out with Molpro,[2] so the preceding Hartree-Fock calculations and orbital rotations were done with the same software. With Molpro, the used orbital rotation scheme (10 random mixing between orbital pairs, 100 calculations) did not consider whether the starting orbital set had 15 occupied orbitals, and sometimes led into rotations with negative orbital indices. These rotations were disregarded by Molpro, thus leading to calculations containing fewer than 10 rotations. The lowest energy wave function from the set of 100 calculations was chosen as the reference for CCSD(T)-F12/cc-pVDZ-F12 calculation.

S1.2. Orbital rotations for DLPNO-CCSD(T)-F12/cc-pVTZ-F12 calculations

All DLPNO-CCSD(T)-F12 calculations were done with ORCA-4.2.1 software,[3-4] and the Hartree-Fock orbital mixing was also performed in the ORCA calculations. The employed version of ORCA does not support DLPNO-CCSD(T)-F12 with ROHF reference wave functions, so all DLPNO-CCSD(T)-F12 calculations had to be done using UHF reference wavefunction (both closed-shell and open-shell structures, because their energies were compared to each other).

The orbital mixing were done using the R(O)HF/cc-pVDZ-F12 default orbitals as a starting point. With ORCA, the orbital mixing scheme was modified as follows: if the molecule had fewer than 15 occupied orbitals, the number of occupied orbitals to perform the mixing from was decreased to the number of occupied orbitals.

From the 100 calculations, the wave function with the lowest energy was then converted non-iteratively into a UHF wavefunction, which was further reoptimized in UHF/cc-pVTZ-F12 and then used as the reference wave function for DLPNO-CCSD(T)-F12/cc-pVTZ-F12 calculations.

S2. Generation of the CASSCF active spaces for the studied structures along the reaction coordinate

The DFT-optimized global minimum structures of the tetroxides from the conformer sampling were used as a starting point for the CASSCF calculations. All CASSCF calculations were done with the Firefly QC package,[5] which is partially based on the GAMESS-US source code.[6]

The starting geometries for the CASSCF calculations were prepared as follows: the DFT-optimized global minimum geometries of the tetroxide-intermediates were used as a starting point for tetroxide (RO_4R') calculations. The starting structures for decomposition transition states ($RO\cdots O_2\cdots OR'$) were generated by lengthening the outer O-O bonds of the O-O-O-O -moiety to 1.65 Å and shortening the inner O-O bond to 1.35 Å in the DFT-optimized RO_4R' structures. Initial geometries for the formation transition state (ROO···OOR') calculations were obtained by lengthening the inner O-O distances in the DFT-optimized global minimum geometries to 1.80-1.90 Å. Starting geometries for the minimum corresponding to peroxyl radical pair cluster (reactant complex; $RO_2\ldots R'O_2$) were prepared by lengthening the inner O-O distance even further to 2.5-3.0 Å. To simulate non-interacting RO_2• + $R'O_2$• pairs, the distance between the peroxyl radical oxygens was frozen to 15 Å. The other extreme of the reaction path, the $RO\ldots O_2\ldots R'O$ product complex minimum, was prepared from the structure resembling the decomposition transition state by lengthening the outer O-O bonds to 2.3 Å.

CASSCF active spaces were constructed using the initial structure of decomposition transition state. First, B3LYP/6-311++G(d,p) single-point calculations were conducted and the resulting orbitals were visualized with Chemcraft.[7] Orbitals corresponding to three O-O σ-orbitals, two inner O-O π-orbitals, and three O-O σ*-orbitals were selected as the active space for the CASSCF(10,8)/6-311++G(d,p) optimizations. CASSCF orbital optimization was done by reading in the B3LYP/6-311++G(d,p) MOs as a starting point and then listing the five active occupied orbitals as HOMO, HOMO-1, HOMO-2, HOMO-3, HOMO-4. Similarly, the three active unoccupied/virtual orbitals were listed as LUMO, LUMO+1, and LUMO+2. The CASSCF calculation was then carried out at the fixed molecular geometry, resulting in optimized orbitals. These orbitals were then read in as starting orbitals for all calculations to ensure that the active space remained the same for all the structures along the reaction coordinate. Active space orbitals are visualized for all stationary points along the reaction coordinate for the 2 MeO_2• → 2 MeO• + $^3O_2$ reaction (Figure S1). Active space orbitals for larger systems look similar.

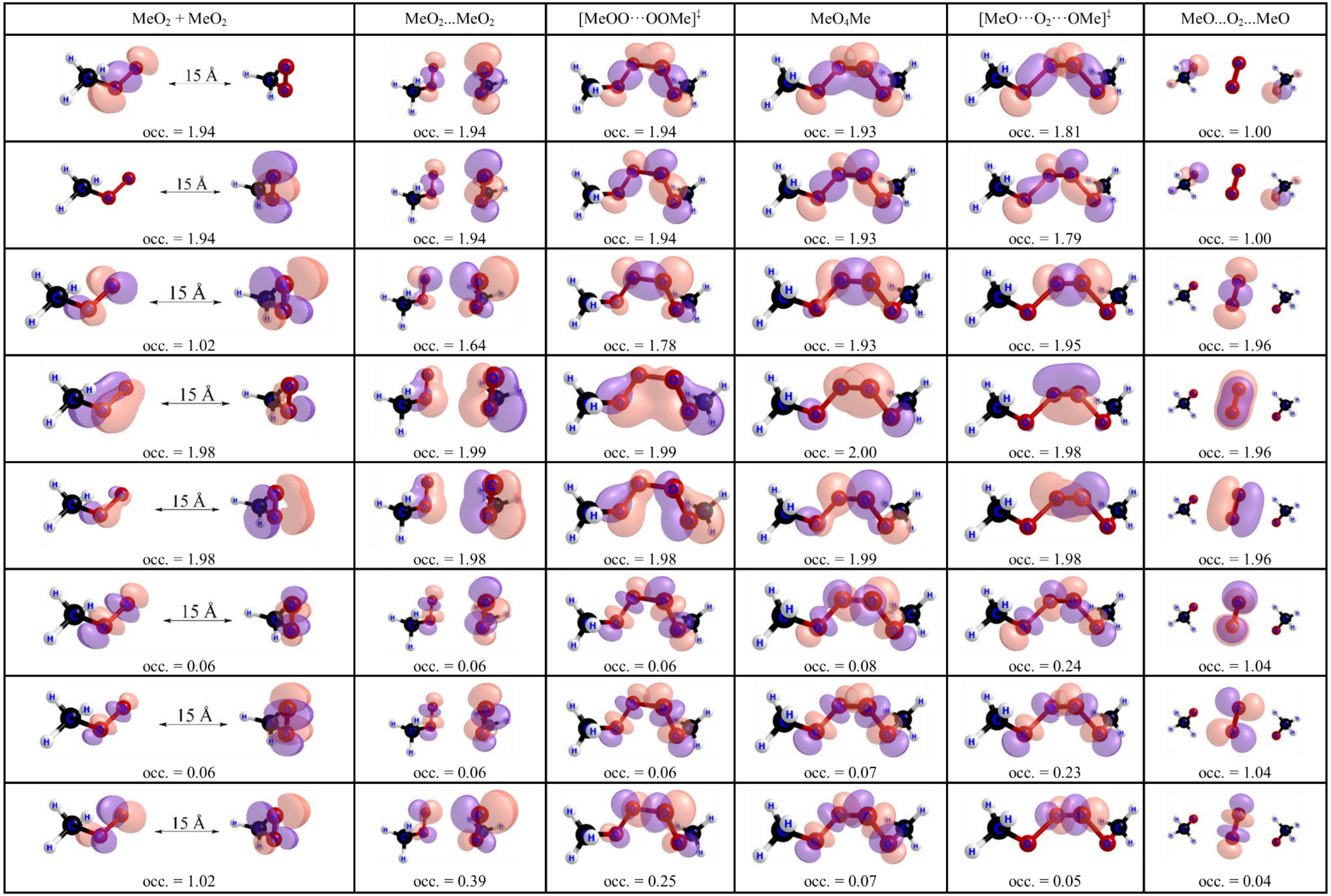

Figure S1. Active space orbitals visualized along the reaction coordinate, MeO_2• + MeO_2• → MeO• + MeO• + $^3O_2$ as an example. Orbital occupation numbers are from MCSCF populations for natural orbitals of the optimized stationary points.

S3. Size-consistency analysis of CASSCF and XMCQDPT2

To calculate vibrational frequencies for the various RO_2-structures, the CASSCF(10,8) active space was split in half, giving (5,4) active spaces for each investigated RO_2. To make sure that the electronic energy description remained size-consistent with the (10,8) active space, a comparison between 2×E(5,4) vs. E(10,8) was done with both CASSCF and XMCQDPT2 (Table S1). The RO_2 structures for this analysis were extracted from the 15 Å separated pairs of RO_2 + R'O_2 without re-optimization of geometries. Based on this analysis, CASSCF is size-consistent, while XMCQDPT2 is not size-consistent with the selected active space. (The latter result is a potential reason for why this type of active-space splitting, or indeed any reaction rate estimations, are seldom carried out in multireference studies of bimolecular reactions.) The residual differences in 2×E(5,4) vs. E(10,8) are most likely due to basis set superposition error (BSSE), as the 15 Å separation between the RO_2 monomers does not completely eliminate all superimposed electron density. This was further verified by comparing the 2×E(5,4) energy of the MeO_2 system to a 100 Å separated MeO_2 + MeO_2 pair (10,8): the ΔE_{el} dropped from 0.02 kcal/mol to -0.0001 kcal/mol. The same treatment was done with XMCQDPT2 as well, to make sure that the discrepancy is not simply due to a larger effect of BSSE on the XMCQDPT2 energies. XMCQDPT2 shows several kcal/mol size-consistency error across all the systems studied and increasing the separation for MeO_2 + MeO_2 from 15 Å to 100 Å only decreases the energy difference by 0.02 kcal/mol, further demonstrating that the XMCQDPT2 is not size-consistent. All CASSCF and XMCQDPT2 calculations were carried out with Firefly QC package.[5]

Table S1. Size-consistency analysis of CASSCF and XMCQDPT2.[1]

RO_2 + R'O_2	Difference[2] of 2×(5,4) vs. (10,8) in kcal mol^{-1} CASSCF	XMCQDPT2
HO_2 + HO_2	0.01	-2.96
MeO_2 + HO_2	0.02	-8.44
MeO_2 + MeO_2	-0.02	-4.97
EtO_2 + EtO_2	-0.03	-11.33
iPrO_2 + *i*PrO_2	-0.02	-9.15
AcO_2 + MeO_2	-0.04	-12.84
AcO_2 + AcO_2	-0.06	-13.10
$AceO_2$ + $AceO_2$	-0.01	-14.81
$AllylO_2$ + $AllylO_2$	-0.05	-15.00
$AceO_2$ + *S*-BuOH-O_2	-0.02	-11.78
R-BuOH-O_2 + *R*-BuOH-O_2	0.01	-12.77
R-BuOH-O_2 + *S*-BuOH-O_2	0.02	-12.72
R-$PrNO_3$-O_2 + *R*-$PrNO_3$-O_2	0.00	-16.99
R-$PrNO_3$-O_2 + *S*-$PrNO_3$-O_2	0.09	-16.91
Average	-0.01	-11.70
Standard Deviation	0.04	3.96

[1]Comparison is done between 15 Å separated RO_2 + R'O_2 pair with (10,8) active space and RO_2 with a corresponding (5,4) active space.
[2]Negative difference implies that 2×(5,4) is higher in energy than (10,8), and *vice versa*.

S4. Thermodynamic data for bimolecular rate coefficient calculations

Table S2. Gibbs energy changes, calculated with CASSCF/6-311++G(d,p).[1]

R	R’	Gibbs energy change, kcal/mol				
		RO_2+R’O_2[2]	RO_2…RO_2	[ROO···OOR']	RO_4R	[RO···O_2···OR']
H	H	0.00	4.98	**12.83** (11.65)	9.50	8.89
Me	H	0.00	5.93	**13.46** (12.27)	10.47	9.01
Me	Me	0.00	6.21	**13.79** (12.54)	13.13	11.90
Et	Et	0.00	5.69	**13.27** (12.05)	12.31	10.27
*i*Pr	*i*Pr	0.00	6.75	**14.78** (13.53)	14.36	14.07
Ac	Me	0.00	**6.25** (5.32)	-	5.20	4.18
Ac	Ac	0.00	**3.52** (2.71)	-	-0.60	-1.03
Allyl	Allyl	0.00	5.75	**13.89** (12.71)	11.89	9.82
Ace	Ace	0.00	6.26	**15.27** (13.98)	12.26	11.04
Ace	*S*-BuOH	0.00	3.09	**13.31** (11.93)	10.36	9.54
R-BuOH	*R*-BuOH	0.00	2.19	**15.90** (14.60)	9.87	9.12
R-BuOH	*S*-BuOH	0.00	1.21	**12.37** (11.03)	8.93	8.20
R-PrNO_3	*R*-PrNO_3	0.00	3.59	**12.86** (11.57)	9.76	9.30
R-PrNO_3	*S*-PrNO_3	0.00	4.91	**12.68** (11.28)	10.15	9.94

[1]Boldfaced values correspond to the highest energy point in the Gibbs energy surface for the reaction coordinate (=$\Delta G^‡$). Values in brackets correspond to vibrationally scaled Gibbs energy barrier heights. The scaling factor for the G-correction is 0.906 - the suggested value for HF/6-311+G(3df,2pd). Thermochemical corrections were calculated at 298.15 K and 1 atm.

[2]Gibbs energy is calculated from the sum of 2×CASSCF(5,4) E_{el}+G-correction, instead of CASSCF(10,8).

Table S3. Reaction enthalpy changes, calculated with CASSCF/6-311++G(d,p).[1]

R	R’	Reaction enthalpy change, kcal/mol				
		RO_2+R'O_2[2]	RO_2...RO_2	[ROO···OOR']	RO_4R	[RO···O_2···OR']
H	H	0.00	-2.77	1.47	-2.38	-2.03
Me	H	0.00	-2.77	1.69	-1.69	-2.09
Me	Me	0.00	-0.89	1.15	0.45	1.01
Et	Et	0.00	-1.68	0.96	0.09	-0.08
*i*Pr	*i*Pr	0.00	-1.18	2.10	1.78	3.14
Ac	Me	0.00	-2.98	-	-6.95	-7.12
Ac	Ac	0.00	-4.06	-	-12.65	-13.42
Allyl	Allyl	0.00	-1.18	1.88	-0.30	-0.85
Ace	Ace	0.00	-1.31	2.11	-0.70	-1.01
Ace	*S*-BuOH	0.00	-4.98	-0.78	-3.48	-3.50

R-BuOH	*R*-BuOH	0.00	-5.84	2.42	-3.56	-3.77
R-BuOH	*S*-BuOH	0.00	-5.92	-1.41	-4.36	-4.36
R-$PrNO_3$	*R*-$PrNO_3$	0.00	-5.18	-0.38	-3.45	-3.30
R-$PrNO_3$	*S*-$PrNO_3$	0.00	-5.14	-1.78	-3.85	-3.10

[1]Thermochemical corrections were calculated at 298.15 K and 1 atm.

[2]Enthalpy is calculated from the sum of 2×CASSCF(5,4) E_{el}+H-correction, instead of CASSCF(10,8).

S5. TST reaction rates based on CASSCF energetics

The bimolecular reaction rate coefficients for the RO_2• + $R'O_2$• reactions (involving both tetroxide formation and decomposition) were estimated using elementary transition state theory. The Gibbs energy of activation, $\Delta G^{\ddagger}$, was calculated from the Gibbs energy difference between the 2×CASSCF(5,4)/6-311++G(d,p) peroxyl radicals and the CASSCF(10,8)/6-311++G(d,p) formation transition states, $[ROO\cdots OR']^{\ddagger}$, which generally were the highest energy points in the Gibbs energy surface of the reactions. For AcO_4Me and AcO_4Ac, the formation transition state does not exist, and the highest energy point in the Gibbs energy surface used in the rate calculations corresponded instead to the reactant complex, $RO_2\ldots R'O_2$. The reaction rate coefficients were calculated with the following equation:

$$k(T) = \sigma \frac{k_b T}{h} e^{-\frac{\Delta G^{\ddagger}}{RT}} \left(\frac{RT}{p_0 N_A}\right) \times 10^6$$

where the $\left(\frac{RT}{p_0 N_A}\right)$ term is the ideal gas volume of one molecule, the factor 10^6 scales the volume element to cm^3 $molecule^{-1}$, and σ is the reaction path symmetry number. σ=2 for all reactions where the peroxyl radicals are identical, and the corresponding formation transition states $[ROO\cdots OR']^{\ddagger}$ are C_1-symmetric. For all other reactions σ=1. Tunneling was not considered as the studied reaction pathway only involves bond formation and bond breaking between heavy atoms. Rate coefficients were calculated using 298.15 K reference temperature, which was also used in the calculation of the Gibbs energy correction terms in vibrational analyses. Gibbs energy changes and reaction enthalpies can be found in the previous section (S4). The calculated reaction rate coefficients and their comparison to experimental rate coefficients[8] are shown in Table S4.

Table S4. Bimolecular reaction rate coefficients, calculated from CASSCF Gibbs energy changes.

RO_4R' in RO_2• + $R'O_2$• → products	k(298.15 K) CASSCF cm^3 $molecule^{-1}$ s^{-1}	k(298.15 K) CASSCF cm^3 $molecule^{-1}$ s^{-1} G-correction scaled by 0.906	k(298.15 K) Experimental[1] cm^3 $molecule^{-1}$ s^{-1}
MeO_4Me	2.0×10^{-17}	1.6×10^{-16}	3.5×10^{-13}
EtO_4Et	4.7×10^{-17}	3.7×10^{-16}	6.4×10^{-14}
$iPrO_4iPr$	3.7×10^{-18}	3.0×10^{-17}	1.0×10^{-15}
AcO_4Me	6.7×10^{-12}	3.2×10^{-11}	1.1×10^{-11}
AcO_4Ac	6.6×10^{-10}	2.6×10^{-9}	1.6×10^{-11}
$AceO_4Ace$	3.2×10^{-18}	2.8×10^{-17}	2.1×10^{-12}
$AllylO_4Allyl$	3.4×10^{-17}	2.4×10^{-16}	-
$AceO_4BuOH$	4.4×10^{-17}	4.5×10^{-16}	-
R,R-BuOH-O_4-BuOH	1.1×10^{-18}	1.0×10^{-17}	-
R,S-BuOH-O_4-BuOH	2.2×10^{-16}	2.1×10^{-15}	-
R,R-$PrNO_3$-O_4-$PrNO_3$	1.9×10^{-16}	1.7×10^{-15}	-
R,S-$PrNO_3$-O_4-$PrNO_3$	1.3×10^{-16}	1.4×10^{-15}	-

[1] Experimental rate coefficients are from ref. 8

The rate coefficients calculated based on CASSCF data are internally consistent for the most part, as simple primary alkyl peroxyl radicals, MeO_2• and EtO_2• have similar rate coefficients, while the secondary peroxyl radical *i*PrO_2• has a smaller rate coefficient, as predicted based on experimental data. The calculated rate coefficients for acyl peroxyl radical reactions are also consistent with the experimental results, in that these are a few orders of magnitude faster than other peroxyl radical reactions. However, the calculated reaction rate coefficient for acetonyl peroxyl self-reaction is distinctly different from the experimental rate coefficient. This is probably due to the lack of dispersion in CASSCF, while the $[AceOO\cdots OOAce]^{\ddagger}$ structure shows clear indications of intermolecular H-bonding. These weak interactions might actually be stronger, stabilize the transition states, and lower the barriers along the reaction path.

The studied peroxyl radicals with hydroxyl groups and nitrate groups do not have direct analogues for which experimental rate data are available, but experimental results for peroxyl radicals with hydroxyl groups suggest that the inclusion of hydroxyl groups should enhance the reactivity in comparison to equivalent alkyl peroxyl radicals. This trend can also be observed in the calculated rate coefficients, apart from the *R,R*-BuOH-O_4-BuOH, which in any case has a peculiar formation barrier in comparison to *R,S*-BuOH-O_4-BuOH. The rate coefficients for OH-systems should probably be even larger, but the discrepancy between calculated rates and experimental rates are presumably due to ill-described dynamical electron correlation effects in CASSCF when using small active spaces.

S6. Master equation modeling of selected reaction rates based on CCSD(T) and DFT energetics

As discussed in the previous section, the CASSCF energies are not accurate enough to reproduce the experimentally observed kinetics for the studied reaction. On the other hand, CASSCF, along with XMC-QDPT2 and ASCI-SCF(PT2) results strongly suggest that the rate-limiting step in the total reaction is either the formation of the tetroxide, or the formation of the pre-reactive complex (for reactions with barrierless tetroxide formation). By neglecting the decomposition TS of the tetroxide (which requires multiconfigurational treatment), the total reaction rate can be estimated using energies calculated with single-reference methods (Scheme S1).

To that end, we optimized the geometries and calculated the frequencies of the pre-reactive $RO_2...R'O_2$ complexes and the formation transition state structures $[ROO\cdots OOR']^{\ddagger}$ with ωB97X-D/aug-cc-pVTZ. We also tried to calculate CCSD(T)-F12/cc-pVDZ-F12 corrections to the total energy for these stationary points but were not able to get the underlying Hartree-Fock wavefunction to properly converge to the expected open-shell singlet wavefunction for $RO_2\ldots R'O_2$. Thus, the coupled-cluster energy corrections are limited to the energy difference of the separate peroxyl radicals and tetroxide, as well as the energy difference of the two alkoxyl radicals + O_2 and the tetroxide. The zero-point energy corrected total energies (E+ZPE) for kinetics simulations were calculated by adding the ZPE at ωB97X-D/aug-cc-pVTZ to the total energies at CCSD(T)-F12/cc-pVDZ-F12 or ωB97X-D/aug-cc-pVTZ. Vibrational frequencies and rotational constants were calculated at ωB97X-D/aug-cc-pVTZ.

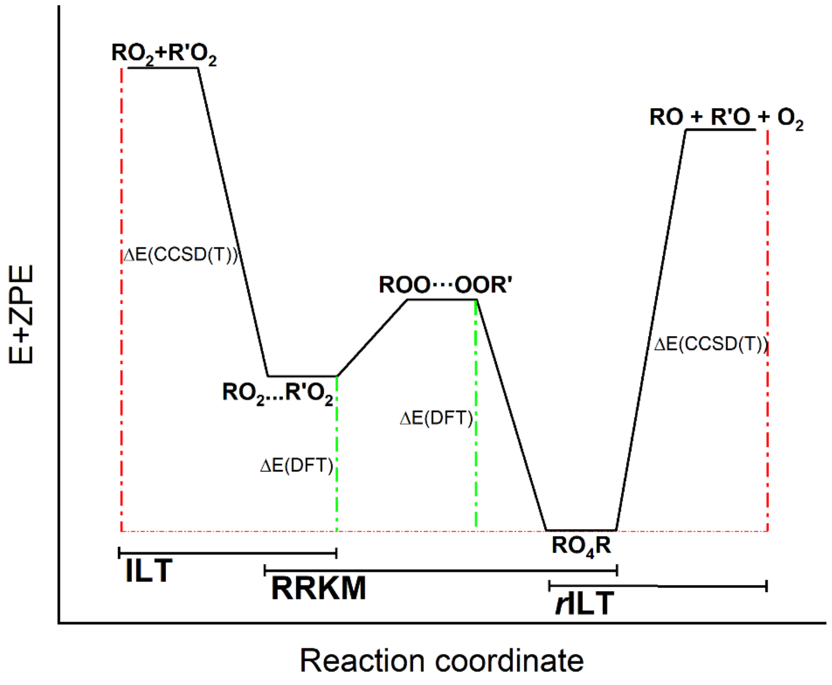

Scheme S1. Schematic of the potential energy surface, and the used methods in MESMER simulations.

We used the MESMER[9] program (Master Equation Solver for Multi-Energy Well Reactions) for simulating the kinetics of the tetroxide formation (and decomposition) reaction (Scheme S1). The total reaction (RO_2 + $R'O_2$ → RO + R'O + O_2) was simulated with a model consisting of three consecutive steps:

1) The barrierless association reaction of two peroxyl radicals into pre-reactive complex (RO_2 + RO_2 → RO_2…$R'O_2$) with ILT (Inverse Laplace Transform) method

2) Unimolecular isomerization of the pre-reactive complex into tetroxide via the formation transition state (RO_2…$R'O_2$ → $[ROO\cdots OOR']^{\ddagger}$ → RO_4R') with RRKM (Rice-Ramsperger-Kassel-Marcus) theory

3) Irreversible decomposition of the tetroxide into alkoxyl radicals and molecular oxygen (RO_4R' → RO + R'O + O_2) with reverse-ILT method (*r*ILT).

The MESMER simulations were only conducted for reactions for which canonical coupled-cluster corrections and experimental data were available: MeO_4Me, EtO_4Et, $iPrO_4iPr$, $AceO_4Ace$, AcO_4Ac, and AcO_4Me.

S6.1. General simulation parameters

In a MESMER simulation, the intermediate minimum structures of the reaction are treated with a type 'Modelled' (Table S5). This means, that these species are allowed to relax energy *via* collisions with a bath gas (N_2). A Lennard-Jones potential is used to calculate the collision frequency of the 'Modelled' compounds with the bath. The Lennard-Jones potential depends on the ε and σ parameters. These parameters were estimated with thermodynamic critical points,[10] which in turn were calculated using the Joback group contribution method.[11] Identical ε and σ parameters were used for both RO_2…$R'O_2$ and RO_4R' structures. We used the 'Exponential Down' collisional energy transfer model, with an average energy transfer in a deactivating collision, $\langle \Delta E \rangle_d$=100 cm^{-1} in all simulations.

Other constant parameters across all simulations were the 'grainSize' of 50 cm^{-1}, 'energyAboveTheTopHill' of 20 kT, Arrhenius parameters of E_a = 0.0 kcal mol^{-1} and A = 1.0×10^{-10} cm^3 s^{-1} $molecule^{-1}$ for the *r*ILT for simulating the dissociation of tetroxide into product sink. All simulations were run at 298.15 K and 760.0 Torr.

Table S5. Type definitions of the stationary points in MESMER simulations.

Molecule(s)	Role
RO_2	Deficient reactant
$R'O_2$	Excess reactant
RO_2…$R'O_2$	Modelled
$[ROO\cdots OOR']^{\ddagger}$	Transition state
RO_4R'	Modelled
RO + R'O (+ O_2)	Sink

S6.2. Barrierless association (**ILT**)

The association reaction of the peroxyl radicals into pre-reactive complex (RO_2 + $R'O_2$ → RO_2…$R'O_2$) was simulated with the ILT-method. As MESMER does not support true second-order

association reactions (A + A → B), we simulated the reaction using pseudo-first order conditions ($[RO_2] << [R'O_2]$) for all studied systems. We used excess reactant concentration of 5.0×10^{12} molecule cm^{-3}, which is of a similar order of magnitude as initial concentrations of peroxyl radicals in reported experiments. Apparently the association of two identical molecules can be simulated with this approximation as the reactant molecules (even when identical) can be treated as two different compounds in MESMER simulation. Thus, the simulation will resolve the pseudo-first order reaction profile even for A + A → B type of reaction. For AcO_4Ac and AcO_4Me systems, the ILT-method was used to estimate the rate of tetroxide formation instead of the formation of a pre-reactive complex as the tetroxide formation is barrierless for these systems.

The ILT-method requires Arrhenius activation energy and pre-exponential factor of the reaction as input parameters. We derived these parameters from reaction rate coefficients that we calculated using long-range transition-state theory (LR-TST).[12] With LR-TST, we assume the association reaction to occur between two dipoles (two peroxyl radicals) and therefore used the following equation to calculate the rate coefficients:

$$k(T) = C_1 \mu^{-\frac{1}{2}} (d_1 d_2)^{\frac{2}{3}} T^{-\frac{1}{6}}$$

in which C_1=5.42 is an empirical parameter, which in the studied reaction corresponds to two non-linear dipoles, μ is the reduced mass of the reacting system, d_1 and d_2 are the dipole moments of the two peroxyl radicals, and T is temperature. In the above equation, all the variables are in their respective atomic units. The bimolecular rate coefficients were then converted from the atomic units ($bohr^3$ $s(a.u.)^{-1}$ $molecule^{-1}$) to cm^3 s^{-1} $molecule^{-1}$ with conversion factor of 6.13×10^{-9}. The rate coefficients were calculated at two temperatures (298.15 K and 299 K) to solve the Arrhenius activation energy for the ILT-method.

$$E_a = R \times \frac{\ln k_1 - \ln k_2}{\frac{1}{T_1} - \frac{1}{T_2}}$$

Then, the pre-exponential factor of the Arrhenius equation was calculated with k(298.15) by

$$A = e^{\ln k(298.15) + \frac{E_a}{RT}}$$

The derivation of Arrhenius parameters from LR-TST rate coefficients yields constant negative activation energy for all systems (-0.098 kcal/mol, this value changes depending on which two temperatures are used for calculating the E_a). In the ILT, E_a was set to 0.00 kcal/mol as negative values were not accepted. The pre-exponential factor (which is equal to k(T) when E_a=0) varied from 9.6×10^{-11} cm^3 s^{-1} $molecule^{-1}$ to 4.3×10^{-10} cm^3 s^{-1} $molecule^{-1}$ across the studied systems. The LR-TST has limited applicability outside the low-temperature regime of kinetics, but the calculated association rate coefficients are likely to be of the correct order of magnitude. More rigorous derivation of these rate coefficients would require VTST (Variational Transition State Theory) calculations.

S6.3. Unimolecular isomerization (**RRKM**)

The tetroxide formation from the pre-reactive complex *via* the formation transition state was simulated using the standard RRKM theory with Eckart tunneling corrections. The related parameters were discussed above in 'General simulation parameters'.

S6.4. Irreversible dissociation (***r*ILT**)

The dissociation of the tetroxide into two alkoxyl radicals and O_2 was estimated with the *r*ILT method. In this estimation, the decomposition barrier is totally neglected. Additionally, MESMER does not support dissociation to three molecules so the decomposition was assumed to only produce two alkoxyl radicals. To take the energy of the O_2 into account, we added half of the energy of O_2 for both alkoxyl radicals to get correct potential energy surface.

S.6.5. Simulated reaction profiles (**ILT+RRKM+*r*ILT**)

The simulated reaction profiles were treated according to the logarithmic transformation of the pseudo-first order reaction rate law

$$\ln\left(\frac{[RO_2]}{[RO_2]_0}\right) = -k't$$

in which k' is the pseudo-first order rate coefficient. Plotting the above equation from the beginning of the simulation until the total consumption of the deficient reactant showed linear trend across all systems (all reactions demonstrated pseudo-first order kinetics). The bimolecular reaction rate coefficient (with respect to the consumption of RO_2) was then retrieved by dividing the slope of the linear fit (k') with the concentration of the excess reactant (5.0×10^{12} molecule cm^{-3}). The bimolecular reaction rate coefficients at 298.15 K, 760 Torr are shown in Table S6.

Table S6. Bimolecular reaction rate coefficients from MESMER simulations.

Reaction<br>$RO_2 + R'O_2 \rightarrow RO_4R' \rightarrow$ products	k(298.15) MESMER<br>$cm^3\ s^{-1}$ molecule^{-1}	k(298.15) Experimental[1]<br>$cm^3\ s^{-1}$ molecule^{-1}
2 $MeO_2 \rightarrow$ products	1.79×10^{-14}	3.50×10^{-13}
2 $EtO_2 \rightarrow$ products	1.72×10^{-15}	7.60×10^{-14}
2 $iPrO_2 \rightarrow$ products	1.76×10^{-16}	1.00×10^{-15}
2 $AceO_2 \rightarrow$ products	6.66×10^{-13}	8.00×10^{-12}
2 $AcO_2 \rightarrow$ products	3.38×10^{-10}	1.60×10^{-11}
$AcO_2 + MeO_2 \rightarrow$ products	3.27×10^{-10}	1.10×10^{-11}

[1] Experimental rate coefficients are from ref. 8

The calculated rate coefficients from MESMER-simulations agree with the experimental consumption rates of RO_2 surprisingly well. The calculated coefficients for reactions having the formation transition state are consistently one order of magnitude smaller than the experimental rate coefficients. The one order of magnitude difference may be due to the lack of coupled-cluster energy corrections for the barrier height of formation. Also, unlike the canonical TST rate coefficients calculated from CASSCF potential energy surface, these rate coefficients are internally completely consistent with experimentally observed reactivity trend: k($iPrO_4iPr$) < k(EtO_4Et) < k(MeO_4Me) < k($AceO_4Ace$) < k(AcO4Me) < k(AcO4Ac).

For AcO_4Ac and AcO_4Me, for which the formation reaction is barrierless, the rate coefficients are one order of magnitude larger than experimental rate coefficients. This difference is likely to originate from the shortcomings of the LR-TST and ILT approach. We demonstrated in the CASSCF calculations that the pre-reactive complexes exist also for these systems (as inflection points of PES

instead of minima), but in the MESMER simulations, these structures were neglected, because we did not have any rate estimations for the barrierless formation of tetroxides from pre-reactive complexes. Estimation of rates for barrierless, unimolecular reactions would require VTST calculations which we chose not to conduct for this study.

S7. ASCI-SCF(PT2) results

The ASCI-SCF(PT2) single-point calculations were done upon the CASSCF(10,8)/6-311++G(d,p) optimized geometries for all the stationary points along the reaction coordinate. For computational reasons, these calculations were only carried out for the three smallest systems - HO_4H, MeO_4H, and MeO_4Me - with full valence active spaces being (26,18), (32,24), and (38,30), respectively. Results with three basis sets, cc-pVDZ, cc-pVTZ, and 6-311++G(d,p), are shown in Tables S5-S7, and further visualized in Figures S1-S3.

The ASCI-SCF results show, similarly to XMC-QDPT2(10,8) single-point energies, that directly comparable results to CASSCF(10,8) would most likely require geometry optimization with the applied ASCI-SCF/ASCI-SCF(PT2) level of theory. In any case, the ASCI-SCF results do not predict any insurmountable barriers in comparison to the excess energy of the peroxyl radicals. The results also show a lot of variation between basis sets. Surprisingly, ASCI-SCF(PT2)/cc-pVDZ energies are closer to CASSCF(10,8)/6-311++G(d,p) relative energies than ASCI-SCF(PT2)/6-311++G(d,p) energies are. Unfortunately, the overall reaction energies (i.e. the energy of RO+R'O+O_2 minus the energy of RO_2+R'O_2) obtained for MeO_4Me using ASCI-SCF and any basis set or number of determinants is (when combined with Gibbs energy corrections e.g. from Table 3 in the main manuscript) incompatible with experimental results (as well as e.g. W1 calculations) indicating substantial formation of CH_3O + CH_3O products. This is very probably not due to any inherent failure of the ASCI method, but due to the errors incurred in the use of CASSCF geometries, especially the fixed-separation reactant geometry. For this reason, the ASCI results are not discussed extensively in the main manuscript.

The full valence electron determinant space of ASCI-SCF appears to be very accurate for the present full valence electron determinant spaces, as the PT2-corrected electronic energies are very similar to the variational energies. The PT2 contribution and the relative energy difference between 100k and 5M determinant energies increase with increasing system size. This is expected, as the truncated determinant spaces describe less of the total full valence determinant space for larger valence active spaces. Full valence spaces for HO_4H, MeO_4H, and MeO_4Me contain 7.3×10^7, 5.4×10^{11}, and 3.0×10^{15} determinants respectively. ASCI-SCF(PT2) calculations were carried out with Q-Chem software.[13]

Table S7. ASCI-SCF(PT2)/cc-pVDZ relative electronic energies in kcal mol^{-1} for HO_4H, MeO_4H, and MeO_4Me.

cc-pVDZ	RO_2+R'O_2	RO_2…R'O_2	ROO···OOR'	RO_4R'	RO···O_2···OR'	RO+R'O+O_2
**R=R'=H**						
**100k determinants**						
E(ASCI-SCF)	6.42	1.79	4.57	0.00	5.83	3.34
E(ASCI-SCF(PT2))	6.58	1.96	4.64	0.00	5.93	3.52

**5M determinants**						
E(ASCI-SCF)	6.61	1.98	4.65	0.00	5.94	3.52
E(ASCI-SCF(PT2))	6.61	1.98	4.65	0.00	5.95	3.52
**R=Me, R'=H**						
**100k determinants**						
E(ASCI-SCF)	6.60	1.24	4.45	0.00	4.68	21.69
E(ASCI-SCF(PT2))	6.84	1.30	4.49	0.00	4.44	21.91
**5M determinants**						
E(ASCI-SCF)	6.83	1.29	4.48	0.00	4.42	0.10
E(ASCI-SCF(PT2))	6.85	1.29	4.49	0.00	4.41	0.07
**R=Me, R'=Me**						
**100k determinants**						
E(ASCI-SCF)	3.65	4.67	2.71	0.00	7.09	19.68
E(ASCI-SCF(PT2))	6.03	4.58	2.46	0.00	5.81	22.65
**5M determinants**						
E(ASCI-SCF)	6.17	4.39	2.41	0.00	5.76	22.73
E(ASCI-SCF(PT2))	6.63	4.39	2.35	0.00	5.51	23.27

Table S8. ASCI-SCF(PT2)/cc-pVTZ relative electronic energies in kcal mol^{-1} for HO_4H, MeO_4H, and MeO_4Me

**cc-pVTZ**	RO_2+R'O_2	RO_2…R'O_2	ROO···OOR'	RO_4R'	RO···O_2···OR'	RO+R'O+O_2
**R=R'=H**						
**100k determinants**						
E(ASCI-SCF)	8.45	5.03	7.80	0.00	8.19	7.69
E(ASCI-SCF(PT2))	8.51	5.09	7.73	0.00	8.22	7.77
**5M determinants**						
E(ASCI-SCF)	8.51	5.09	7.72	0.00	8.22	7.75
E(ASCI-SCF(PT2))	8.41	5.09	7.72	0.00	8.22	7.75
**R=Me, R'=H**						
**100k determinants**						
E(ASCI-SCF)	27.32	23.06	26.37	0.00	25.71	41.88
E(ASCI-SCF(PT2))	27.67	23.38	26.63	0.00	25.87	42.48
**5M determinants**						
E(ASCI-SCF)	27.62	23.34	26.60	0.00	25.83	30.60
E(ASCI-SCF(PT2))	27.64	23.36	26.62	0.00	25.84	30.63
**R=Me, R'=Me**						
**100k determinants**						
E(ASCI-SCF)	24.04	26.59	6.60	0.00	15.38	42.91
E(ASCI-SCF(PT2))	26.79	26.34	6.32	0.00	14.20	44.87
**5M determinants**						
E(ASCI-SCF)	26.71	25.93	6.30	0.00	14.31	44.43
E(ASCI-SCF(PT2))	27.15	25.82	6.25	0.00	14.14	44.78

Table S9. ASCI-SCF(PT2)/6-311++G(d,p) relative electronic energies in kcal mol-1 for HO_4H, MeO_4H, and MeO_4Me.[1]

6-311++G(d,p)	RO_2+R'O_2	RO_2…R'O_2	ROO···OOR'	RO_4R'	RO···O_2···OR'	RO+R'O+O_2
**R=R'=H**						
**100k determinants**						
E(ASCI-SCF)	9.17	8.96	7.43	0.00	9.12	3.21
E(ASCI-SCF(PT2))	9.25	9.04	7.42	0.00	9.18	231.70[2]
**5M determinants**						
E(ASCI-SCF)	9.25	9.04	7.42	0.00	9.18	2.35
E(ASCI-SCF(PT2))	9.25	9.04	7.42	0.00	9.18	-85.28[2]
**E(CASSCF)**	3.92	-0.32	4.19	0.00	2.34	-12.46
**E(XMC-QDPT2)**	8.42	2.95	4.12	0.00	1.71	9.57
**R=Me, R'=H**						
**100k determinants**						
E(ASCI-SCF)	16.81	1.87	21.29	0.00	2.47	7.25
E(ASCI-SCF(PT2))	17.09	2.15	21.27	0.00	2.49	7.96
**5M determinants**						
E(ASCI-SCF)	17.06	2.14	21.26	0.00	2.49	7.92
E(ASCI-SCF(PT2))	17.08	2.16	21.27	0.00	2.49	7.95
**E(CASSCF)**	3.09	-1.23	3.87	0.00	1.21	-16.63
**E(XMC-QDPT2)**	10.88	4.06	4.65	0.00	1.03	12.62
**R=Me, R'=Me**						
**100k determinants**						
E(ASCI-SCF)	5.53	-8.08	-9.46	0.00	-1.00	-0.58
E(ASCI-SCF(PT2))	8.38	-8.86	-10.39	0.00	-2.34	3.61
**5M determinants**						
E(ASCI-SCF)	8.32	-9.08	-10.28	0.00	-2.10	32.37
E(ASCI-SCF(PT2))	8.86	-9.09	-10.38	0.00	-2.23	33.02
**E(CASSCF)**	0.88	-1.27	1.40	0.00	2.31	-17.41
**E(XMC-QDPT2)**	3.72	-0.56	-3.00	0.00	-3.42	3.98

[1]CASSCF(10,8) and XMC-QDPT2(10,8) results with same basis set are shown for comparison.

[2]Convergence issues, results are not reliable.

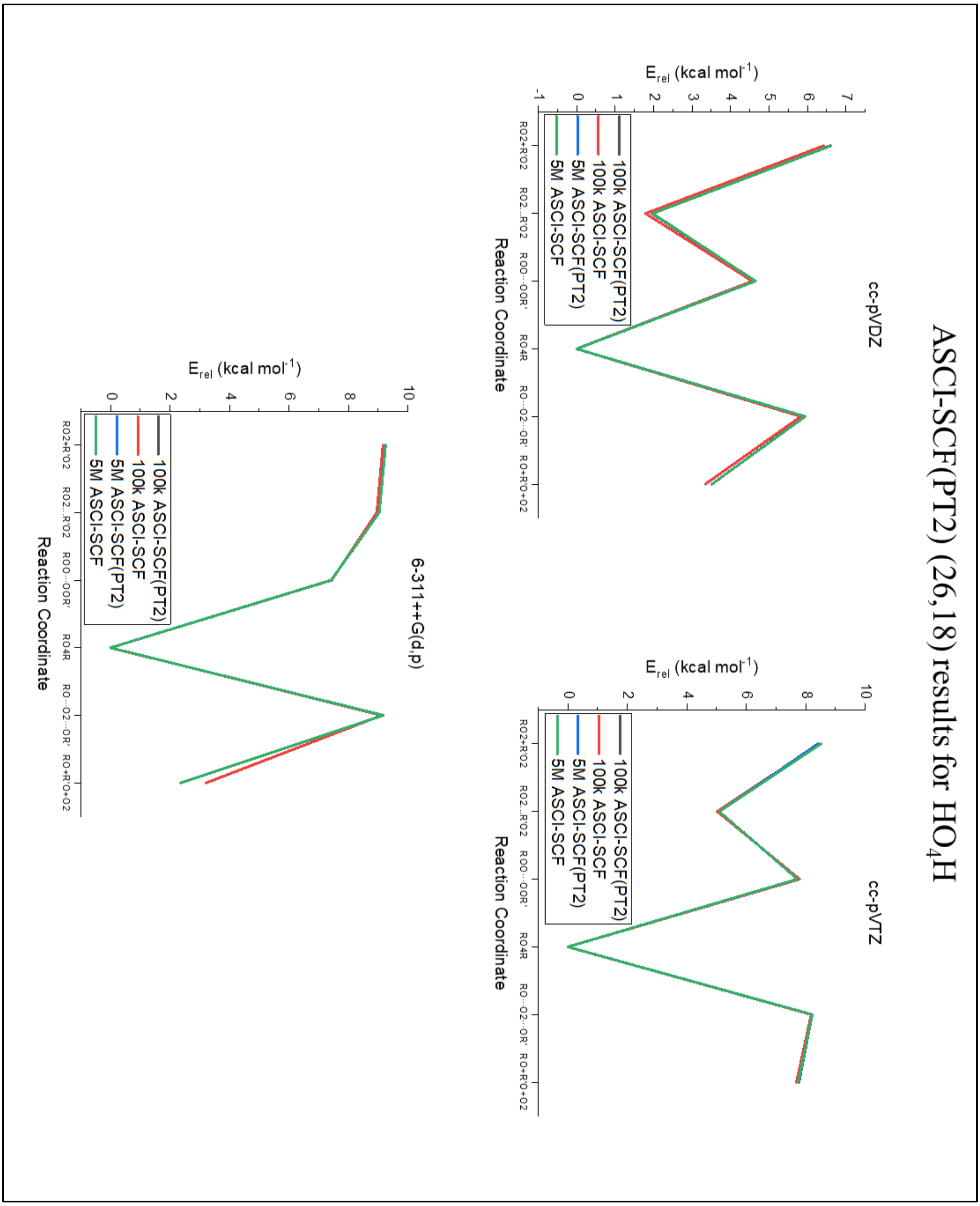

Figure S2. ASCI results for HO_4H.

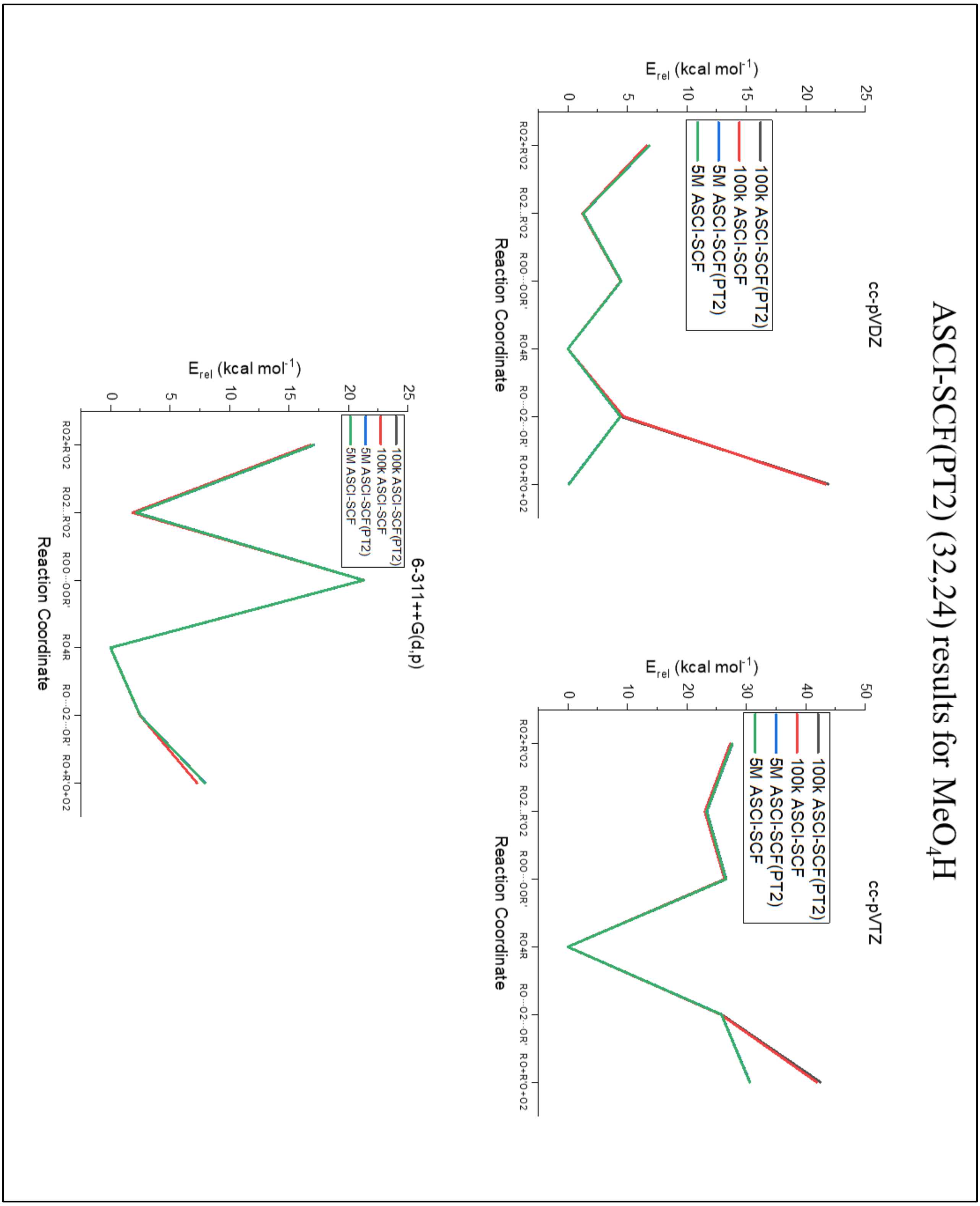

Figure S3. ASCI results for MeO_4H.

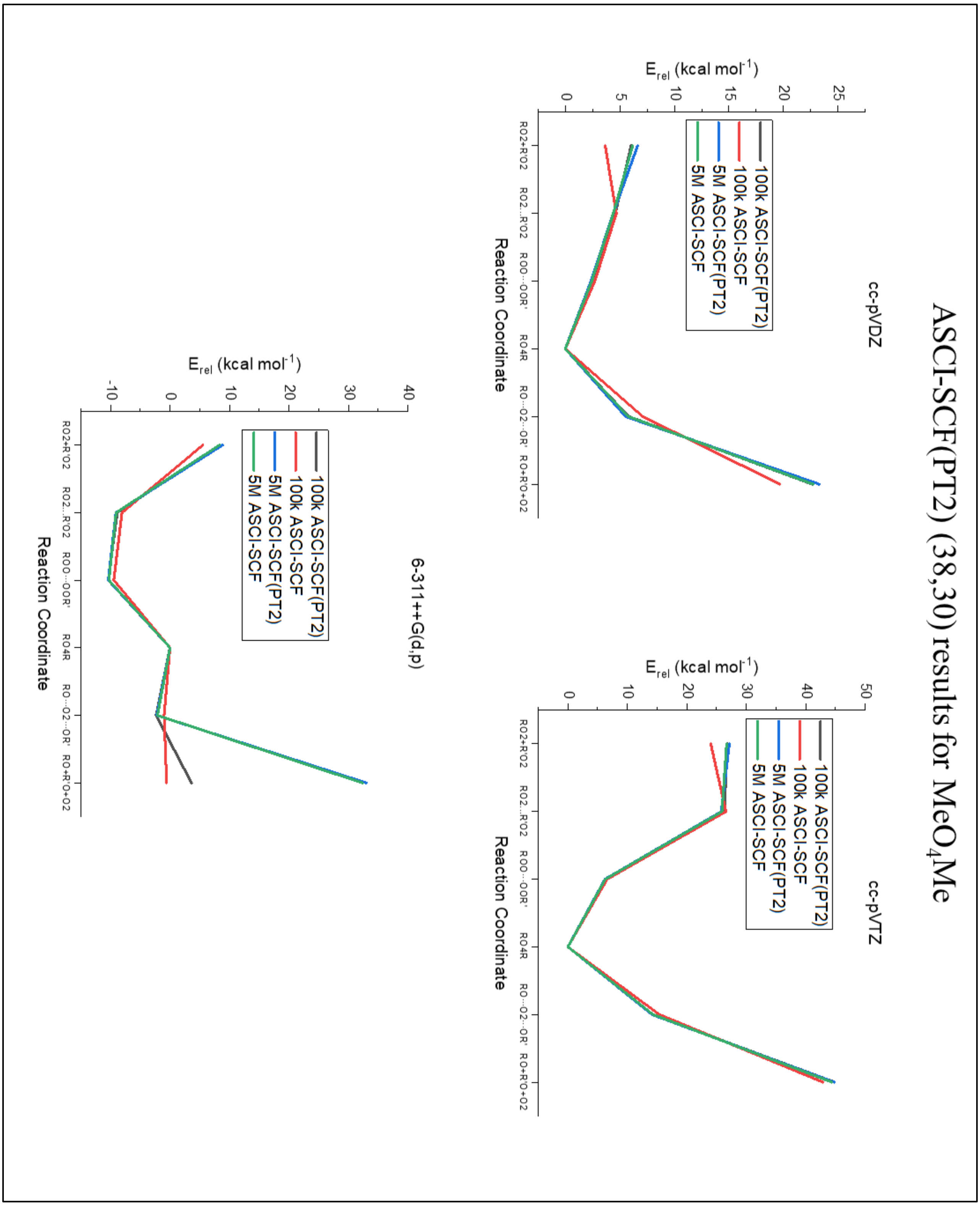

Figure S4. ASCI results for MeO_4Me.

S8. Reaction coordinate graphs

This section shows a graphical representation of all the calculated reaction coordinates. In the graphs, the gray line connecting the various stationary points correspond to CASSCF(10,8)/6-311++G(d,p) optimized reaction coordinate with respect to the electronic energy. The disconnected red values correspond to XMC-QDPT2(10,8)/6-311++G(d,p) single-point energies calculated using the CASSCF stationary points. The blue values correspond to coupled-cluster energies calculated using ωB97X-D/aug-cc-pVTZ optimized geometries. These energies are only calculated for points which can be reliably described with single-reference methods (DFT and coupled-cluster). All the energies are shown relative to the tetroxide intermediate, which is chosen as a zero-point for all different levels of calculations.

In addition, above the legend of the graph, the Gibbs energy change for the total reaction RO_2• + $R'O_2$• → RO• + R'O• + $^3O_2$ calculated with the highest applicable level-of-theory is shown.

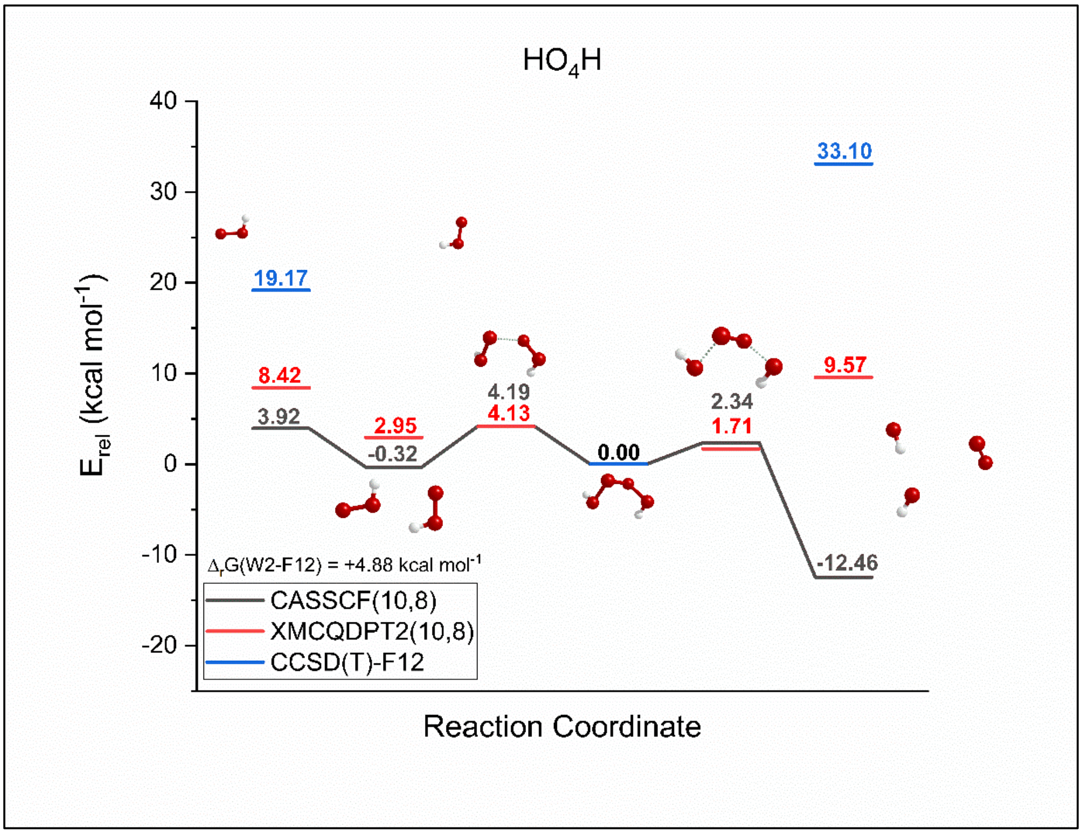

Figure S5. Reaction coordinate of the formation and decomposition of HO_4H.

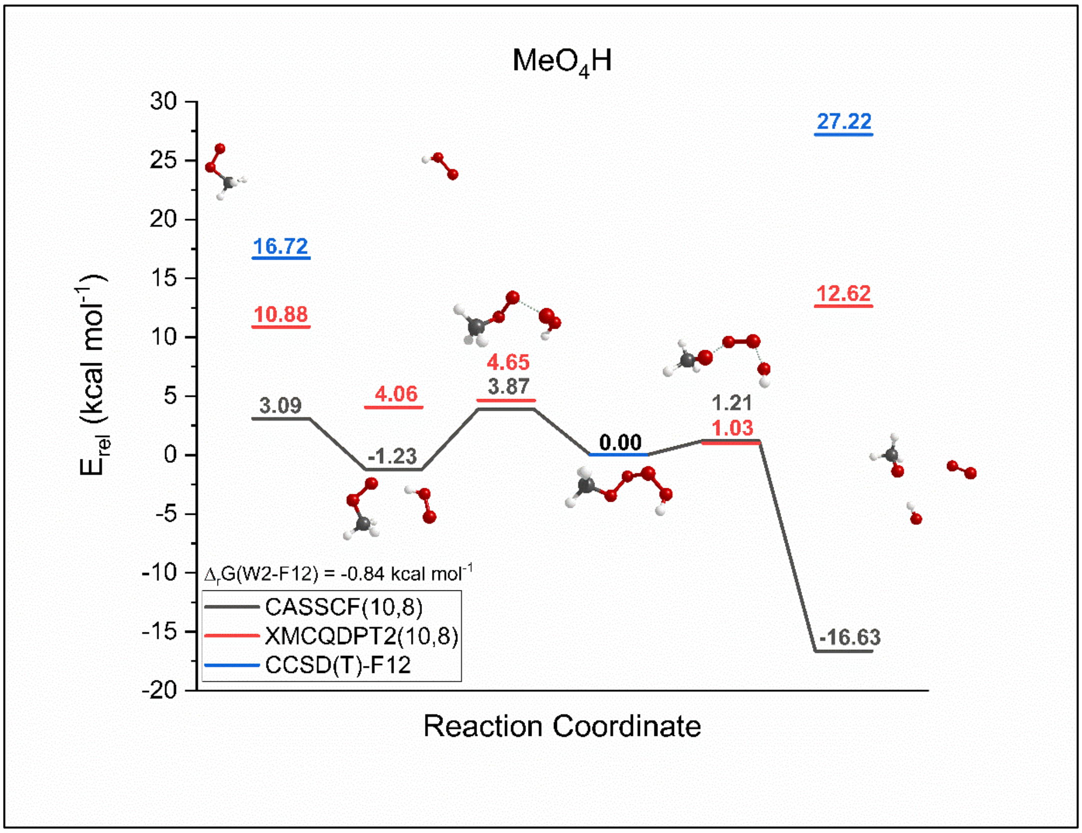

Figure S6. Reaction coordinate of the formation and decomposition of MeO_4H.

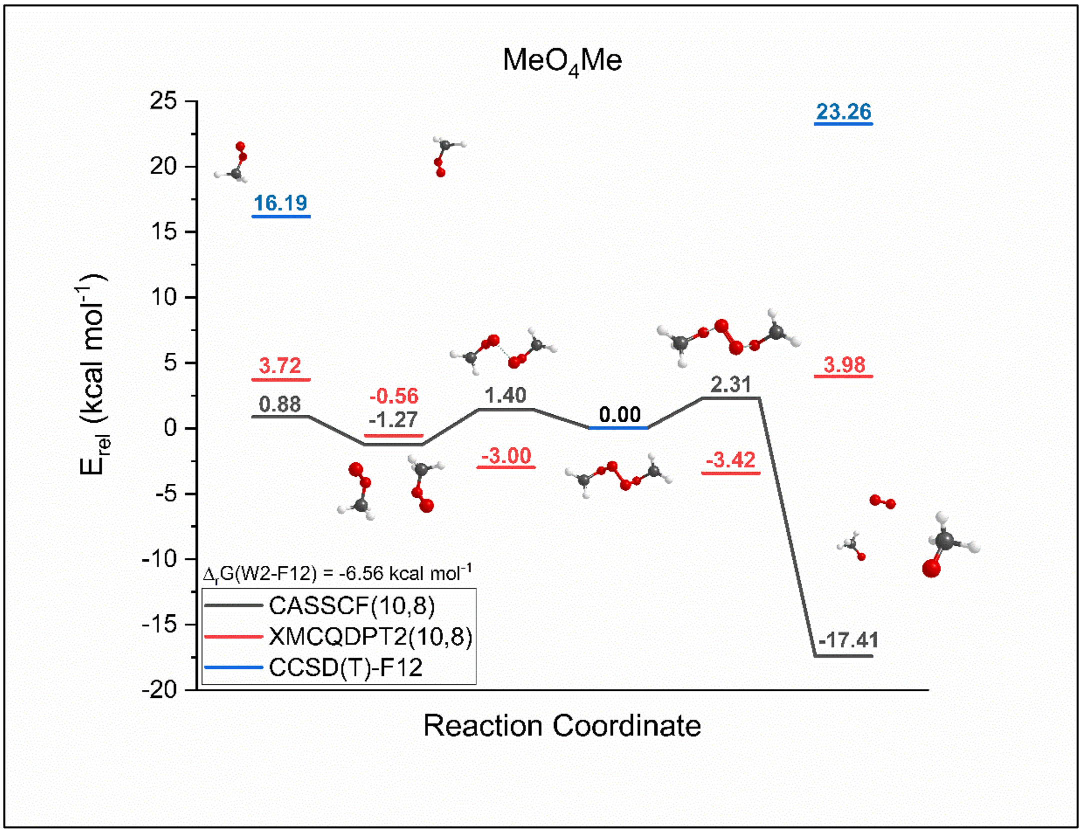

Figure S7. Reaction coordinate of the formation and decomposition of MeO_4Me.

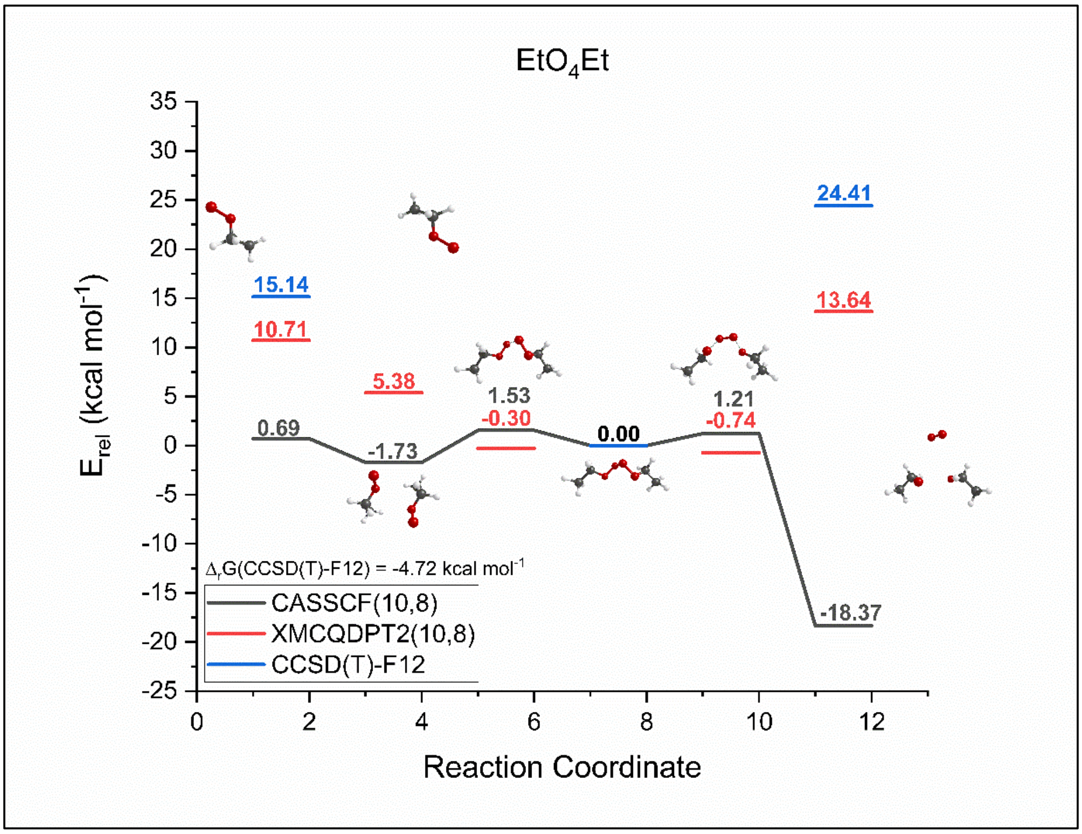

Figure S8. Reaction coordinate of the formation and decomposition of EtO_4Et.

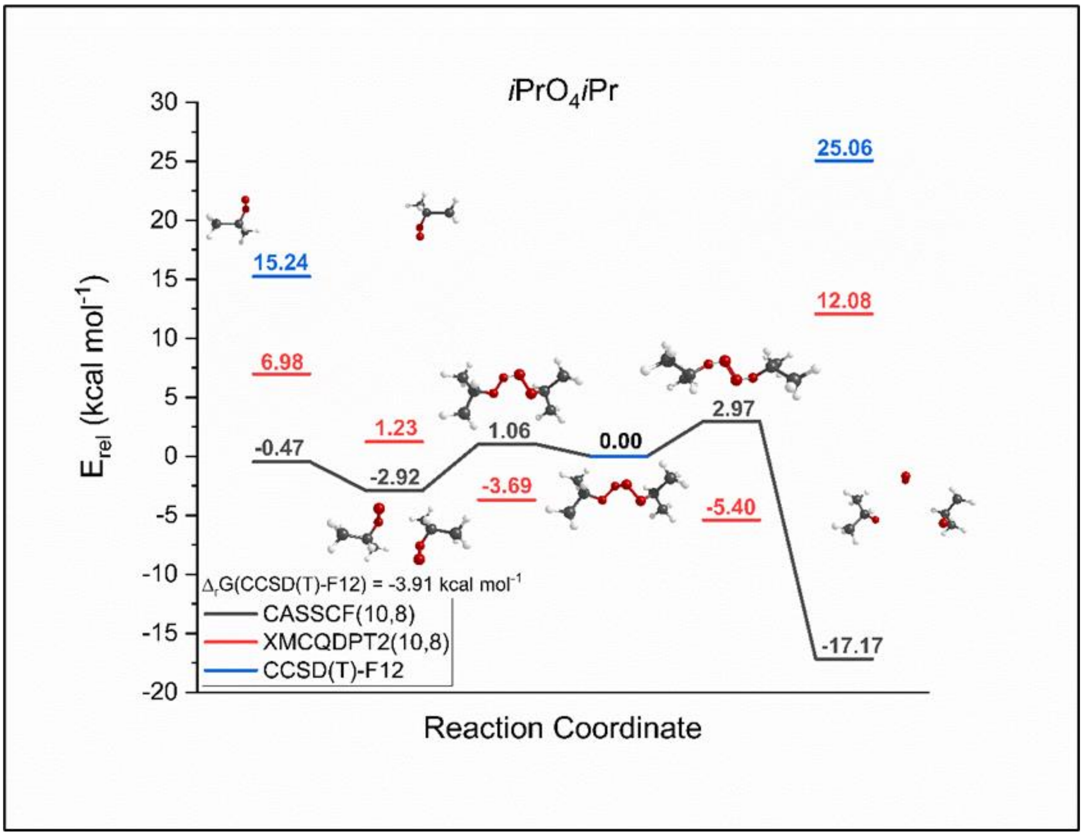

Figure S9. Reaction coordinate of the formation and decomposition of $iPrO_4iPr$.

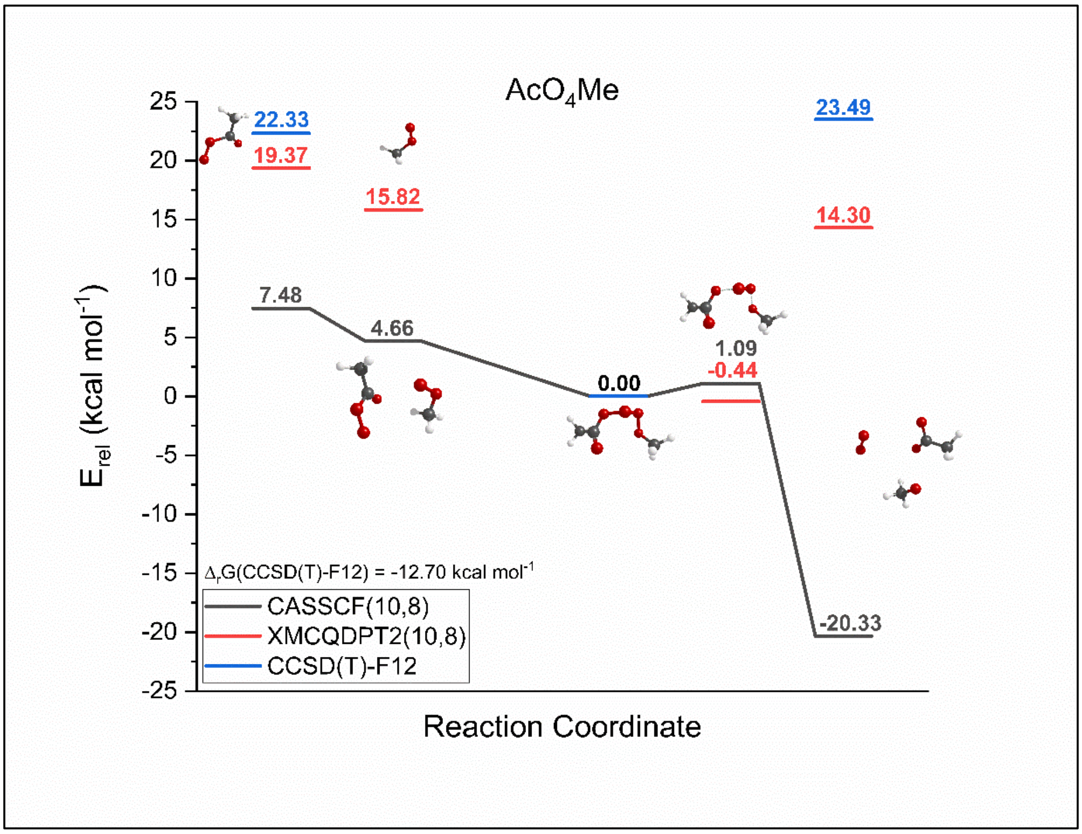

Figure S10. Reaction coordinate of the formation and decomposition of AcO_4Me.

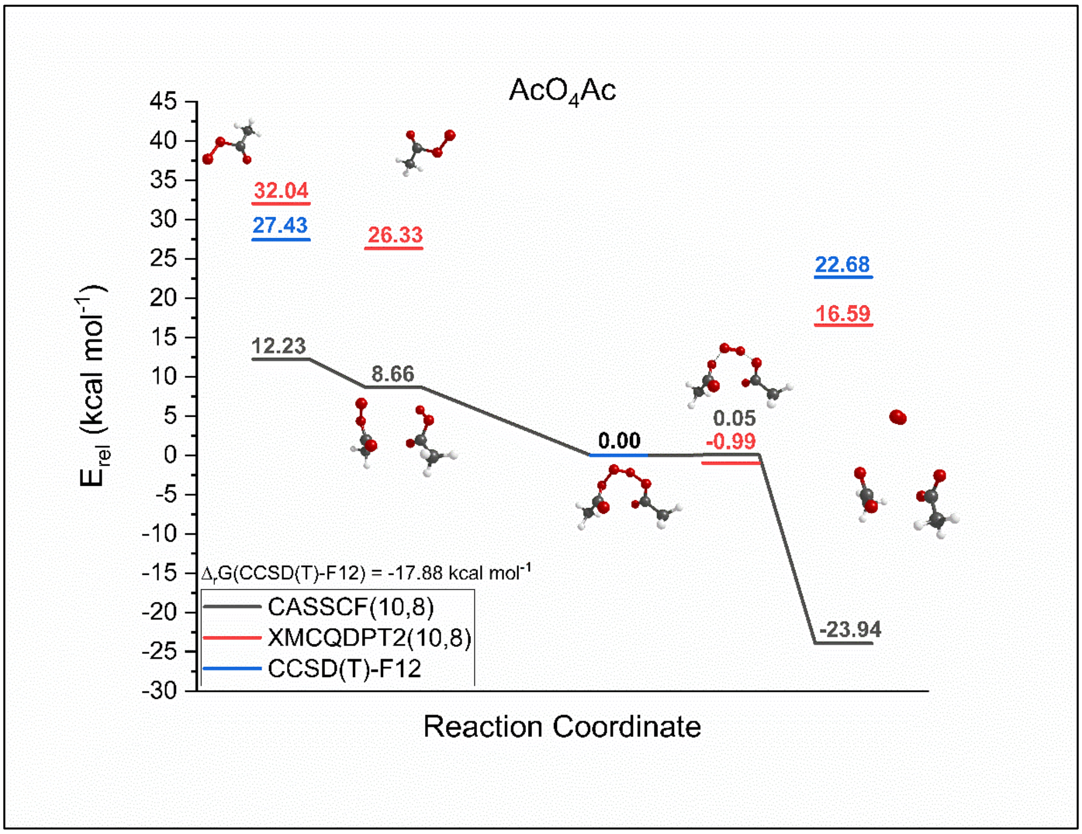

Figure S11. Reaction coordinate of the formation and decomposition of AcO_4Ac.

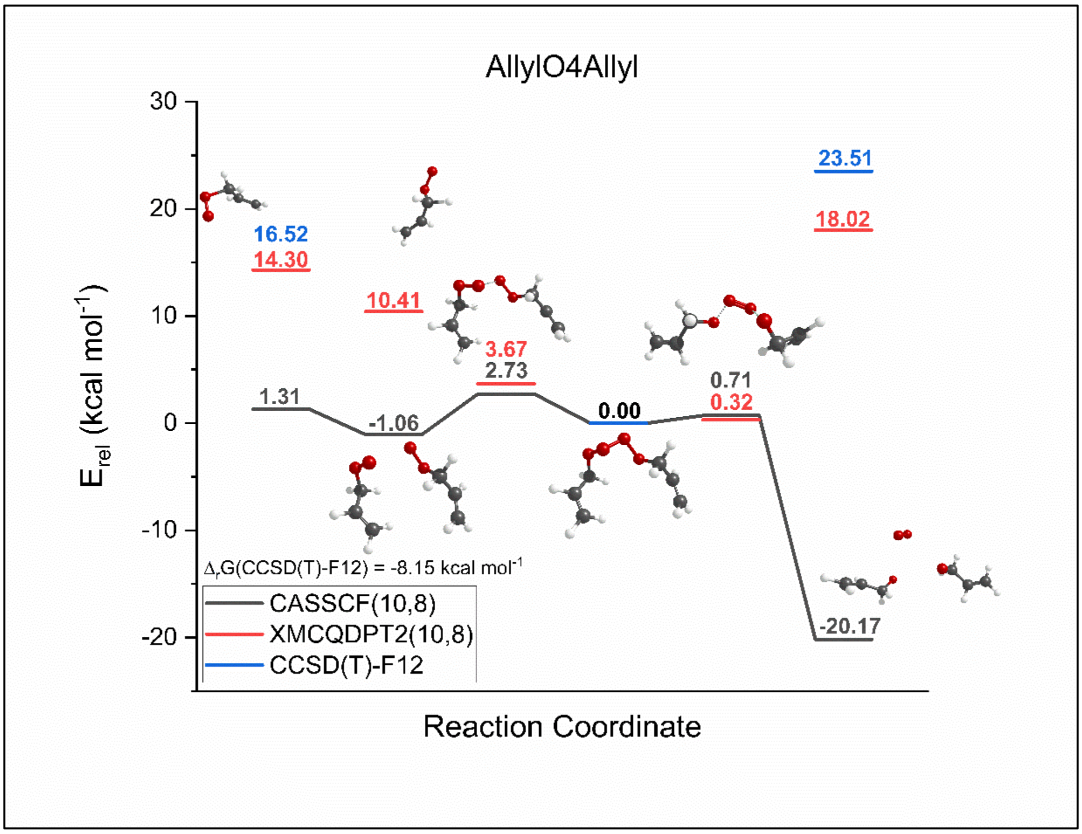

Figure S12. Reaction coordinate of the formation and decomposition of AllylO$_4$Allyl.

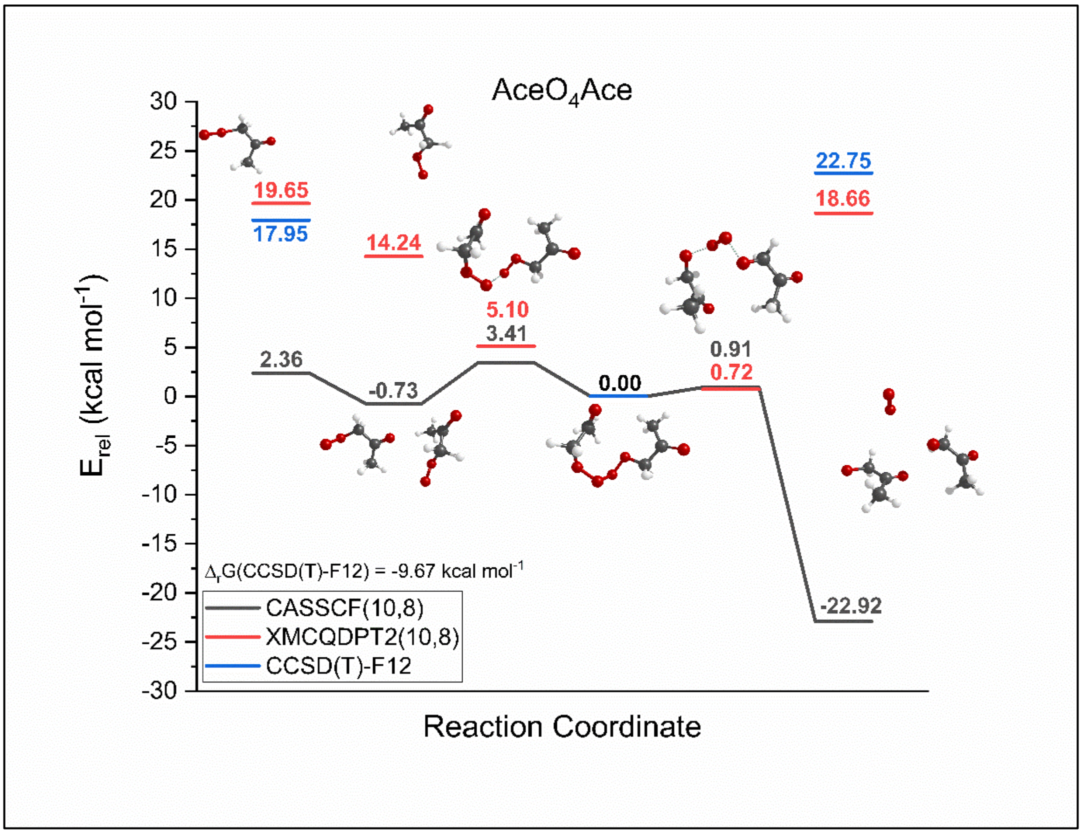

Figure S13. Reaction coordinate of the formation and decomposition of AceO$_4$Ace.

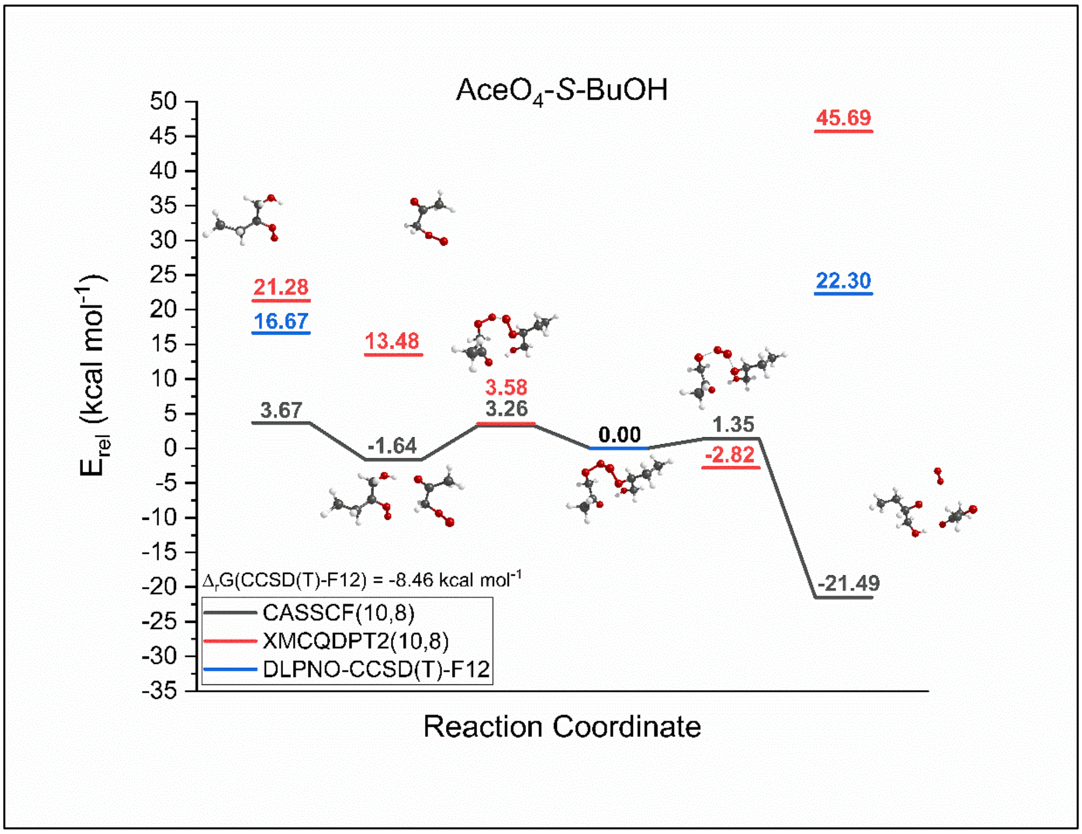

Figure S14. Reaction coordinate of the formation and decomposition of AceO$_4$-S-BuOH.

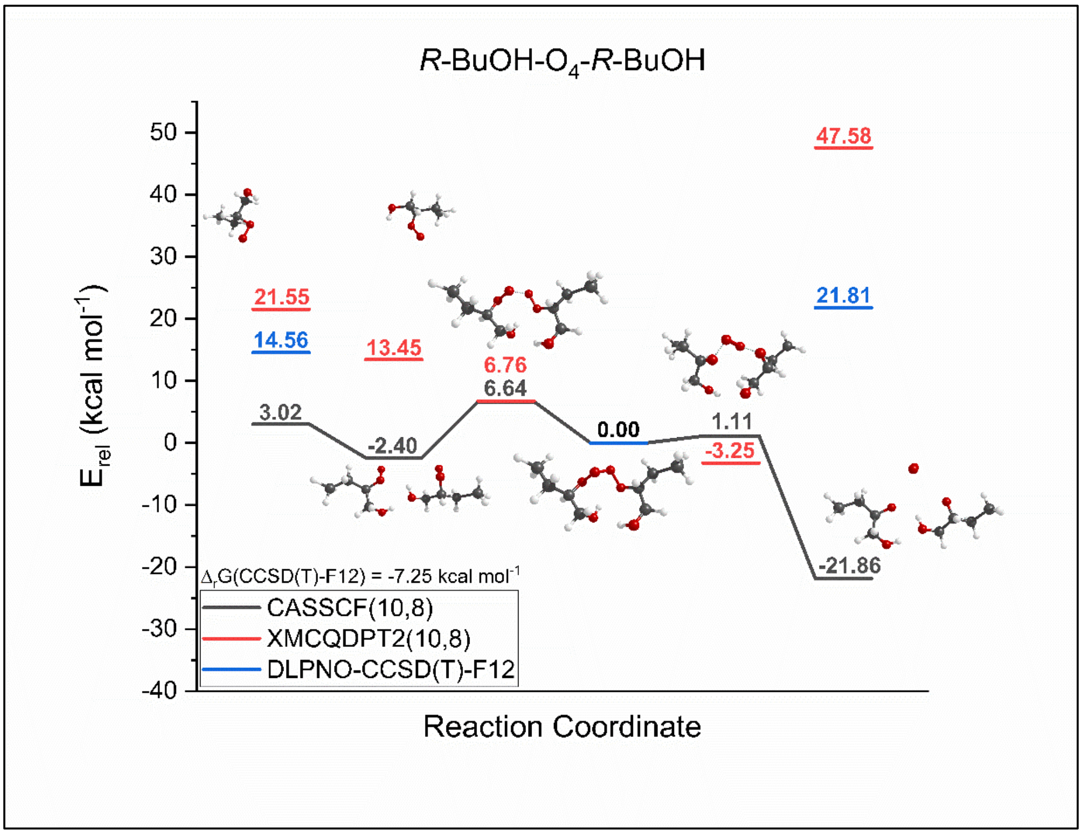

Figure S15. Reaction coordinate of the formation and decomposition of *R*-BuOH-O$_4$-*R*-BuOH.

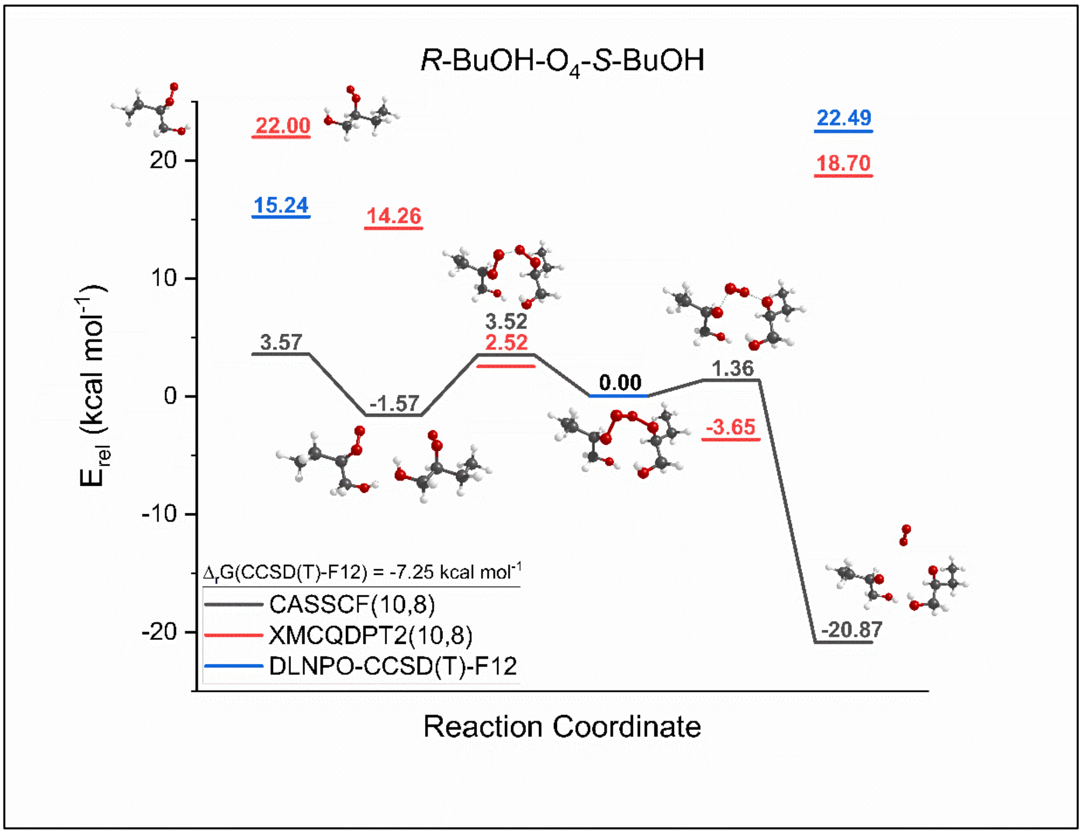

Figure S16. Reaction coordinate of the formation and decomposition of *R*-BuOH-O_4-*S*-BuOH.

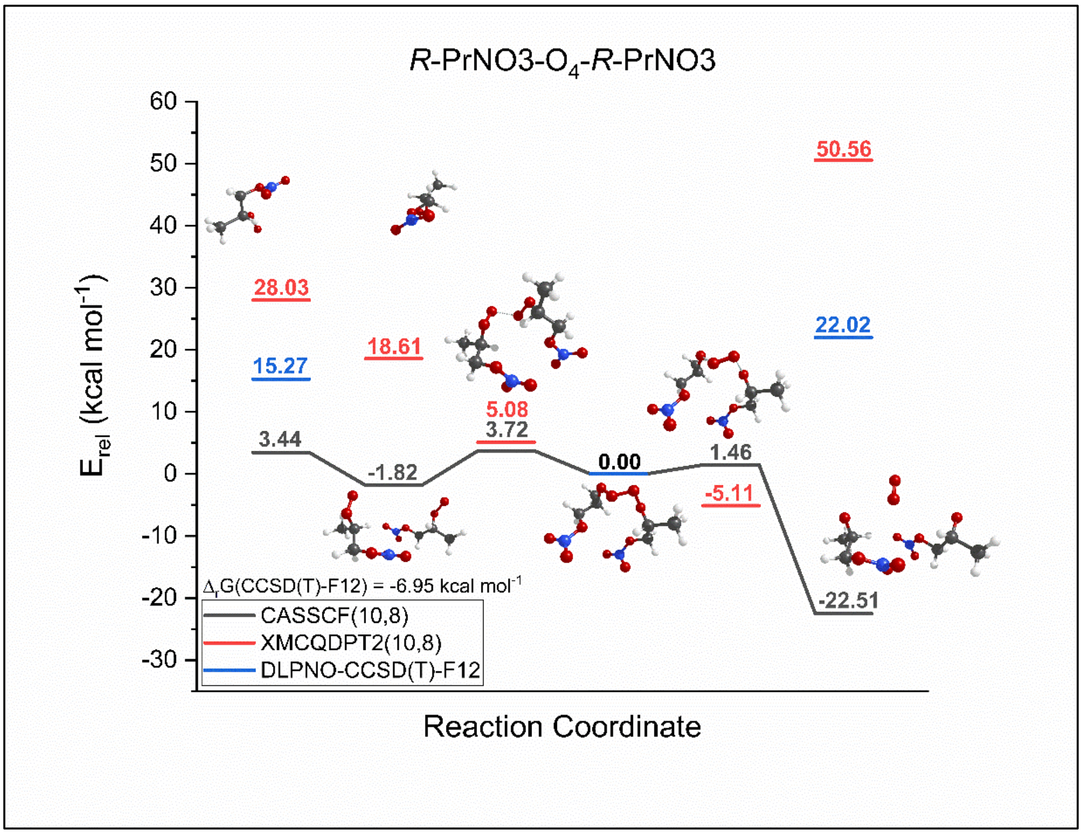

Figure S17. Reaction coordinate of the formation and decomposition of *R*-$PrNO_3$-O_4-*R*-$PrNO_3$.

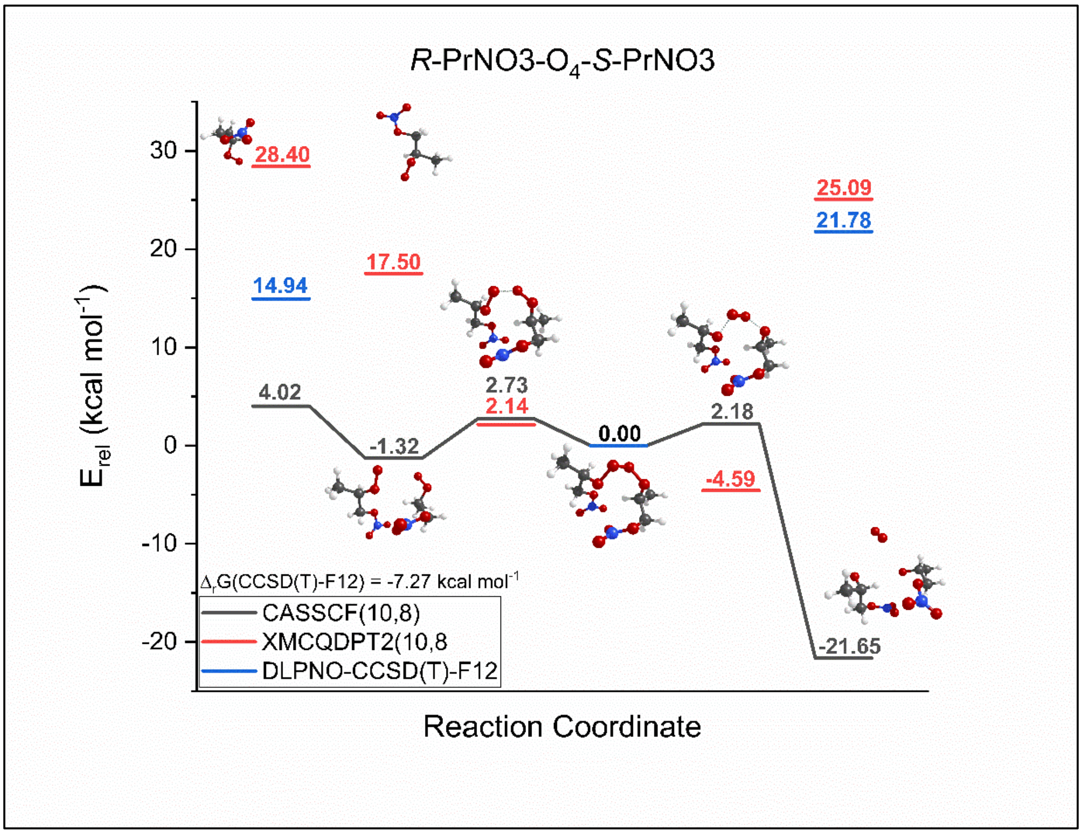

Figure S18. Reaction coordinate of the formation and decomposition of *R*-$PrNO_3$-O_4-*S*-$PrNO_3$.

S9. ωB97X-D / aug-cc-pVTZ Optimized Geometries

S9.1. Molecular oxygen

$^{3}O_2$ ($^{3}\Sigma_g^-$)

E(ωB97X-D/aug-cc-pVTZ) = -150.3342995 Eh
E(CCSD(T)-F12/cc-pVDZ-F12) = -150.1752595 Eh
E(DLPNO-CCSD(T)-F12/cc-pVTZ-F12) = -150.1871595 Eh
0 3

O	0.000000000000	0.000000000000	0.597995000000
O	0.000000000000	0.000000000000	-0.597995000000

$^{1}O_2$ ($^{1}\Delta_g$)

E(ωB97X-D/aug-cc-pVTZ) = -150.2730336 Eh
E(CCSD(T)-F12/cc-pVDZ-F12) = -150.1290506 Eh
E(DLPNO-CCSD(T)-F12/cc-pVTZ-F12) = -150.1369837 Eh
0 3

O	0.000000000000	0.000000000000	0.597508000000
O	0.000000000000	0.000000000000	-0.597508000000

S9.2. Alkoxyl radicals

HO (hydroxyl)

E(ωB97X-D/aug-cc-pVTZ) = -75.7407449 Eh
E(CCSD(T)-F12/cc-pVDZ-F12) = -75.6628772 Eh
0 1

O	0.000000000000	0.000000000000	0.107827000000
H	0.000000000000	0.000000000000	-0.862618000000

MeO (methoxyl)

E(ωB97X-D/aug-cc-pVTZ) = -115.0588082 Eh
E(CCSD(T)-F12/cc-pVDZ-F12) = -114.9129965 Eh
0 1

C	0.011022000000	-0.571078000000	0.000000000000
H	-1.055717000000	-0.862188000000	0.000000000000
H	0.450706000000	-1.002476000000	0.907699000000
H	0.450706000000	-1.002476000000	-0.907699000000
O	0.011022000000	0.786701000000	0.000000000000

EtO (ethoxyl)

E(ωB97X-D/aug-cc-pVTZ) = -154.3778286 Eh
E(CCSD(T)-F12/cc-pVDZ-F12) = -154.1696503 Eh
0 1

H	0.846568000000	-1.213903000000	0.887181000000
C	0.990175000000	-0.601531000000	0.000000000000
H	2.001831000000	-0.199059000000	0.000000000000
H	0.846568000000	-1.213903000000	-0.887181000000
C	0.000000000000	0.584929000000	0.000000000000

H	0.181161000000	1.185695000000	0.900349000000
H	0.181161000000	1.185695000000	-0.900349000000
O	-1.249793000000	0.044386000000	0.000000000000

*i*PrO (isopropoxyl)

E(ωB97X-D/aug-cc-pVTZ) = -193.6991494 Eh
E(CCSD(T)-F12/cc-pVDZ-F12) = -193.4285783 Eh
0 1

H	-1.043455000000	-1.741738000000	0.319939000000
C	-1.174555000000	-0.742111000000	-0.092232000000
H	-1.196702000000	-0.803826000000	-1.179056000000
H	-2.113428000000	-0.325501000000	0.264226000000
C	0.020772000000	0.128066000000	0.364069000000
H	-0.021444000000	0.170334000000	1.463008000000
C	1.350022000000	-0.455002000000	-0.099843000000
H	1.357896000000	-0.562094000000	-1.184746000000
H	1.516170000000	-1.433379000000	0.353056000000
H	2.171538000000	0.199421000000	0.188427000000
O	-0.231001000000	1.363883000000	-0.157102000000

AcO (acetyloxyl)

E(ωB97X-D/aug-cc-pVTZ) = -228.4222196 Eh
E(CCSD(T)-F12/cc-pVDZ-F12) = -228.1376216 Eh
0 1

C	-1.386240000000	0.009957000000	-0.000075000000
H	-1.757263000000	1.031983000000	-0.001725000000
H	-1.746297000000	-0.516353000000	0.882953000000
H	-1.746493000000	-0.519420000000	-0.881175000000
C	0.100201000000	0.002572000000	-0.000131000000
O	0.819126000000	1.024076000000	0.000087000000
O	0.801659000000	-1.032999000000	0.000061000000

AceO (acetonyloxyl)

E(ωB97X-D/aug-cc-pVTZ) = -267.7110855 Eh
E(CCSD(T)-F12/cc-pVDZ-F12) = -267.3661528 Eh
E(DLPNO-CCSD(T)-F12/cc-pVTZ-F12) = -267.3781794 Eh
0 1

H	1.900505000000	1.478173000000	0.083062000000
C	0.830197000000	1.295668000000	0.060964000000
H	0.336018000000	1.741241000000	0.923697000000
H	0.384768000000	1.753474000000	-0.823561000000
C	0.561214000000	-0.175639000000	0.015163000000
C	-0.944435000000	-0.611248000000	0.217498000000
H	-1.060636000000	-1.622804000000	-0.188015000000
H	-0.976008000000	-0.648597000000	1.324823000000
O	1.380711000000	-1.027053000000	-0.145647000000
O	-1.789024000000	0.307781000000	-0.239571000000

AllylO (allyloxyl)

E(ωB97X-D/aug-cc-pVTZ) = -192.4536152 Eh
E(CCSD(T)-F12/cc-pVDZ-F12) = -192.1880883 Eh
0 1

C	0.000000000000	0.919031000000	0.000000000000
H	-0.507439000000	1.878409000000	0.000000000000
C	1.320014000000	0.859014000000	0.000000000000
H	1.833891000000	-0.093364000000	0.000000000000
H	1.921985000000	1.756786000000	0.000000000000
C	-0.902890000000	-0.282151000000	0.000000000000
H	-1.599228000000	-0.248409000000	0.859355000000
H	-1.599228000000	-0.248409000000	-0.859355000000
O	-0.319091000000	-1.502548000000	0.000000000000

R-BuOH-O (*R*-1-hydroxy-butan-2-yloxyl)

E(ωB97X-D/aug-cc-pVTZ) = -308.2392184 Eh
E(CCSD(T)-F12/cc-pVDZ-F12) = -307.8303028 Eh
E(DLPNO-CCSD(T)-F12/cc-pVTZ-F12) = -307.8435064 Eh
0 1

H	2.059391000000	0.314933000000	-1.339207000000
C	2.120843000000	-0.459256000000	-0.573976000000
H	1.708613000000	-1.379373000000	-0.988239000000
H	3.174218000000	-0.637952000000	-0.362150000000
C	1.385715000000	-0.024381000000	0.689802000000
H	1.890045000000	0.837886000000	1.127684000000
H	1.411096000000	-0.821452000000	1.436477000000
C	-0.072040000000	0.367566000000	0.443472000000
C	-0.996872000000	-0.785465000000	0.006334000000
H	-0.967217000000	-1.577823000000	0.753957000000
H	-0.630328000000	-1.195510000000	-0.940947000000
O	-2.325935000000	-0.356265000000	-0.105932000000
H	-2.316581000000	0.461370000000	-0.610548000000
H	-0.507209000000	0.735624000000	1.396211000000
O	-0.230053000000	1.440204000000	-0.377446000000

S-BuOH-O (*S*-1-hydroxy-butan-2-yloxyl)

E(ωB97X-D/aug-cc-pVTZ) = -308.2392184 Eh
E(CCSD(T)-F12/cc-pVDZ-F12) = -307.8303028 Eh
E(DLPNO-CCSD(T)-F12/cc-pVTZ-F12) = -307.8435065 Eh
0 1

H	-1.708612000000	-1.379361000000	-0.988254000000
C	-2.120842000000	-0.459249000000	-0.573980000000
H	-2.059392000000	0.314948000000	-1.339203000000
H	-3.174217000000	-0.637949000000	-0.362156000000
C	-1.385715000000	-0.024388000000	0.689802000000
H	-1.411094000000	-0.821468000000	1.436468000000
H	-1.890046000000	0.837873000000	1.127694000000
C	0.072040000000	0.367563000000	0.443475000000
H	0.507207000000	0.735620000000	1.396216000000

C	0.996874000000	-0.785466000000	0.006336000000
H	0.630326000000	-1.195517000000	-0.940941000000
H	0.967227000000	-1.577821000000	0.753963000000
O	2.325934000000	-0.356260000000	-0.105940000000
H	2.316572000000	0.461381000000	-0.610546000000
O	0.230052000000	1.440202000000	-0.377440000000

R-PrNO3-O (*R*-2-oxyl-propyl nitrate)

E(ωB97X-D/aug-cc-pVTZ) = -473.3986351 Eh
E(CCSD(T)-F12/cc-pVDZ-F12) = -472.8345579 Eh
E(DLPNO-CCSD(T)-F12/cc-pVTZ-F12) = -472.8567238 Eh
0 1

H	0.288833000000	1.085103000000	1.219511000000
C	0.440201000000	0.047523000000	0.928395000000
H	0.840087000000	-0.512219000000	1.772173000000
C	1.399956000000	-0.059124000000	-0.260444000000
H	0.901132000000	0.375881000000	-1.147108000000
C	2.683847000000	0.736011000000	-0.012319000000
H	3.199640000000	0.353915000000	0.868898000000
H	2.458813000000	1.790058000000	0.145305000000
H	3.345856000000	0.638801000000	-0.869252000000
O	-0.817476000000	-0.589636000000	0.697066000000
N	-1.707739000000	0.140801000000	-0.081347000000
O	-1.354018000000	1.221946000000	-0.473240000000
O	-2.740628000000	-0.421921000000	-0.258085000000
O	1.634096000000	-1.343340000000	-0.634977000000

S-PrNO3-O (*S*-2-oxyl-propyl nitrate)

E(ωB97X-D/aug-cc-pVTZ) = -473.3988819 Eh
E(CCSD(T)-F12/cc-pVDZ-F12) = -472.8348028 Eh
E(DLPNO-CCSD(T)-F12/cc-pVTZ-F12) = -472.8565704 Eh
0 1

H	-0.227767000000	-1.486100000000	0.923433000000
C	-0.458719000000	-0.424069000000	0.898392000000
H	-0.889619000000	-0.122749000000	1.851660000000
C	-1.449597000000	-0.117986000000	-0.234253000000
C	-1.751157000000	1.370304000000	-0.412191000000
H	-0.836530000000	1.927380000000	-0.607330000000
H	-2.209546000000	1.767413000000	0.493432000000
H	-2.438692000000	1.509205000000	-1.243160000000
O	0.743003000000	0.353258000000	0.840958000000
N	1.675467000000	-0.070770000000	-0.101696000000
O	1.407597000000	-1.040971000000	-0.757175000000
O	2.649575000000	0.612724000000	-0.128264000000
H	-1.018758000000	-0.496249000000	-1.181616000000
O	-2.568990000000	-0.871636000000	-0.085048000000

S9.3. Peroxyl radicals

HO_2 (hydroperoxyl)

E(ωB97X-D/aug-cc-pVTZ) = -150.9209996 Eh
E(CCSD(T)-F12/cc-pVDZ-F12) = -150.7616069 Eh
0 1

O	0.055001000000	0.709921000000	0.000000000000
O	0.055001000000	-0.602084000000	0.000000000000
H	-0.880024000000	-0.862702000000	0.000000000000

MeO_2 (methylperoxyl)

E(ωB97X-D/aug-cc-pVTZ) = -190.2294049 Eh
E(CCSD(T)-F12/cc-pVDZ-F12) = -190.0062618 Eh
0 1

C	0.990142000000	-0.479485000000	0.000000000000
O	0.000000000000	0.560606000000	0.000000000000
O	-1.203998000000	0.067037000000	0.000000000000
H	1.950965000000	0.028364000000	0.000000000000
H	0.870084000000	-1.086298000000	0.894983000000
H	0.870084000000	-1.086298000000	-0.894983000000

EtO_2 (ethylperoxyl)

E(ωB97X-D/aug-cc-pVTZ) = -229.5505678 Eh
E(CCSD(T)-F12/cc-pVDZ-F12) = -229.2646667 Eh
0 1

C	0.487966000000	0.634658000000	0.253833000000
H	0.304982000000	0.705345000000	1.325007000000
H	0.807174000000	1.602412000000	-0.129394000000
O	-0.793536000000	0.417690000000	-0.385535000000
O	-1.449734000000	-0.554763000000	0.176643000000
C	1.462541000000	-0.466835000000	-0.089662000000
H	1.090143000000	-1.425394000000	0.266807000000
H	2.420853000000	-0.267840000000	0.389448000000
H	1.619965000000	-0.524873000000	-1.165756000000

iPrO_2 (isopropylperoxyl)

E(ωB97X-D/aug-cc-pVTZ) = -268.8722602 Eh
E(CCSD(T)-F12/cc-pVDZ-F12) = -268.5240335 Eh
0 1

H	1.309063000000	1.933993000000	-0.415279000000
C	0.480381000000	1.456944000000	0.107531000000
H	0.670650000000	1.511506000000	1.179768000000
H	-0.432864000000	2.002943000000	-0.119950000000
C	0.360078000000	0.015797000000	-0.337863000000
H	0.141908000000	-0.040848000000	-1.404584000000
C	1.551388000000	-0.842300000000	0.022793000000
H	1.723582000000	-0.827704000000	1.099157000000
H	2.441529000000	-0.453962000000	-0.470389000000
H	1.402741000000	-1.873170000000	-0.293912000000
O	-0.784789000000	-0.604125000000	0.328661000000

O	-1.916173000000	-0.150300000000	-0.119858000000

AcO_2 (acetylperoxyl)

E(ωB97X-D/aug-cc-pVTZ) = -303.5779813 Eh
E(CCSD(T)-F12/cc-pVDZ-F12) = -303.2214673 Eh
0 1

C	-1.449044000000	0.211363000000	0.000000000000
H	-1.693806000000	-0.386673000000	0.875633000000
H	-1.693806000000	-0.386673000000	-0.875633000000
H	-2.014499000000	1.137143000000	0.000000000000
C	0.000000000000	0.561599000000	0.000000000000
O	0.913965000000	-0.540886000000	0.000000000000
O	0.341763000000	-1.713974000000	0.000000000000
O	0.506319000000	1.629664000000	0.000000000000

$AceO_2$ (acetonylperoxyl)

E(ωB97X-D/aug-cc-pVTZ) = -342.8773129 Eh
E(CCSD(T)-F12/cc-pVDZ-F12) = -342.4575807 Eh
E(DLPNO-CCSD(T)-F12/cc-pVTZ-F12) = -342.4749486 Eh
0 1

C	-0.877617000000	1.389927000000	0.176610000000
H	-0.205046000000	1.810775000000	-0.572144000000
H	-0.443939000000	1.620184000000	1.150783000000
H	-1.864866000000	1.831899000000	0.083906000000
C	-0.977645000000	-0.094254000000	-0.010400000000
C	0.274144000000	-0.949157000000	0.186876000000
H	0.116210000000	-1.604775000000	1.042304000000
H	0.446684000000	-1.544059000000	-0.708060000000
O	1.459805000000	-0.202991000000	0.488635000000
O	1.965433000000	0.345686000000	-0.581206000000
O	-1.995530000000	-0.666836000000	-0.296841000000

$AllylO_2$ (allylperoxyl)

E(ωB97X-D/aug-cc-pVTZ) = -267.6234352 Eh
E(CCSD(T)-F12/cc-pVDZ-F12) = -267.2812879 Eh
0 1

C	2.230121000000	-0.126171000000	-0.198559000000
H	2.970364000000	-0.908028000000	-0.297486000000
H	2.547540000000	0.884147000000	-0.427564000000
C	0.995299000000	-0.392221000000	0.192342000000
H	0.688975000000	-1.407421000000	0.416113000000
C	-0.052367000000	0.652910000000	0.375058000000
H	0.289888000000	1.633797000000	0.049893000000
H	-0.408500000000	0.700593000000	1.404011000000
O	-1.212288000000	0.377754000000	-0.454577000000
O	-1.928535000000	-0.591529000000	0.034825000000

R-BuOH-O_2 (*R*-1-hydroxy-butan-2-ylperoxyl)

E(ωB97X-D/aug-cc-pVTZ) = -383.4105648 Eh
E(CCSD(T)-F12/cc-pVDZ-F12) = -382.9247177 Eh
E(DLPNO-CCSD(T)-F12/cc-pVTZ-F12) = -382.9428611 Eh
0 1

C	0.228812000000	1.142114000000	0.708419000000
H	1.113771000000	1.327111000000	1.327379000000
H	-0.625047000000	1.588701000000	1.218461000000
C	-0.002477000000	-0.362258000000	0.638023000000
H	-0.278514000000	-0.718289000000	1.632589000000
C	-1.020875000000	-0.817842000000	-0.392568000000
H	-1.065045000000	-1.907482000000	-0.365237000000
H	-0.666272000000	-0.530610000000	-1.382161000000
C	-2.402021000000	-0.226165000000	-0.141395000000
H	-3.125477000000	-0.644759000000	-0.839136000000
H	-2.396831000000	0.854725000000	-0.276441000000
H	-2.751901000000	-0.444631000000	0.869355000000
O	0.329311000000	1.775276000000	-0.540559000000
H	1.035045000000	1.336475000000	-1.022903000000
O	1.258955000000	-1.066033000000	0.412054000000
O	1.904188000000	-0.618785000000	-0.626092000000

S-BuOH-O_2 (*S*-1-hydroxy-butan-2-ylperoxyl)

E(ωB97X-D/aug-cc-pVTZ) = -383.4105648 Eh
E(CCSD(T)-F12/cc-pVDZ-F12) = -382.9247189 Eh
E(DLPNO-CCSD(T)-F12/cc-pVTZ-F12) = -382.9428611 Eh
0 1

C	-0.228812000000	1.142114000000	0.708419000000
H	0.625047000000	1.588701000000	1.218461000000
H	-1.113771000000	1.327111000000	1.327379000000
C	0.002477000000	-0.362258000000	0.638023000000
H	0.278514000000	-0.718289000000	1.632589000000
C	1.020875000000	-0.817841000000	-0.392568000000
H	0.666272000000	-0.530610000000	-1.382161000000
H	1.065045000000	-1.907482000000	-0.365237000000
C	2.402021000000	-0.226165000000	-0.141395000000
H	3.125476000000	-0.644759000000	-0.839136000000
H	2.751901000000	-0.444631000000	0.869355000000
H	2.396831000000	0.854725000000	-0.276440000000
O	-0.329311000000	1.775276000000	-0.540559000000
H	-1.035045000000	1.336475000000	-1.022903000000
O	-1.258955000000	-1.066033000000	0.412054000000
O	-1.904188000000	-0.618785000000	-0.626092000000

R-PrNO_3-O_2 (*R*-2-peroxyl-propyl nitrate)

E(ωB97X-D/aug-cc-pVTZ) = -548.5695601 Eh
E(CCSD(T)-F12/cc-pVDZ-F12) = -547.9285317 Eh
E(DLPNO-CCSD(T)-F12/cc-pVDZ-F12) = -547.9556782 Eh
0 1

H	2.714155000000	1.143501000000	0.873423000000
C	2.135422000000	1.453638000000	0.004698000000
H	1.678695000000	2.420862000000	0.209708000000
H	2.808751000000	1.562576000000	-0.842949000000
C	1.057902000000	0.438818000000	-0.311932000000
C	0.155971000000	0.182361000000	0.878255000000
H	-0.178564000000	1.124258000000	1.306403000000
H	0.694756000000	-0.390378000000	1.630316000000
O	-0.964505000000	-0.649990000000	0.571398000000
N	-2.024020000000	0.001537000000	-0.058789000000
O	-1.911105000000	1.179550000000	-0.268769000000
O	-2.930659000000	-0.725931000000	-0.307984000000
H	0.474166000000	0.745800000000	-1.177419000000
O	1.652173000000	-0.814260000000	-0.767472000000
O	2.389146000000	-1.372654000000	0.146066000000

S-$PrNO_3$-O_2 (*S*-2-peroxyl-propyl nitrate)

E(ωB97X-D/aug-cc-pVTZ) = -548.5695601 Eh
E(CCSD(T)-F12/cc-pVDZ-F12) = -547.9285318 Eh
E(DLPNO-CCSD(T)-F12/cc-pVDZ-F12) = -547.9556781 Eh
0 1

H	-1.678695000000	2.420862000000	0.209708000000
C	-2.135422000000	1.453638000000	0.004698000000
H	-2.714154000000	1.143501000000	0.873423000000
H	-2.808751000000	1.562575000000	-0.842948000000
C	-1.057902000000	0.438818000000	-0.311932000000
H	-0.474166000000	0.745800000000	-1.177419000000
C	-0.155971000000	0.182361000000	0.878255000000
H	-0.694756000000	-0.390378000000	1.630316000000
H	0.178564000000	1.124258000000	1.306403000000
O	-1.652173000000	-0.814260000000	-0.767472000000
O	-2.389147000000	-1.372654000000	0.146066000000
O	0.964505000000	-0.649990000000	0.571398000000
N	2.024020000000	0.001537000000	-0.058789000000
O	1.911105000000	1.179550000000	-0.268769000000
O	2.930659000000	-0.725931000000	-0.307985000000

S9.4. Pre-reactive complexes

MeO_2…MeO_2

E(ωB97X-D/aug-cc-pVTZ) = -380.4640635 Eh
0 1

H	1.247441000000	-1.271925000000	0.344376000000
C	0.648759000000	-2.007161000000	-0.189518000000
O	-0.648759000000	-1.446834000000	-0.453832000000
O	-1.289369000000	-1.190340000000	0.649034000000
O	1.289369000000	1.190340000000	0.649034000000
O	0.648759000000	1.446834000000	-0.453832000000
C	-0.648759000000	2.007161000000	-0.189518000000
H	-1.247441000000	1.271925000000	0.344376000000

H	1.077006000000	-2.221736000000	-1.164832000000
H	-1.077006000000	2.221736000000	-1.164832000000
H	-0.528240000000	2.915925000000	0.395949000000
H	0.528240000000	-2.915925000000	0.395949000000

$EtO_2 \ldots EtO_2$

E(ωB97X-D/aug-cc-pVTZ) = -459.1080184 Eh

0 1

H	1.345149000000	-1.368212000000	1.269638000000
C	2.262599000000	-1.000778000000	0.813901000000
H	2.823157000000	-1.856421000000	0.440064000000
H	2.864138000000	-0.499715000000	1.571111000000
C	1.952218000000	-0.075818000000	-0.334146000000
H	1.317021000000	-0.545552000000	-1.083994000000
H	2.846516000000	0.323890000000	-0.811177000000
O	1.217221000000	1.055368000000	0.201258000000
O	0.834747000000	1.872741000000	-0.734302000000
O	-0.834801000000	-1.872754000000	-0.734292000000
O	-1.217190000000	-1.055349000000	0.201270000000
C	-1.952222000000	0.075811000000	-0.334135000000
H	-1.317049000000	0.545552000000	-1.084001000000
H	-2.846523000000	-0.323919000000	-0.811143000000
C	-2.262574000000	1.000779000000	0.813911000000
H	-2.864085000000	0.499723000000	1.571150000000
H	-2.823147000000	1.856414000000	0.440081000000
H	-1.345111000000	1.368223000000	1.269614000000

$iPrO_2 \ldots iPrO_2$

E(ωB97X-D/aug-cc-pVTZ) = -537.7520324 Eh

0 1

C	1.929261000000	-0.252648000000	-0.110215000000
C	3.250756000000	-0.035276000000	-0.813886000000
C	2.011666000000	-1.147721000000	1.103897000000
O	1.429708000000	1.032615000000	0.385969000000
O	1.022755000000	1.808018000000	-0.573783000000
O	-1.022689000000	-1.807965000000	-0.573821000000
O	-1.429773000000	-1.032655000000	0.385949000000
C	-1.929247000000	0.252653000000	-0.110190000000
C	-3.250676000000	0.035331000000	-0.813999000000
C	-2.011764000000	1.147649000000	1.103973000000
H	1.171859000000	-0.611175000000	-0.807545000000
H	3.135236000000	0.669308000000	-1.635118000000
H	3.605749000000	-0.982819000000	-1.218156000000
H	3.998927000000	0.346148000000	-0.118305000000
H	1.038055000000	-1.247177000000	1.579661000000
H	2.342682000000	-2.138710000000	0.796847000000
H	2.726737000000	-0.753715000000	1.826645000000
H	-1.171775000000	0.611210000000	-0.807429000000
H	-3.605632000000	0.982902000000	-1.218236000000
H	-3.135078000000	-0.669196000000	-1.635269000000

H	-3.998911000000	-0.346143000000	-0.118514000000
H	-2.342728000000	2.138663000000	0.796949000000
H	-1.038202000000	1.247055000000	1.579847000000
H	-2.726920000000	0.753612000000	1.826620000000

$AceO_2 \ldots AceO_2$

E(ωB97X-D/aug-cc-pVTZ) = -685.7591567 Eh

0 1

C	-3.514619000000	-0.333811000000	0.010032000000
O	-4.149995000000	-1.348818000000	-0.098497000000
C	-2.044552000000	-0.469597000000	0.403263000000
H	-1.984914000000	-0.938524000000	1.384128000000
H	-1.524321000000	-1.085834000000	-0.328324000000
C	-4.087003000000	1.035357000000	-0.206349000000
H	-3.588579000000	1.513981000000	-1.050445000000
H	-3.902445000000	1.665438000000	0.664852000000
H	-5.152316000000	0.953021000000	-0.398499000000
O	-1.347936000000	0.777968000000	0.535556000000
O	-1.026877000000	1.275365000000	-0.626331000000
O	1.026918000000	-1.275211000000	-0.626458000000
O	1.347935000000	-0.777974000000	0.535510000000
C	2.044576000000	0.469594000000	0.403398000000
H	1.524371000000	1.085953000000	-0.328104000000
H	1.984969000000	0.938382000000	1.384333000000
C	3.514616000000	0.333776000000	0.010119000000
O	4.150082000000	1.348753000000	-0.098164000000
C	4.086877000000	-1.035404000000	-0.206492000000
H	5.152203000000	-0.953133000000	-0.398600000000
H	3.588425000000	-1.513817000000	-1.050692000000
H	3.902232000000	-1.665633000000	0.664585000000

S9.5. Formation transition state structures

[MeOO···OOMe]‡

E(ωB97X-D/aug-cc-pVTZ) = -380.4579663 Eh

$\tilde{\nu}^{\ddagger}$ = -155.44 cm^{-1}

0 1

H	-1.277629000000	-0.865322000000	1.288039000000
C	-1.857566000000	-0.755650000000	0.374321000000
O	-1.063818000000	-0.175722000000	-0.660148000000
O	-0.710381000000	1.085491000000	-0.295631000000
O	0.710380000000	1.085491000000	0.295630000000
O	1.063819000000	-0.175722000000	0.660149000000
C	1.857566000000	-0.755650000000	-0.374321000000
H	1.277629000000	-0.865322000000	-1.288039000000
H	-2.144098000000	-1.731587000000	-0.012989000000
H	2.144096000000	-1.731587000000	0.012989000000
H	2.744080000000	-0.147343000000	-0.549752000000
H	-2.744079000000	-0.147343000000	0.549752000000

[EtOO···OOEt]‡

E(ωB97X-D/aug-cc-pVTZ) = -459.1004285 Eh

$\tilde{\nu}^{\ddagger}$ = -118.48 cm^{-1}

0 1

H	1.257627000000	2.182284000000	-0.175621000000
C	2.167078000000	1.602794000000	-0.020422000000
H	2.764527000000	2.099804000000	0.742745000000
H	2.735779000000	1.592104000000	-0.949286000000
C	1.839682000000	0.200196000000	0.431910000000
H	1.240983000000	0.196506000000	1.340918000000
H	2.739941000000	-0.395903000000	0.588103000000
O	1.077640000000	-0.400161000000	-0.628650000000
O	0.720735000000	-1.660802000000	-0.276439000000
O	-0.720267000000	-1.660881000000	0.276559000000
O	-1.077381000000	-0.400255000000	0.628602000000
C	-1.839471000000	0.199863000000	-0.432057000000
H	-1.240485000000	0.196772000000	-1.340881000000
H	-2.739290000000	-0.396784000000	-0.588676000000
C	-2.167907000000	1.602146000000	0.020498000000
H	-2.736971000000	1.590873000000	0.949131000000
H	-2.765348000000	2.098983000000	-0.742788000000
H	-1.258874000000	2.182157000000	0.176204000000

[iPrOO···OOiPr]‡

E(ωB97X-D/aug-cc-pVTZ) = -537.7433321 Eh

$\tilde{\nu}^{\ddagger}$ = -106.60 cm^{-1}

0 1

C	1.892520000000	0.326606000000	0.224505000000
C	3.206791000000	-0.421239000000	0.311534000000
C	2.048618000000	1.749259000000	-0.268030000000
O	1.011676000000	-0.293810000000	-0.745976000000
O	0.686664000000	-1.549778000000	-0.354166000000
O	-0.686667000000	-1.549787000000	0.354127000000
O	-1.011680000000	-0.293828000000	0.745965000000
C	-1.892528000000	0.326605000000	-0.224500000000
C	-3.206819000000	-0.421209000000	-0.311488000000
C	-2.048577000000	1.749268000000	0.268022000000
H	1.376598000000	0.309919000000	1.184343000000
H	3.044441000000	-1.454066000000	0.612347000000
H	3.851361000000	0.053924000000	1.051396000000
H	3.717836000000	-0.410251000000	-0.651945000000
H	1.083403000000	2.247735000000	-0.339080000000
H	2.676833000000	2.308628000000	0.423933000000
H	2.521462000000	1.764993000000	-1.250460000000
H	-1.376629000000	0.309893000000	-1.184350000000
H	-3.851395000000	0.053953000000	-1.051347000000
H	-3.044499000000	-1.454047000000	-0.612284000000
H	-3.717844000000	-0.410191000000	0.652001000000
H	-2.676843000000	2.308628000000	-0.423903000000
H	-1.083355000000	2.247740000000	0.338990000000

H	-2.521347000000	1.765021000000	1.250488000000

[AceOO···OOAce]‡

E(ωB97X-D/aug-cc-pVTZ) = -685.7559014 Eh

$\tilde{\nu}^{\ddagger}$ = -128.01 cm^{-1}

0 1

C	-3.308796000000	0.186297000000	-0.245181000000
O	-4.061103000000	1.053587000000	-0.607189000000
C	-1.880914000000	0.218579000000	-0.780871000000
H	-1.910982000000	-0.035342000000	-1.841630000000
H	-1.474575000000	1.221891000000	-0.672567000000
C	-3.711481000000	-0.931798000000	0.671192000000
H	-3.167348000000	-0.847373000000	1.613067000000
H	-3.440521000000	-1.895593000000	0.238802000000
H	-4.780379000000	-0.880701000000	0.855001000000
O	-0.999492000000	-0.745206000000	-0.208598000000
O	-0.676375000000	-0.370494000000	1.057845000000
O	0.676375000000	0.370487000000	1.057847000000
O	0.999492000000	0.745208000000	-0.208593000000
C	1.880914000000	-0.218573000000	-0.780873000000
H	1.474574000000	-1.221886000000	-0.672577000000
H	1.910982000000	0.035356000000	-1.841630000000
C	3.308796000000	-0.186295000000	-0.245182000000
O	4.061103000000	-1.053582000000	-0.607198000000
C	3.711481000000	0.931792000000	0.671200000000
H	4.780379000000	0.880694000000	0.855009000000
H	3.167348000000	0.847358000000	1.613074000000
H	3.440520000000	1.895590000000	0.238819000000

S9.6. Tetroxides

HO_4H (tetraoxidane)

E(ωB97X-D/aug-cc-pVTZ) = -301.8616105 Eh

E(CCSD(T)-F12/cc-pVDZ-F12) = -301.5537685 Eh

0 1

H	1.176054000000	1.221530000000	0.230707000000
O	1.465252000000	0.392037000000	-0.171123000000
O	0.575652000000	-0.536593000000	0.391402000000
O	-0.574341000000	-0.554905000000	-0.378652000000
O	-1.380246000000	0.527972000000	0.032540000000
H	-1.866583000000	0.150387000000	0.775963000000

MeO_4H (1-methyltetraoxidane)

E(ωB97X-D/aug-cc-pVTZ) = -341.1670368 Eh

E(CCSD(T)-F12/cc-pVDZ-F12) = -340.7945094 Eh

0 1

C	1.900396000000	0.165526000000	0.384165000000
H	1.504207000000	0.198792000000	1.399766000000
H	2.578945000000	1.001933000000	0.226789000000

H	2.421697000000	-0.775392000000	0.207663000000
O	0.869244000000	0.353661000000	-0.572348000000
O	-0.039115000000	-0.721849000000	-0.460737000000
O	-0.946594000000	-0.401663000000	0.535572000000
O	-1.932334000000	0.429975000000	-0.023052000000
H	-1.516833000000	1.300510000000	0.025318000000

MeO_4Me (1,4-dimethyltetraoxidane)

E(ωB97X-D/aug-cc-pVTZ) = -380.4716606 Eh
E(CCSD(T)-F12/cc-pVDZ-F12) = -380.0382595 Eh
0 1

H	0.010503000000	3.149773000000	-0.864824000000
C	-0.066186000000	2.439228000000	-0.044018000000
H	0.415553000000	2.843785000000	0.846990000000
H	-1.116002000000	2.219636000000	0.152571000000
O	0.615476000000	1.285785000000	-0.506006000000
O	0.615476000000	0.326990000000	0.522178000000
O	-0.615476000000	-0.326990000000	0.522178000000
O	-0.615476000000	-1.285785000000	-0.506006000000
C	0.066186000000	-2.439228000000	-0.044018000000
H	-0.010503000000	-3.149773000000	-0.864824000000
H	-0.415553000000	-2.843785000000	0.846990000000
H	1.116002000000	-2.219636000000	0.152571000000

EtO_4Et (1,4-diethyltetraoxidane)

E(ωB97X-D/aug-cc-pVTZ) = -459.1127590 Eh
E(CCSD(T)-F12/cc-pVDZ-F12) = -458.5534596 Eh
0 1

H	-3.062699000000	1.740707000000	-0.603355000000
C	-3.462816000000	0.850810000000	-0.119862000000
H	-4.363116000000	0.544573000000	-0.651336000000
H	-3.735502000000	1.102480000000	0.904050000000
C	-2.454927000000	-0.275222000000	-0.149488000000
H	-2.167434000000	-0.520803000000	-1.173432000000
H	-2.838545000000	-1.172280000000	0.341229000000
O	-1.309187000000	0.191268000000	0.561171000000
O	-0.353118000000	-0.834795000000	0.602586000000
O	0.353117000000	-0.834792000000	-0.602589000000
O	1.309186000000	0.191270000000	-0.561170000000
C	2.454926000000	-0.275222000000	0.149489000000
H	2.167433000000	-0.520803000000	1.173433000000
H	2.838542000000	-1.172280000000	-0.341229000000
C	3.462817000000	0.850808000000	0.119863000000
H	3.735504000000	1.102478000000	-0.904049000000
H	4.363117000000	0.544570000000	0.651337000000
H	3.062701000000	1.740706000000	0.603356000000

iPrO_4*i*Pr (1,4-diisopropyltetraoxidane)

E(ωB97X-D/aug-cc-pVTZ) = -537.7548874 Eh

E(CCSD(T)-F12/cc-pVDZ-F12) = -537.0723477 Eh
0 1

C	2.454002000000	0.005478000000	0.272974000000
C	3.213093000000	-1.103976000000	-0.428848000000
C	3.279795000000	1.260945000000	0.470429000000
O	1.325718000000	0.441874000000	-0.502775000000
O	0.381213000000	-0.592174000000	-0.586110000000
O	-0.381185000000	-0.592201000000	0.585986000000
O	-1.325686000000	0.441856000000	0.502704000000
C	-2.454033000000	0.005464000000	-0.272955000000
C	-3.213089000000	-1.103968000000	0.428939000000
C	-3.279823000000	1.260941000000	-0.470370000000
H	2.076534000000	-0.340766000000	1.237992000000
H	2.572721000000	-1.966961000000	-0.600348000000
H	4.056544000000	-1.421792000000	0.184679000000
H	3.594679000000	-0.753827000000	-1.388622000000
H	2.699261000000	2.031790000000	0.973870000000
H	4.155099000000	1.030081000000	1.076584000000
H	3.620334000000	1.649403000000	-0.489878000000
H	-2.076641000000	-0.340800000000	-1.237995000000
H	-4.056588000000	-1.421783000000	-0.184522000000
H	-2.572716000000	-1.966958000000	0.600408000000
H	-3.594602000000	-0.753798000000	1.388735000000
H	-4.155176000000	1.030079000000	-1.076454000000
H	-2.699315000000	2.031768000000	-0.973868000000
H	-3.620283000000	1.649420000000	0.489956000000

AcO_4Me (1-(methyltetraoxidaneyl)ethan-1-one)

E(ωB97X-D/aug-cc-pVTZ) = -493.8305443 Eh
E(CCSD(T)-F12/cc-pVDZ-F12) = -493.2633169 Eh
0 1

H	-3.220663000000	-0.764557000000	0.417754000000
C	-2.714676000000	0.180185000000	0.601044000000
H	-3.343919000000	1.015941000000	0.312081000000
H	-2.494786000000	0.246481000000	1.666562000000
C	-1.421548000000	0.264229000000	-0.156081000000
O	-0.834336000000	-0.978767000000	-0.133559000000
O	0.435696000000	-0.964552000000	-0.772568000000
O	1.385944000000	-1.080178000000	0.215202000000
O	1.558344000000	0.168543000000	0.840996000000
C	2.296803000000	1.022729000000	-0.020859000000
H	3.261554000000	0.578142000000	-0.266322000000
H	2.438908000000	1.931181000000	0.560771000000
H	1.722827000000	1.251230000000	-0.917959000000
O	-0.961572000000	1.222296000000	-0.689760000000

AcO_4Ac (1,4-diacetyltetraoxidane)

E(ωB97X-D/aug-cc-pVTZ) = -607.1876076 Eh
E(CCSD(T)-F12/cc-pVDZ-F12) = -606.4866414 Eh
0 1

C	0.050348000000	1.589051000000	-0.408273000000
C	1.036534000000	2.317294000000	-1.268628000000
H	1.704576000000	1.570868000000	-1.699641000000
H	1.632712000000	3.019653000000	-0.692005000000
H	0.501232000000	2.825083000000	-2.064774000000
O	-1.036534000000	1.212398000000	-0.712668000000
O	0.630797000000	1.384508000000	0.821702000000
O	-0.208876000000	0.646748000000	1.705694000000
O	0.208876000000	-0.646748000000	1.705694000000
O	-0.630797000000	-1.384508000000	0.821702000000
C	-0.050348000000	-1.589051000000	-0.408273000000
O	1.036534000000	-1.212398000000	-0.712668000000
C	-1.036534000000	-2.317294000000	-1.268628000000
H	-1.632712000000	-3.019653000000	-0.692005000000
H	-0.501232000000	-2.825083000000	-2.064774000000
H	-1.704576000000	-1.570868000000	-1.699641000000

AllylO$_4$Allyl (1,4-diallyltetraoxidane)

E(ωB97X-D/aug-cc-pVTZ) = -535.2610032 Eh
E(CCSD(T)-F12/cc-pVDZ-F12) = -534.5888946 Eh
0 1

C	3.191071000000	0.947432000000	-0.052474000000
H	4.055152000000	1.352053000000	0.456530000000
H	2.761498000000	1.542901000000	-0.849026000000
C	2.685486000000	-0.230030000000	0.275321000000
H	3.122841000000	-0.810551000000	1.079679000000
C	1.524773000000	-0.852176000000	-0.426967000000
H	1.806145000000	-1.804289000000	-0.884362000000
H	1.120816000000	-0.183979000000	-1.190015000000
O	0.532491000000	-1.103309000000	0.570174000000
O	-0.504890000000	-1.871711000000	-0.006851000000
O	-1.320188000000	-1.033471000000	-0.752420000000
O	-2.348923000000	-0.537747000000	0.069482000000
C	-1.932936000000	0.670836000000	0.714049000000
H	-1.173476000000	0.456225000000	1.465557000000
H	-2.851483000000	0.979121000000	1.218076000000
C	-1.473144000000	1.721947000000	-0.241370000000
H	-2.155193000000	1.966614000000	-1.048475000000
C	-0.314312000000	2.348971000000	-0.125580000000
H	-0.023627000000	3.131817000000	-0.813296000000
H	0.383775000000	2.098114000000	0.664377000000

AceO$_4$Ace (1,1’-tetraoxidanediylbis(propan-2-one))

E(ωB97X-D/aug-cc-pVTZ) = -685.7700945 Eh
E(CCSD(T)-F12/cc-pVDZ-F12) = -684.9437577 Eh
E(DLPNO-CCSD(T)-F12/cc-pVTZ-F12) = -684.9718217 Eh
0 1

C	1.491632000000	1.302084000000	-0.256335000000
O	0.799276000000	2.104885000000	-0.831340000000
C	2.034626000000	0.112438000000	-1.040623000000

H	1.362580000000	-0.122134000000	-1.864089000000
H	3.002635000000	0.405933000000	-1.456268000000
C	1.926234000000	1.455082000000	1.170932000000
H	3.016716000000	1.471959000000	1.222115000000
H	1.596628000000	0.599060000000	1.757252000000
H	1.519202000000	2.374765000000	1.580527000000
O	2.312430000000	-1.049349000000	-0.271454000000
O	1.182654000000	-1.893482000000	-0.273470000000
O	0.454335000000	-1.688130000000	0.883117000000
O	-0.422573000000	-0.596537000000	0.681834000000
C	-1.568181000000	-1.046604000000	-0.026577000000
H	-1.300719000000	-1.353094000000	-1.039349000000
H	-2.024592000000	-1.889882000000	0.494918000000
C	-2.602368000000	0.072520000000	-0.124247000000
O	-3.612364000000	-0.162072000000	-0.734901000000
C	-2.314598000000	1.384560000000	0.538435000000
H	-2.073695000000	1.229666000000	1.590937000000
H	-3.176039000000	2.037739000000	0.436084000000
H	-1.436856000000	1.842991000000	0.078077000000

AceO$_4$-*S*-BuOH (*S*-1-((1-hydroxybutan-2-yl)tetraoxidaneyl)propan-2-one)

E(ωB97X-D/aug-cc-pVTZ) = -726.3077969 Eh

E(DLPNO-CCSD(T)-F12/cc-pVTZ-F12) = -725.4443752 Eh

0 1

C	2.766290000000	-0.015887000000	1.493358000000
H	3.669280000000	-0.579076000000	1.254612000000
H	2.025852000000	-0.739375000000	1.834992000000
H	2.962314000000	0.713694000000	2.273245000000
C	2.241158000000	0.674408000000	0.273644000000
O	1.965814000000	1.852629000000	0.251912000000
C	2.120479000000	-0.140500000000	-1.006650000000
H	1.355782000000	0.288173000000	-1.652105000000
H	3.085903000000	-0.087226000000	-1.518519000000
O	1.886384000000	-1.526826000000	-0.800070000000
O	0.504362000000	-1.789589000000	-0.883700000000
O	-0.063244000000	-1.682730000000	0.375963000000
O	-0.331654000000	-0.318545000000	0.625733000000
C	-1.560443000000	0.075008000000	-0.011613000000
H	-1.544552000000	-0.304122000000	-1.036093000000
C	-1.492620000000	1.595573000000	-0.064783000000
H	-2.481314000000	1.964722000000	-0.339673000000
H	-1.268971000000	1.974952000000	0.938480000000
C	-2.760509000000	-0.460598000000	0.754453000000
H	-2.562600000000	-1.504578000000	0.998997000000
H	-2.844372000000	0.074923000000	1.703483000000
C	-4.063950000000	-0.365044000000	-0.030313000000
H	-3.990240000000	-0.905389000000	-0.975152000000
H	-4.331897000000	0.666780000000	-0.257538000000
H	-4.885621000000	-0.799074000000	0.537671000000
O	-0.584856000000	2.079077000000	-1.018356000000

H	0.293561000000	2.145714000000	-0.622840000000

R-BuOH-O_4-*R*-BuOH ((2*R*,2'*R*)-2,2'-tetraoxidanediylbis(butan-1-ol))

E(ωB97X-D/aug-cc-pVTZ) = -766.8372686 Eh
E(DLPNO-CCSD(T)-F12/cc-pVTZ-F12) = -765.9089194 Eh
0 1

H	2.005530000000	2.270441000000	0.857324000000
C	1.451255000000	1.491118000000	0.335749000000
H	0.725889000000	1.070975000000	1.039632000000
C	2.422964000000	0.410733000000	-0.104879000000
H	3.147494000000	0.844244000000	-0.799955000000
C	3.147551000000	-0.231017000000	1.070637000000
H	2.411236000000	-0.672386000000	1.745363000000
H	3.638332000000	0.568832000000	1.629721000000
C	4.170598000000	-1.277208000000	0.650964000000
H	4.672767000000	-1.696555000000	1.521799000000
H	4.931677000000	-0.842349000000	0.001335000000
H	3.695400000000	-2.092618000000	0.107930000000
O	0.793867000000	2.111481000000	-0.750107000000
H	0.392653000000	1.407461000000	-1.272409000000
O	1.800423000000	-0.551547000000	-0.963130000000
O	0.778451000000	-1.239246000000	-0.220805000000
O	-0.298855000000	-1.331344000000	-1.056036000000
O	-1.026957000000	-0.116760000000	-1.019640000000
C	-1.979067000000	-0.138663000000	0.062029000000
H	-1.436398000000	-0.348995000000	0.987965000000
C	-2.525642000000	1.283794000000	0.117921000000
H	-3.430646000000	1.268411000000	0.724649000000
H	-2.820507000000	1.577189000000	-0.897433000000
C	-3.064267000000	-1.176544000000	-0.186530000000
H	-2.588960000000	-2.087094000000	-0.549518000000
H	-3.717592000000	-0.820787000000	-0.987220000000
C	-3.875521000000	-1.497342000000	1.063523000000
H	-4.622959000000	-2.259770000000	0.848984000000
H	-4.400507000000	-0.623499000000	1.449698000000
H	-3.231846000000	-1.876409000000	1.858561000000
O	-1.665313000000	2.220061000000	0.704296000000
H	-0.911732000000	2.382524000000	0.120466000000

R-BuOH-O_4-*S*-BuOH ((2*R*,2'S)-2,2'-tetraoxidanediylbis(butan-1-ol))

E(ωB97X-D/aug-cc-pVTZ) = -766.8393386 Eh
E(DLPNO-CCSD(T)-F12/cc-pVTZ-F12) = -765.9100069 Eh
0 1

H	3.529761000000	0.118670000000	1.649430000000
C	3.226170000000	-0.513401000000	0.811732000000
H	4.050724000000	-0.506963000000	0.094635000000
C	2.009356000000	0.126068000000	0.164165000000
H	1.158858000000	0.117468000000	0.846945000000
C	2.267313000000	1.549873000000	-0.297092000000
H	2.666054000000	2.128642000000	0.535311000000

O	1.629413000000	-0.612575000000	-1.018462000000
O	0.503169000000	-1.419406000000	-0.735796000000
O	-0.532637000000	-0.991502000000	-1.546384000000
O	-1.044278000000	0.221387000000	-1.039528000000
C	-1.889254000000	-0.013807000000	0.099817000000
H	-1.355142000000	-0.679080000000	0.783094000000
C	-2.014801000000	1.354384000000	0.757356000000
H	-2.391025000000	2.067789000000	0.013883000000
H	-2.762698000000	1.282741000000	1.546877000000
C	-3.219373000000	-0.617059000000	-0.323375000000
H	-3.012401000000	-1.420455000000	-1.030641000000
H	-3.796065000000	0.137865000000	-0.863444000000
C	-4.025388000000	-1.169209000000	0.846892000000
H	-3.457265000000	-1.926712000000	1.388839000000
H	-4.304128000000	-0.390680000000	1.557059000000
H	-4.944810000000	-1.633573000000	0.493203000000
O	-0.827385000000	1.802122000000	1.352226000000
H	-0.207161000000	2.069629000000	0.657722000000
H	3.016321000000	1.544383000000	-1.096134000000
O	1.090261000000	2.205699000000	-0.722825000000
H	0.624069000000	1.618134000000	-1.326458000000
C	2.967773000000	-1.931807000000	1.300234000000
H	2.685021000000	-2.584175000000	0.474827000000
H	2.161821000000	-1.950881000000	2.035259000000
H	3.858950000000	-2.348851000000	1.767371000000

R-PrNO3-O4-*R*-PrNO3 ((2*R*,2’*R*)-tetraoxidanediylbis(propane-2,1-diyl) dinitrate)

E(ωB97X-D/aug-cc-pVTZ) = -1097.1558541 Eh

E(DLPNO-CCSD(T)-F12/cc-pVTZ-F12) = -1095.935697 Eh

0 1

C	-2.326445000000	-1.222577000000	0.700369000000
H	-2.489212000000	-2.248895000000	0.382594000000
H	-3.158701000000	-0.896799000000	1.322332000000
C	-2.175436000000	-0.310925000000	-0.501966000000
H	-1.207225000000	-0.489397000000	-0.974344000000
C	-3.307636000000	-0.504428000000	-1.490028000000
H	-3.250902000000	-1.497504000000	-1.934942000000
H	-3.233976000000	0.230135000000	-2.288493000000
H	-4.275239000000	-0.389207000000	-1.000442000000
O	-2.172353000000	1.003711000000	0.066787000000
O	-1.815525000000	1.956894000000	-0.913861000000
O	-0.456341000000	1.878773000000	-1.147110000000
O	0.218415000000	2.606764000000	-0.143796000000
C	0.791620000000	1.711289000000	0.818752000000
H	0.018500000000	1.038729000000	1.185679000000
C	1.274623000000	2.615369000000	1.937232000000
H	0.446646000000	3.212376000000	2.311808000000
H	2.058262000000	3.287280000000	1.586925000000
H	1.667432000000	2.012378000000	2.753989000000
C	1.952608000000	0.919378000000	0.246958000000

H	2.620849000000	1.570706000000	-0.315440000000
H	2.502996000000	0.434332000000	1.052071000000
O	-1.205179000000	-1.148018000000	1.587970000000
N	-0.127327000000	-1.966443000000	1.253073000000
O	-0.268783000000	-2.751591000000	0.362536000000
O	0.826458000000	-1.765994000000	1.942745000000
O	1.408226000000	-0.085499000000	-0.620581000000
N	2.360679000000	-0.890606000000	-1.236485000000
O	3.512274000000	-0.688335000000	-0.972065000000
O	1.879195000000	-1.695884000000	-1.970845000000

R-PrNO3-O4-*S*-PrNO3 ((2*R*,2'*S*)-tetraoxidanediylbis(propane-2,1-diyl) dinitrate)

E(ωB97X-D/aug-cc-pVTZ) = -1097.1554367 Eh

E(DLPNO-CCSD(T)-F12/cc-pVTZ-F12) = -1095.935158 Eh

0 1

C	-2.205237000000	0.589243000000	-0.034450000000
H	-3.252947000000	0.758280000000	-0.279126000000
H	-2.120434000000	0.291977000000	1.010751000000
C	-1.385473000000	1.831392000000	-0.311128000000
H	-1.221286000000	1.918407000000	-1.386499000000
C	-2.037098000000	3.080837000000	0.246690000000
H	-2.973242000000	3.288512000000	-0.271161000000
H	-1.373697000000	3.932313000000	0.110084000000
H	-2.239117000000	2.966322000000	1.311571000000
O	-0.121409000000	1.585237000000	0.317071000000
O	0.867563000000	2.393356000000	-0.278040000000
O	1.378058000000	1.718902000000	-1.380507000000
O	2.353891000000	0.820251000000	-0.925164000000
C	1.813139000000	-0.514212000000	-0.875022000000
H	0.734647000000	-0.445205000000	-0.785858000000
C	2.394521000000	-1.153981000000	0.371006000000
H	3.479887000000	-1.066496000000	0.374356000000
H	2.115078000000	-2.204268000000	0.420886000000
C	2.188980000000	-1.291219000000	-2.121254000000
H	3.271695000000	-1.382128000000	-2.215779000000
H	1.748809000000	-2.287737000000	-2.092144000000
O	-1.664201000000	-0.432409000000	-0.880079000000
N	-2.172214000000	-1.705791000000	-0.651032000000
O	-3.076960000000	-1.809269000000	0.126045000000
O	-1.622303000000	-2.543626000000	-1.297035000000
H	1.807552000000	-0.777954000000	-3.001703000000
O	2.027115000000	-0.483136000000	1.581861000000
N	0.742958000000	-0.733881000000	2.029429000000
O	0.457974000000	-0.116793000000	3.006224000000
O	0.077129000000	-1.528349000000	1.417223000000

S10. CASSCF / 6-311++G(d,p) Optimized Geometries

HO_4H (tetraoxidane)

HO_2
E(CASSCF(5,4)/6-311++G(d,p)) = -150.2702347 Eh
0 2

O	0.053014744200	0.726831425400	0.000000000000
O	0.048757498300	-0.625914065700	0.000000000000
H	-0.871794242500	-0.855782359600	0.000000000000

HO_2 + HO_2
E(CASSCF(10,8)/6-311++G(d,p)) = -300.5404556 Eh
E(XMCQDPT2(10,8)/6-311++G(d,p)) = -301.2157142 Eh
E(ASCI-SCF(PT2)(26,18)/6-311++G(d,p), 5M det) = -300.611942 Eh
E(ASCI-SCF(PT2)(26,18)/cc-pVDZ, 5M det) = -300.543957 Eh
E(ASCI-SCF(PT2)(26,18)/cc-pVTZ, 5M det) = -300.638829 Eh
0 1

H	5.312899465100	0.031572971900	3.005664928200
O	6.040358023400	-0.065139881900	3.607124522900
O	5.473294249000	-0.536285372000	4.741326160000
O	-6.417908163000	0.777759411000	-4.307021286000
O	-5.334540508200	0.665649696000	-3.504770993900
H	-5.684319580400	0.334771314800	-2.687271032200

HO_2…HO_2
E(CASSCF(10,8)/6-311++G(d,p)) = -300.5472134 Eh
E(XMCQDPT2(10,8)/6-311++G(d,p)) = -301.2244265 Eh
E(ASCI-SCF(PT2)(26,18)/6-311++G(d,p), 5M det) = -300.612281 Eh
E(ASCI-SCF(PT2)(26,18)/cc-pVDZ, 5M det) = -300.551332 Eh
E(ASCI-SCF(PT2)(26,18)/cc-pVTZ, 5M det) = -300.644111 Eh
0 1

H	1.043628553800	0.501342847500	-0.237042159600
O	1.774662923300	0.405197783400	0.364294710400
O	1.206212469200	-0.064748827900	1.494384623600
O	-2.150826314800	0.306222848800	-1.060079781700
O	-1.072597491100	0.191748393100	-0.253198844200
H	-1.405292141300	-0.139335044900	0.572478452500

$[HOO\cdots OOH]^{\ddagger}$
E(CASSCF(10,8)/6-311++G(d,p)) = -300.5400247 Eh
E(XMCQDPT2(10,8)/6-311++G(d,p)) = -301.2225510 Eh
E(ASCI-SCF(PT2)(26,18)/6-311++G(d,p), 5M det) = -300.614866 Eh
E(ASCI-SCF(PT2)(26,18)/cc-pVDZ, 5M det) = -300.547074 Eh
E(ASCI-SCF(PT2)(26,18)/cc-pVTZ, 5M det) = -300.639925 Eh
$\tilde{\nu}^{\ddagger}$ = -135.65 cm^{-1}
0 1

H	1.244027468113	1.209253657573	0.388166125107
O	1.577955089321	0.438885744765	-0.051896721743
O	0.849792210567	-0.585152494374	0.491993883621

O	-0.822490805595	-0.621396477001	-0.501496993335
O	-1.474867302849	0.524291655980	-0.097290463068
H	-1.978628703314	0.234545999991	0.651361234189

HO_4H

E(CASSCF(10,8)/6-311++G(d,p)) = -300.5467084 Eh
E(XMCQDPT2(10,8)/6-311++G(d,p)) = -301.2291254 Eh
E(ASCI-SCF(PT2)(26,18)/6-311++G(d,p), 5M det) = -300.626685 Eh
E(ASCI-SCF(PT2)(26,18)/cc-pVDZ, 5M det) = -300.554484 Eh
E(ASCI-SCF(PT2)(26,18)/cc-pVTZ, 5M det) = -300.652228 Eh
0 1

H	1.206143508358	1.218380974234	0.214166705515
O	1.507679337226	0.422153503588	-0.202116753650
O	0.581080746064	-0.562355312740	0.405646567403
O	-0.554141185139	-0.578369348893	-0.365926700527
O	-1.397594641214	0.571423534378	0.098694985160
H	-1.947379809052	0.129194737372	0.730372259916

$[HO{\cdots}O_2{\cdots}OH]^‡$

E(CASSCF(10,8)/6-311++G(d,p)) = -300.5429746 Eh
E(XMCQDPT2(10,8)/6-311++G(d,p)) = -301.2263951 Eh
E(ASCI-SCF(PT2)(26,18)/6-311++G(d,p), 5M det) = -300.612060 Eh
E(ASCI-SCF(PT2)(26,18)/cc-pVDZ, 5M det) = -300.545010 Eh
E(ASCI-SCF(PT2)(26,18)/cc-pVTZ, 5M det) = -300.639133 Eh
$\tilde{\nu}^‡$ = -337.49 cm^{-1}
0 1

H	1.378201409803	1.157058487799	0.109487371953
O	1.626779974811	0.375063687161	-0.367658469640
O	0.578477941885	-0.652969156300	0.411573255806
O	-0.512947988152	-0.696802524471	-0.236685572143
O	-1.507276383198	0.611619642311	0.313912920754
H	-2.187148305405	0.060123633366	0.679326747189

$HO{\ldots}O_2{\ldots}HO$

E(CASSCF(10,8)/6-311++G(d,p)) = -300.5665600 Eh
E(XMCQDPT2(10,8)/6-311++G(d,p)) = -301.2138756 Eh
E(ASCI-SCF(PT2)(26,18)/6-311++G(d,p), 5M det) = -300.762588 Eh
E(ASCI-SCF(PT2)(26,18)/cc-pVDZ, 5M det) = -300.548878 Eh
E(ASCI-SCF(PT2)(26,18)/cc-pVTZ, 5M det) = -300.639870 Eh
0 1

H	0.718381504400	1.207575923200	-0.063164297400
O	1.573430678400	1.476653795100	-0.387308241000
O	0.705666613600	-2.181953008600	0.386858181100
O	-0.282286442500	-2.693371745000	-0.102325351800
O	-1.323774322400	0.831061136600	0.581653084400
H	-2.092345205700	1.391748807600	0.613144807600

MeO_4H (1-methyltetraoxidane)

MeO_2

E(CASSCF(5,4)/6-311++G(d,p)) = -189.3072503

0 2

C	0.986112137800	-0.476193762300	-0.000001179200
O	0.022290135100	0.565387927500	0.000000161200
O	-1.218628941300	0.054997161400	-0.000000033400
H	1.946367123200	0.019792583800	0.000000070900
H	0.870568201200	-1.080029183900	0.889297526600
H	0.870568344000	-1.080028726600	-0.889296546200

HO_2

-follow-this-link-

MeO_2 + HO_2

E(CASSCF(10,8)/6-311++G(d,p)) = -339.5774526 Eh
E(XMCQDPT2(10,8)/6-311++G(d,p)) = -340.4091666 Eh
E(ASCI-SCF(PT2)(32,24)/6-311++G(d,p), 5M det) = -339.729540 Eh
E(ASCI-SCF(PT2)(32,24)/cc-pVDZ, 5M det) = -339.643700 Eh
E(ASCI-SCF(PT2)(32,24)/cc-pVTZ, 5M det) = -339.750560 Eh

0 1

H	4.396114690500	-5.347771793600	-3.150875254300
C	3.342285676000	-5.121084990600	-3.229103823500
H	2.939822514000	-4.847871990700	-2.263573886300
H	3.175846237500	-4.332080994000	-3.949226431700
O	2.730176826700	-6.318775037500	-3.681312843200
O	1.406255000200	-6.146377884100	-3.814598263000
O	-2.361537074000	6.149813482000	3.874680183000
O	-3.165914823000	5.620625396000	2.924524289400
H	-2.692793362200	4.857649546100	2.617514940200

MeO_2…HO_2

E(CASSCF(10,8)/6-311++G(d,p)) = -339.5843401 Eh
E(XMCQDPT2(10,8)/6-311++G(d,p)) = -340.4200416 Eh
E(ASCI-SCF(PT2)(32,24)/6-311++G(d,p), 5M det) = -339.753318 Eh
E(ASCI-SCF(PT2)(32,24)/cc-pVDZ, 5M det) = -339.652551 Eh
E(ASCI-SCF(PT2)(32,24)/cc-pVTZ, 5M det) = -339.757384 Eh

0 1

H	2.949487651642	-0.638970446296	-0.197732202318
C	1.900222325619	-0.399029813929	-0.286063297731
H	1.482426115390	-0.132316025610	0.673090697745
H	1.750961729848	0.393849626506	-1.005535475823
O	1.285683739116	-1.593659424436	-0.762260898197
O	-0.036264544603	-1.450369566045	-0.870464339032
O	-0.918817320550	1.434265486891	0.930546259420
O	-1.723195068799	0.905077400553	-0.016179198193
H	-1.243986498100	0.143644200398	-0.328301941789

[MeOO···OOH]‡

E(CASSCF(10,8)/6-311++G(d,p)) = -339.5762123 Eh
E(XMCQDPT2(10,8)/6-311++G(d,p)) = -340.4191086 Eh
E(ASCI-SCF(PT2)(32,24)/6-311++G(d,p), 5M det) = Eh
E(ASCI-SCF(PT2)(32,24)/cc-pVDZ, 5M det) = -339.647461 Eh
E(ASCI-SCF(PT2)(32,24)/cc-pVTZ, 5M det) = -339.722862 Eh
$\tilde{\nu}^{\ddagger}$ = -163.96 cm^{-1}
0 1

C	2.024960513300	0.121662344400	0.200449568500
H	1.656354219300	0.095722872700	1.217238345200
H	2.642422306000	0.995755397200	0.044227223600
H	2.582696188900	-0.777381633000	-0.024002952500
O	0.944321934800	0.261449687300	-0.703068841200
O	0.126712874400	-0.836120515400	-0.608240623800
O	-1.138374306200	-0.372842483800	0.724319362600
O	-1.979950577700	0.531730084600	0.119804607000
H	-1.513589089800	1.353005957100	0.196943416600

MeO$_4$H

E(CASSCF(10,8)/6-311++G(d,p)) = -339.5823844 Eh
E(XMCQDPT2(10,8)/6-311++G(d,p)) = -340.4265122 Eh
E(ASCI-SCF(PT2)(32,24)/6-311++G(d,p), 5M det) = -339.756758 Eh
E(ASCI-SCF(PT2)(32,24)/cc-pVDZ, 5M det) = -339.654612 Eh
E(ASCI-SCF(PT2)(32,24)/cc-pVTZ, 5M det) = -339.794613 Eh
0 1

C	1.958711838897	0.159106796550	0.384220247850
H	1.637498630596	0.097545254081	1.417294107657
H	2.622591101965	1.005073890807	0.256609755610
H	2.465149971559	-0.751691940464	0.089843785515
O	0.870717137058	0.423502071695	-0.467396100831
O	-0.069003276977	-0.738351871506	-0.341493606745
O	-1.005601990863	-0.371790425938	0.594223162028
O	-2.031309087137	0.439883102854	-0.108886360903
H	-1.609140974553	1.288216235323	-0.101278866331

[MeO···O$_2$···OH]‡

E(CASSCF(10,8)/6-311++G(d,p)) = -339.5804486 Eh
E(XMCQDPT2(10,8)/6-311++G(d,p)) = -340.4248784 Eh
E(ASCI-SCF(PT2)(32,24)/6-311++G(d,p), 5M det) = -339.752796 Eh
E(ASCI-SCF(PT2)(32,24)/cc-pVDZ, 5M det) = -339.647588 Eh
E(ASCI-SCF(PT2)(32,24)/cc-pVTZ, 5M det) = -339.753431 Eh
$\tilde{\nu}^{\ddagger}$ = -306.69 cm^{-1}
0 1

C	2.107499827631	0.164294362874	0.368944239701
H	2.438207953612	-0.491078346548	1.167369597567
H	2.802339917959	0.992597259887	0.279513674253
H	2.067481759743	-0.376099624229	-0.570203797277
O	0.877040523503	0.752104984474	0.671632262651
O	-0.168205448208	-0.598733426348	0.817076831208

O	-1.360032511503	-0.135457312831	0.814077400955
O	-1.849326491947	0.036454557654	-0.731768068999
H	-1.604719474214	0.948438713725	-0.821996930554

MeO…O_2…HO
E(CASSCF(10,8)/6-311++G(d,p)) = -339.6088914 Eh
E(XMCQDPT2(10,8)/6-311++G(d,p)) = -340.4063968 Eh
E(ASCI-SCF(PT2)(32,24)/6-311++G(d,p), 5M det) = -339.744086 Eh
E(ASCI-SCF(PT2)(32,24)/cc-pVDZ, 5M det) = -339.654493 Eh
E(ASCI-SCF(PT2)(32,24)/cc-pVTZ, 5M det) = -339.745799 Eh
0 1

H	3.324995841700	0.236411109400	0.482348590200
C	2.512015135800	-0.338112860500	0.045346141100
H	2.314917938000	0.034424808200	-0.954269109500
H	2.798896109100	-1.383736164100	0.019684675200
O	1.420655751000	-0.148360274300	0.879724713300
O	-0.838644771900	-1.554470020200	-1.141141052800
O	-1.887050620700	-1.908135866200	-0.639164208100
O	-0.920696558900	1.820889844500	0.819195236600
H	-0.201353771000	1.199053452200	0.898015650100

MeO_4Me (1,4-dimethyltetraoxidane)
MeO_2
-follow-this-link-

MeO_2 + MeO_2
E(CASSCF(10,8)/6-311++G(d,p)) = -378.6145396 Eh
E(XMCQDPT2(10,8)/6-311++G(d,p)) = -379.5883671 Eh
E(ASCI-SCF(PT2)(38,30)/6-311++G(d,p), 5M det) = -378.844947 Eh
E(ASCI-SCF(PT2)(38,30)/cc-pVDZ, 5M det) = -378.743420 Eh
E(ASCI-SCF(PT2)(38,30)/cc-pVTZ, 5M det) = -378.862456 Eh
0 1

H	6.277637103400	1.389153794700	-3.667475017700
C	6.658028560600	1.992846227700	-4.479660141300
O	5.592171863200	2.709015714400	-5.083821879200
O	4.682081604000	1.859793645000	-5.584720742000
O	-4.653980643000	-1.722056263000	5.595990011000
O	-5.527241479400	-0.711122155000	5.721576300900
C	-6.610550369000	-0.877637521700	4.820096838700
H	-6.242140470300	-0.880279905400	3.803703334100
H	7.336807092200	2.746701485800	-4.106777925600
H	-7.257589496000	-0.028223953100	4.987185792200
H	-7.129933064900	-1.799856816400	5.040496605300
H	7.146889687000	1.373955610400	-5.219126359900

MeO_2…MeO_2
E(CASSCF(10,8)/6-311++G(d,p)) = -378.6179702 Eh
E(XMCQDPT2(10,8)/6-311++G(d,p)) = -379.5951858 Eh
E(ASCI-SCF(PT2)(38,30)/6-311++G(d,p), 5M det) = -378.873556 Eh

E(ASCI-SCF(PT2)(38,30)/cc-pVDZ, 5M det) = -378.746992 Eh
E(ASCI-SCF(PT2)(38,30)/cc-pVTZ, 5M det) = -378.864577 Eh
0 1

H	1.553185281700	-0.000534134100	1.157224646300
C	2.112424605200	0.834279822100	0.761758226600
O	1.456446510100	1.343033874100	-0.392919743800
O	1.364360201500	0.395061836200	-1.336683552600
O	-1.346601683500	-0.467161888600	1.270601627700
O	-1.403272381300	0.864068125300	1.120786146100
C	-2.062410006700	1.201034850700	-0.093464788300
H	-1.522844895700	0.777710874900	-0.927562230800
H	2.122316927300	1.651517111700	1.468462096800
H	-2.043230351600	2.280353434600	-0.139321447200
H	-3.080153205000	0.837438854700	-0.063831314900
H	3.120088928000	0.539833875600	0.503272932700

[MeOO···OOMe]‡

E(CASSCF(10,8)/6-311++G(d,p)) = -378.6137116 Eh
E(XMCQDPT2(10,8)/6-311++G(d,p)) = -379.5990650 Eh
E(ASCI-SCF(PT2)(38,30)/6-311++G(d,p), 5M det) = -378.875608 Eh
E(ASCI-SCF(PT2)(38,30)/cc-pVDZ, 5M det) = -378.750238 Eh
E(ASCI-SCF(PT2)(38,30)/cc-pVTZ, 5M det) = -378.895759 Eh
$\tilde{\nu}^{\ddagger}$ = -181.16 cm^{-1}
0 1

H	1.874642793776	0.174925393680	1.583397542704
C	2.337424242295	0.639281720314	0.723180719406
O	1.345506598452	1.134940313232	-0.151956828998
O	0.608696406067	0.076973353586	-0.651574194217
O	-0.587939697562	-0.299235079698	0.541588873247
O	-1.278999209625	0.872152325184	0.790777699270
C	-2.277640545991	1.041972701492	-0.193766707032
H	-1.821718957937	1.174022051032	-1.165604180454
H	2.904452112355	1.508919526288	1.026533197365
H	-2.808355888406	1.938693365410	0.096126572952
H	-2.948203727562	0.192426745940	-0.198530796405
H	2.976058259552	-0.065109419696	0.206079181931

MeO_4Me

E(CASSCF(10,8)/6-311++G(d,p)) = -378.6159499 Eh
E(XMCQDPT2(10,8)/6-311++G(d,p)) = -379.5942896 Eh
E(ASCI-SCF(PT2)(38,30)/6-311++G(d,p), 5M det) = -378.859063 Eh
E(ASCI-SCF(PT2)(38,30)/cc-pVDZ, 5M det) = -378.753981 Eh
E(ASCI-SCF(PT2)(38,30)/cc-pVTZ, 5M det) = -378.905721 Eh
0 1

H	2.097081252918	-0.216684023702	1.413561713415
C	2.469857606874	0.387138647043	0.595550504148
O	1.437615859135	1.156891758812	0.029464120109
O	0.462520600490	0.240927567463	-0.531445638505
O	-0.434419490477	-0.103190139459	0.542714773305
O	-1.369619812207	0.999376320384	0.661752729950

C	-2.421648118382	0.766062656464	-0.242291800540
H	-2.060700346259	0.767071613536	-1.263281750491
H	3.179290650291	1.113487472637	0.970906345306
H	-3.100214880574	1.596231417640	-0.093510211781
H	-2.923787764783	-0.166989830117	-0.016561672216
H	2.941078271025	-0.236546475151	-0.154550384217

[MeO···O_2···OMe]‡

E(CASSCF(10,8)/6-311++G(d,p)) = -378.6122696 Eh
E(XMCQDPT2(10,8)/6-311++G(d,p)) = -379.5997430 Eh
E(ASCI-SCF(PT2)(38,30)/6-311++G(d,p), 5M det) = -378.862612 Eh
E(ASCI-SCF(PT2)(38,30)/cc-pVDZ, 5M det) = -378.745198 Eh
E(ASCI-SCF(PT2)(38,30)/cc-pVTZ, 5M det) = -378.883191 Eh
$\tilde{\nu}^{‡}$ = -359.7 cm^{-1}
0 1

H	2.156234533800	-0.269037722400	1.481612060900
C	2.584364432500	0.321171546900	0.678577203100
O	1.647342393200	1.206658483400	0.139456734100
O	0.419247376400	0.211558101600	-0.469685073800
O	-0.391234840500	-0.088724675800	0.475604868200
O	-1.574250233200	1.118242966000	0.604147362400
C	-2.537580352300	0.768974096600	-0.346341519000
H	-2.124538013100	0.771654599000	-1.349610017100
H	3.364116251800	0.956176486300	1.086129106100
H	-3.291884384400	1.544893431300	-0.274598527300
H	-2.988480432100	-0.192143932900	-0.123345986100
H	3.008621267800	-0.324393380000	-0.082877211600

MeO...O_2...MeO

E(CASSCF(10,8)/6-311++G(d,p)) = -378.6436981 Eh
E(XMCQDPT2(10,8)/6-311++G(d,p)) = -379.5879436 Eh
E(ASCI-SCF(PT2)(38,30)/6-311++G(d,p), 5M det) = -378.806444 Eh
E(ASCI-SCF(PT2)(38,30)/cc-pVDZ, 5M det) = -378.716902 Eh
E(ASCI-SCF(PT2)(38,30)/cc-pVTZ, 5M det) = -378.834358 Eh
0 1

H	2.584217987700	0.252728599600	1.433973226100
C	3.458493543500	0.540596426500	0.857775013700
O	3.328628590700	1.821718799500	0.355097670000
O	-0.057877398600	-1.499513434900	-1.162225881400
O	0.016552686400	-1.885607277800	-0.011994187400
O	-3.232900744800	1.800282678400	0.824471228600
C	-3.401376746600	1.083383646000	-0.345328196900
H	-2.532248369300	1.173043520400	-0.990181830800
H	4.317980181900	0.562517773100	1.523965482200
H	-4.253820903400	1.528750299400	-0.853292405500
H	-3.623553856500	0.039384532300	-0.144424536900
H	3.650805663900	-0.179132511500	0.067415991400

EtO_4Et (1,4-diethyltetraoxidane)

EtO_2

E(CASSCF(5,4)/6-311++G(d,p)) = -228.3568920 Eh

0 2

C	0.479368657800	0.630449186800	0.251375900700
H	0.298133957000	0.700641777400	1.315506636600
H	0.810764312700	1.592355055500	-0.117848104300
O	-0.777761261700	0.433641635000	-0.397539491100
O	-1.470074279200	-0.553675317500	0.190736922900
C	1.464235608600	-0.468513004200	-0.089427740800
H	1.109470887500	-1.428181272000	0.267287011200
H	2.419021248200	-0.257765299600	0.383034482100
H	1.617194869000	-0.528552761400	-1.161734617500

EtO_2 + EtO_2

E(CASSCF(10,8)/6-311++G(d,p)) = -456.7146139 Eh

E(XMCQDPT2(10,8)/6-311++G(d,p)) = -458.0012221 Eh

0 1

H	-2.944301492300	1.401189389800	4.758132041800
C	-3.717215668700	0.999228161300	5.404009867300
H	-4.616160179600	0.853097736500	4.814003938400
H	-3.930793861600	1.724729070900	6.181202160300
C	-3.287486621300	-0.322678547700	6.001199617600
H	-3.053059863300	-1.055184210000	5.239772985400
H	-4.035565604400	-0.729381432600	6.669277298200
O	-2.102398892000	-0.078242610900	6.759925046300
O	-1.652705173400	-1.211478903300	7.317752095000
O	1.646656333000	-1.200438038000	-7.315373240000
O	2.108913631000	-0.064762270000	-6.771699622600
C	3.287466478400	-0.313946783500	-6.004088472400
H	3.044005993400	-1.043675919700	-5.242954645500
H	4.038059388200	-0.726064510600	-6.666047628300
C	3.719782581700	1.007105448600	-5.406835664600
H	3.935932639400	1.731575268400	-6.184344292100
H	4.617804482300	0.859501065700	-4.815759178600
H	2.947004061400	1.411150073900	-4.762201331200

$EtO_2 \ldots EtO_2$

E(CASSCF(10,8)/6-311++G(d,p)) = -456.7184737 Eh

E(XMCQDPT2(10,8)/6-311++G(d,p)) = -458.0097144 Eh

0 1

H	-1.735578461704	1.397218775186	-0.603095054674
C	-2.508842070731	1.004376575742	0.046634225402
H	-3.408796036903	0.856643978060	-0.541526575220
H	-2.721896620138	1.732569571508	0.822043172549
C	-2.083152266885	-0.317295064006	0.646319334804
H	-1.837265288081	-1.045961613772	-0.113355772210
H	-2.833568077257	-0.725766524559	1.310762550957
O	-0.902446477377	-0.072156453223	1.418642307775
O	-0.443397140095	-1.208168492521	1.960394601011

O	0.439514904817	-1.204902913259	-1.958015745808
O	0.901772236292	-0.069227063019	-1.418312513718
C	2.081753447789	-0.316353842892	-0.645525715777
H	1.833803105817	-1.043045586563	0.115359617329
H	2.831045806076	-0.728029301716	-1.309263050809
C	2.511135859897	1.005109158777	-0.048011896459
H	2.726497750488	1.731419324428	-0.824550203742
H	3.410515895030	0.855724514968	0.540593449142
H	1.738902431972	1.401318662496	0.600911268545

[EtOO···OOEt]‡

E(CASSCF(10,8)/6-311++G(d,p)) = -456.7132710 Eh
E(XMCQDPT2(10,8)/6-311++G(d,p)) = -458.0187675 Eh
$\tilde{\nu}^{\ddagger}$ = -175.2 cm^{-1}
0 1

H	-2.586292226354	1.828024654409	-0.292151452188
C	-3.155417145575	1.002100197566	0.120071557713
H	-4.083803255785	0.913433704163	-0.435275699531
H	-3.394294760863	1.227101960874	1.153960064570
C	-2.378422203253	-0.292948738201	0.012634747918
H	-2.116421214278	-0.518283753528	-1.013139765379
H	-2.927129379037	-1.127552483682	0.432705206333
O	-1.180245143465	-0.116524371433	0.755183941706
O	-0.448407103475	-1.288953968364	0.737273306406
O	0.448379813487	-1.288794551337	-0.737408362388
O	1.180166973508	-0.116327286405	-0.755117138688
C	2.378331046280	-0.292827261224	-0.012562852892
H	2.116311844294	-0.518050657512	1.013232414367
H	2.926928891982	-1.127535882651	-0.432571240349
C	3.155503050569	1.002111796602	-0.120108734691
H	3.394387353864	1.227001943887	-1.154018915589
H	4.083893888819	0.913333141170	0.435218371537
H	2.586528567338	1.828164790440	0.292078551142

EtO_4Et

E(CASSCF(10,8)/6-311++G(d,p)) = -456.7157146 Eh
E(XMCQDPT2(10,8)/6-311++G(d,p)) = -458.0182863 Eh
0 1

H	-3.200708410825	1.658054216080	-0.767564337616
C	-3.561144567928	0.828531483010	-0.169373354251
H	-4.467767456619	0.443020604077	-0.625231458286
H	-3.803756471543	1.195589293609	0.821904246535
C	-2.523302650765	-0.274582523888	-0.098595064166
H	-2.270188978417	-0.635476983043	-1.089255303912
H	-2.872228447036	-1.106156652146	0.504387286549
O	-1.377442200791	0.291624354129	0.502270669365
O	-0.352357697531	-0.773643107062	0.595238753130
O	0.352415329537	-0.773677575022	-0.595557722126
O	1.377569351765	0.291520877116	-0.502592503379

C	2.523267341783	-0.274617108902	0.098646313130
H	2.269907920421	-0.635351189038	1.089307577893
H	2.872316616062	-1.106300529143	-0.504113705509
C	3.561131179955	0.828476056033	0.169456349299
H	3.803449907521	1.195797720599	-0.821792386508
H	4.467905676605	0.442802351059	0.624879619287
H	3.200932557872	1.657861948100	0.767989020615

[EtO···O_2···OEt]‡

E(CASSCF(10,8)/6-311++G(d,p)) = -456.7137871 Eh
E(XMCQDPT2(10,8)/6-311++G(d,p)) = -458.0194713 Eh
$\tilde{\nu}^‡$ = -266.36 cm^{-1}
0 1

H	-3.643554097903	0.479901896773	-1.413505238402
C	-3.101811686682	0.940840410168	-0.594858342106
H	-3.817858305545	1.416242564570	0.068239601921
H	-2.448446085339	1.706401319603	-0.999435344391
C	-2.305480879996	-0.095855541965	0.179512010022
H	-2.963111705635	-0.863617134528	0.575973415711
H	-1.763782870768	0.361341563183	1.000787890483
O	-1.398797841314	-0.668836430434	-0.727381556695
O	-0.615555829591	-1.827797264405	0.172761232514
O	0.614831504526	-1.825830251239	-0.183899539298
O	1.395907163125	-0.669268524455	0.721871538278
C	2.306566698052	-0.095500283899	-0.180474517079
H	2.964847361767	-0.863195602515	-0.575996055713
H	1.768449625077	0.363903980342	-1.002886559667
C	3.101151775611	0.938827200992	0.598850582380
H	2.447132226232	1.704337378469	1.002469853592
H	3.820196611694	1.414701984445	-0.060647915408
H	3.639314845626	0.475706086455	1.418622219730

EtO…O_2…EtO

E(CASSCF(10,8)/6-311++G(d,p)) = -456.7449842 Eh
E(XMCQDPT2(10,8)/6-311++G(d,p)) = 457.9965574 Eh
0 1

H	-3.576147853200	1.150404042500	-1.600516205300
C	-3.164897180900	1.100591127600	-0.597040970100
H	-3.976416334400	0.885444103400	0.092851306200
H	-2.750768852000	2.072265529900	-0.348927816700
C	-2.096956203800	0.024046908800	-0.504990621700
H	-2.500437035800	-0.961315693300	-0.728168669400
H	-1.669727517700	-0.028811881500	0.493827608600
O	-1.019201682700	0.246219206000	-1.352158506700
O	-0.549085766700	-3.487908531900	0.246305734300
O	0.554414584300	-3.491518824300	-0.262777642900
O	1.021340826300	0.248466957800	1.359770394000
C	2.094372529400	0.027034344000	0.506463402500
H	2.495732855600	-0.960957800200	0.721938837700

H	1.661963922000	-0.018726123400	-0.490452102700
C	3.166322990200	1.099507571000	0.599142784000
H	2.753939670100	2.073994273000	0.359246125100
H	3.973068592700	0.885851991100	-0.096820183800
H	3.583548089700	1.141903850600	1.600431876800

*i*PrO$_4$*i*Pr (1,4-diisopropyltetraoxidane)

iPrO$_2$

E(CASSCF(5,4)/6-311++G(d,p)) = -267.4063321 Eh

0 2

H	1.316126173600	1.925314635800	-0.406603778500
C	0.481078592500	1.461226242500	0.109268581800
H	0.659390661200	1.522596196600	1.178471792400
H	-0.417883795500	2.017664342100	-0.126606589400
C	0.348776325400	0.013626789100	-0.328188907100
H	0.137110747600	-0.043936779100	-1.388458107600
C	1.552094965100	-0.842137711400	0.020783339100
H	1.732367498500	-0.834818983800	1.091040541000
H	2.434068652600	-0.453789948500	-0.477782005200
H	1.400406072700	-1.867704145900	-0.296478078100
O	-0.763480621000	-0.602794249900	0.344678869100
O	-1.932561272600	-0.166472387500	-0.144050657600

iPrO$_2$ + iPrO$_2$

E(CASSCF(10,8)/6-311++G(d,p)) = -534.8127028 Eh

E(XMCQDPT2(10,8)/6-311++G(d,p)) = -536.4053348 Eh

0 1

C	6.171806456600	-0.083005377200	-5.501562468300
C	6.966356619200	-1.050244869200	-6.360462765800
C	6.930715638800	1.181206175000	-5.143855910300
O	5.005640940900	0.364133166700	-6.215795536500
O	4.080259679000	-0.601965879000	-6.294365696000
O	-4.079755486000	-0.609381862000	6.291900530000
O	-5.005166636900	0.356789882600	6.214553945200
C	-6.172019644800	-0.090038116300	5.501259214100
C	-6.965888368600	-1.057462392000	6.360600416600
C	-6.931163169600	1.174362729700	5.144608528500
H	5.814089912000	-0.576593052700	-4.606711338300
H	6.369282049300	-1.917052927700	-6.616467537300
H	7.843829847100	-1.387483753300	-5.817198593900
H	7.292736205000	-0.568020769100	-7.276802687300
H	6.315153774300	1.843561529000	-4.545713296100
H	7.817972493000	0.926360599300	-4.573688226600
H	7.239400066800	1.711491070600	-6.039105021000
H	-5.815196538700	-0.583381417300	4.605946353300
H	-7.843949991200	-1.394365882400	5.818074414000
H	-6.368702462600	-1.924444111000	6.615711892500
H	-7.291304979600	-0.575501737500	7.277431953700
H	-7.818985123100	0.919776584000	4.575284848300

H	-6.316101642800	1.836802759000	4.546072654100
H	-7.238894342100	1.704446187200	6.040343251700

*i*PrO$_2$…*i*PrO$_2$

E(CASSCF(10,8)/6-311++G(d,p)) = -534.8166072 Eh

E(XMCQDPT2(10,8)/6-311++G(d,p)) = -536.4144858 Eh

0 1

C	2.254731532323	0.105308322637	-0.079109420733
C	3.578876938231	-0.357490922908	-0.659121566739
C	2.350797640266	1.390030648663	0.721538388285
O	1.326906090118	0.390870278318	-1.146284075041
O	0.889911709456	-0.733417692129	-1.727925536154
O	-0.889497097441	-0.734344548197	1.726801001056
O	-1.326657327069	0.390114008251	1.145619029999
C	-2.254452902298	0.104852721610	0.078337485696
C	-3.578509093207	-0.358399545962	0.658181092665
C	-2.350736098275	1.389896886678	-0.721764880275
H	1.803027288608	-0.677152937057	0.515502724357
H	3.447309733724	-1.253703995827	-1.253264453796
H	4.271489998383	-0.580518985043	0.146627135614
H	4.015364523830	0.415795090108	-1.284093084006
H	1.377858050816	1.676906305439	1.101727024808
H	3.019429000704	1.243204465038	1.563325704232
H	2.742513767669	2.197297615155	0.110096888997
H	-1.802636593553	-0.677284332033	-0.516614016424
H	-4.271123303368	-0.581179990082	-0.147636771673
H	-3.446765037665	-1.254828677878	1.251959065705
H	-4.015103731841	0.414556637012	1.283482169986
H	-3.019321134696	1.243299432034	-1.563628865274
H	-1.377838160790	1.677101682495	-1.101807666817
H	-2.742608579669	2.196843054120	-0.109997884961

[*i*PrOO···OO*i*Pr]‡

E(CASSCF(10,8)/6-311++G(d,p)) = -534.8102794 Eh

E(XMCQDPT2(10,8)/6-311++G(d,p)) = -536.4223293 Eh

$\tilde{\nu}^‡$ = -167.52 cm^{-1}

0 1

C	2.396126170549	-0.061307974437	0.057562311180
C	3.356325038092	-1.010800873219	-0.638916511281
C	2.951058512771	1.341753979161	0.227876265502
O	1.201520886056	0.109910020953	-0.710604492459
O	0.462457063501	-1.061250979888	-0.715487809805
O	-0.462368777474	-1.061306954882	0.715816260863
O	-1.201563665009	0.109775405934	0.710936143504
C	-2.396021744563	-0.061472615449	-0.057467405146
C	-3.356073898084	-1.011542876244	0.638429412249
C	-2.951332739777	1.341515570152	-0.227239494453
H	2.099746694114	-0.461058914396	1.019382859813
H	2.904510506376	-1.985478759818	-0.777367106331

H	4.252862140052	-1.133959418112	-0.038277832785
H	3.643861042933	-0.619187765830	-1.610230587657
H	2.229381599482	1.979933929423	0.724761678507
H	3.855085143258	1.308653585782	0.827397674927
H	3.195327555473	1.779147152878	-0.735408720258
H	-2.099384871075	-0.460716069346	-1.019419804852
H	-4.252480037036	-1.134660526213	0.037577483735
H	-2.904020316367	-1.986158244838	0.776518347246
H	-3.643887879945	-0.620439296959	1.609861756618
H	-3.855183652249	1.308447723767	-0.827036774879
H	-2.229712830519	1.980156648432	-0.723591306393
H	-3.196006701502	1.778362301789	0.736198543338

*i*PrO_4*i*Pr

E(CASSCF(10,8)/6-311++G(d,p)) = -534.8119615 Eh
E(XMCQDPT2(10,8)/6-311++G(d,p)) = -536.4164529 Eh
0 1

C	2.506917072598	-0.038056460743	0.212630634403
C	3.271172980930	-1.025007036262	-0.655295428482
C	3.313987116042	1.201821411054	0.561328484664
O	1.358490300401	0.464361750647	-0.461841502458
O	0.392824025434	-0.605317137836	-0.606747977924
O	-0.392319831436	-0.606030691921	0.604282811795
O	-1.358015187339	0.463795962588	0.460654556354
C	-2.507117056618	-0.038313493775	-0.212892870412
C	-3.270625913891	-1.025421539266	0.655511264483
C	-3.314452080066	1.201733790044	-0.560394186565
H	2.171067927253	-0.519723272652	1.124419165446
H	2.651979215076	-1.874951365814	-0.916256554371
H	4.141900381010	-1.392359091848	-0.119638171654
H	3.606129450188	-0.546683803601	-1.570873288795
H	2.733018040983	1.873642736724	1.182591944656
H	4.210289294949	0.915909094366	1.102445685878
H	3.611249953562	1.732245090497	-0.338027130473
H	-2.172228138317	-0.519721959658	-1.125174974494
H	-4.141524573986	-1.393097564891	0.120355081729
H	-2.651063543010	-1.875147460804	0.916331444382
H	-3.605255036114	-0.547006594632	1.571154589789
H	-4.211123252046	0.916095639348	-1.101041267768
H	-2.733910596046	1.873895620742	-1.181693672573
H	-3.611165311555	1.731651428449	0.339442251563

[*i*PrO···O_2···O*i*Pr]‡

E(CASSCF(10,8)/6-311++G(d,p)) = -534.8072310 Eh
E(XMCQDPT2(10,8)/6-311++G(d,p)) = -536.4250636 Eh
$\tilde{\nu}^‡$ = -336.07 cm^{-1}
0 1

C	2.614437828373	0.002521294161	0.259759389809
C	3.329723220207	-1.081687083341	-0.533125592593

C	3.521415698053	1.177521906270	0.607869178041
O	1.554605390599	0.575581357707	-0.473352446284
O	0.329605271857	-0.606152255895	-0.553728981093
O	-0.328134778771	-0.608246871053	0.542905398349
O	-1.551964055437	0.574575493623	0.467241872862
C	-2.615379257453	0.002128098160	-0.261216069940
C	-3.328029428081	-1.081341477309	0.535028607751
C	-3.522997271158	1.177720930310	-0.605624200872
H	2.210874972166	-0.409732634667	1.180762124501
H	2.652114223115	-1.888033970779	-0.789873748216
H	4.145033660245	-1.498982839586	0.051334145737
H	3.737942670732	-0.671940071669	-1.452150091169
H	2.979488922801	1.921988436234	1.179549114460
H	4.361948330932	0.828516916030	1.199420498897
H	3.906839579982	1.645161378152	-0.292812725202
H	-2.216078667495	-0.410686686742	-1.183831744772
H	-4.146248522301	-1.498094804490	-0.045734579311
H	-2.649953276965	-1.888192127782	0.788948668172
H	-3.731854639308	-0.671053475617	1.455751100438
H	-4.366454178262	0.829192659079	-1.193287253458
H	-2.983078356053	1.921591053172	-1.179967743459
H	-3.904002255770	1.645931906212	0.296636902486

*i*PrO…O_2…*i*PrO

E(CASSCF(10,8)/6-311++G(d,p)) = -534.8393208 Eh
E(XMCQDPT2(10,8)/6-311++G(d,p)) = -536.3972049 Eh
0 1

C	3.380991754500	0.252024561400	-0.039517226500
C	4.164418470900	-0.973873895000	-0.488886994600
C	4.264079889600	1.324627189500	0.594131971800
O	2.758020558200	0.856223757400	-1.130767795400
O	-0.019236860700	-2.749091213000	-0.620901113700
O	0.016212616900	-2.748810669000	0.594236377600
O	-2.749861426600	0.853763861500	1.125892283700
C	-3.380414309800	0.250796164300	0.038312931000
C	-4.163593897000	-0.973474257800	0.492533032600
C	-4.265120380700	1.325106393700	-0.590266778900
H	2.616327364100	-0.039534195100	0.678371954500
H	3.511412943200	-1.682422103600	-0.988834848200
H	4.610710733300	-1.472775811300	0.366781246800
H	4.952520322000	-0.690675234000	-1.179757418900
H	3.672887140400	2.182517793700	0.894724273100
H	4.752945589900	0.918889863000	1.473637232900
H	5.026401972100	1.654733494100	-0.104407379500
H	-2.620742763800	-0.042528602000	-0.684071099400
H	-4.615835671900	-1.471455186800	-0.360508221600
H	-3.509000905000	-1.683209607000	0.988646671700
H	-4.946913211400	-0.688565453000	1.188099824700
H	-4.759938700700	0.920151850600	-1.466826923900
H	-3.674019252900	2.181707420100	-0.894540603500

H	-5.022513865700	1.656818940500	0.112790031700

AcO_4Me (1-(methyltetraoxidaneyl)ethan-1-one)

AcO_2

E(CASSCF(5,4)/6-311++G(d,p)) = -302.0834200 Eh

0 2

C	-1.448400804901	0.205714463922	-0.000000042017
H	-1.700062803135	-0.378971476462	0.875442033395
H	-1.700062853142	-0.378971629447	-0.875442003390
H	-1.995595688524	1.136343319285	0.000000003016
C	0.011193189821	0.548226958703	0.000000161981
O	0.921046876700	-0.508564043839	0.000000187011
O	0.338491696503	-1.726435857060	-0.000000212994
O	0.484282016086	1.614221301943	-0.000000124992

MeO_2

-follow-this-link-

AcO_2 + MeO_2

E(CASSCF(10,8)/6-311++G(d,p)) = -491.3919127 Eh

E(XMCQDPT2(10,8)/6-311++G(d,p)) = -492.6974999 Eh

0 1

H	-4.510259496800	-1.907139887700	-5.233882291900
C	-3.682773850200	-1.348151295600	-4.812872799200
H	-4.014859434200	-0.374816869600	-4.484529890200
H	-3.288748779800	-1.911370120200	-3.974272649200
C	-2.611211036400	-1.173455536800	-5.845498224000
O	-2.231518139400	-2.431048390800	-6.296066341100
O	-1.257769962500	-2.394087549400	-7.236030356000
O	1.785210132000	1.505046478000	6.926988855000
O	2.839932468000	2.327363639000	6.968254969100
C	3.385047261600	2.501295461700	5.669347458900
H	3.723162339400	1.548954747800	5.285385835200
H	4.218716522300	3.177518185600	5.794439919600
H	2.639790204400	2.933514882400	5.016255543600
O	-2.139089251000	-0.184816024300	-6.239228616400

AcO_2…MeO_2

E(CASSCF(10,8)/6-311++G(d,p)) = -491.3963969 Eh

E(XMCQDPT2(10,8)/6-311++G(d,p)) = -492.7031467 Eh

0 1

H	-3.393170838764	-0.485342395164	-0.032361696336
C	-2.564508368774	0.078494492692	0.380089176548
H	-2.896817158829	1.051073296131	0.710411681436
H	-2.154241981062	-0.478176388620	1.214499875945
C	-1.502175752811	0.254979519471	-0.658960006755
O	-1.145835838998	-0.998446430331	-1.138831672479
O	-0.139410652122	-0.961140186608	-2.040891959845
O	0.670267649572	0.076372138539	1.731850435449
O	1.724990062952	0.898689358121	1.768659130107

C	2.272035258577	1.075858443916	0.465807284717
H	2.613433292312	0.122869405884	0.086505070253
H	3.104624314879	1.751827227867	0.598436672372
H	1.526935361602	1.505064754038	-0.187082005559
O	-1.020186898922	1.243325820040	-1.046001973750

AcO_4Me

E(CASSCF(10,8)/6-311++G(d,p)) = -491.4038262 Eh
E(XMCQDPT2(10,8)/6-311++G(d,p)) = -492.7283605 Eh
0 1

H	-3.354779242978	-0.731082203954	0.353243309577
C	-2.797254062625	0.158004381431	0.621245479977
H	-3.379773088797	1.044755977656	0.419564566393
H	-2.560019883432	0.104381191555	1.678023560540
C	-1.516995404903	0.232847199890	-0.165170229963
O	-0.881406932839	-0.955276770183	-0.060746744392
O	0.444726210191	-0.912890475141	-0.774428987083
O	1.397850940277	-1.062114533922	0.187782643592
O	1.605123825270	0.268559224442	0.831207423189
C	2.418580155177	1.038195559175	-0.023446101693
H	3.376506036570	0.556048722275	-0.176769848813
H	2.555457231094	1.972913348884	0.505108764588
H	1.919447416039	1.224974342730	-0.965097977920
O	-1.118887334026	1.153587330584	-0.773213736981

$[AcO\cdots O_2\cdots OMe]^‡$

E(CASSCF(10,8)/6-311++G(d,p)) = -491.4020825 Eh
E(XMCQDPT2(10,8)/6-311++G(d,p)) = -492.7290640 Eh
$\tilde{\nu}^‡$ = -308.12 cm^{-1}
0 1

H	-3.407465966200	-0.866357930400	0.357508534300
C	-2.881165514100	0.048310211000	0.601693719000
H	-3.484991758600	0.909086233500	0.354293578500
H	-2.664977391900	0.043184448000	1.664337158700
C	-1.585150516800	0.134613210300	-0.163907511400
O	-0.918284600400	-1.018605560800	-0.016188137400
O	0.561683960300	-0.894215158100	-0.789739199800
O	1.470488510600	-1.118620977000	0.087038627300
O	1.804013874200	0.266476476700	0.882669573600
C	2.469690989800	1.098267602600	-0.029917699100
H	3.413212360600	0.663746017700	-0.340630381300
H	2.654660400200	2.011771962200	0.523648070500
H	1.842992822200	1.324477362800	-0.883729974900
O	-1.207955512100	1.058032473500	-0.787245094900

$AcO\ldots O_2\ldots MeO$

E(CASSCF(10,8)/6-311++G(d,p)) = -491.4362166 Eh
E(XMCQDPT2(10,8)/6-311++G(d,p)) = -492.7055704 Eh

0 1

H	-3.365006062700	-0.235524328600	1.014707614700
C	-2.431368818900	0.258808794200	0.775060141200
H	-2.606402685600	1.289785062100	0.503125004800
H	-1.779430346800	0.227651522000	1.640722598900
C	-1.728324880200	-0.423446822200	-0.365301576900
O	-1.917522394800	-1.738067598700	-0.348598593900
O	2.435897953600	-1.936900405700	-1.836528326900
O	1.671640288000	-2.733535571500	-1.328297259800
O	0.833945613000	1.157569509000	1.298295221700
C	1.766505264800	1.571335663400	0.362002750200
H	2.675129259500	0.981015244700	0.431566587100
H	1.988030451900	2.629349864000	0.465612190000
H	1.310091371400	1.396436889100	-0.608219156000
O	-1.059583185300	0.081325482200	-1.195919492200

AcO_4Ac (1,4-diacetyltetraoxidane)

AcO_2

-follow-this-link-

AcO_2 + AcO_2

E(CASSCF(10,8)/6-311++G(d,p)) = -604.1693266 Eh
E(XMCQDPT2(10,8)/6-311++G(d,p)) = -605.7944286 Eh
0 1

C	-3.264003705000	5.930281719000	-0.389459865000
C	-2.092160565000	6.466244838000	-1.153890167000
H	-1.178178278000	6.272083144000	-0.604403569000
H	-2.193476611000	7.539489583000	-1.268596463000
H	-2.056591585000	5.988596440000	-2.121388661000
O	-4.050186788000	5.141017016000	-0.727805449000
O	-3.287331052000	6.542781161000	0.856625402000
O	-4.315177271000	6.134514911000	1.637394828000
O	4.313860233000	-6.134947520000	1.635537873000
O	3.285789342000	-6.541983321000	0.854390210000
C	3.262847273000	-5.928092400000	-0.390943967000
O	4.049961095000	-5.139533771000	-0.728555533000
C	2.089569702000	-6.460916642000	-1.155382426000
H	2.188705917000	-7.534184566000	-1.271724095000
H	2.054199592000	-5.981735532000	-2.122125861000
H	1.176370823000	-6.265784862000	-0.604937779000

AcO_2…AcO_2

E(CASSCF(10,8)/6-311++G(d,p)) = -604.1750163 Eh
E(XMCQDPT2(10,8)/6-311++G(d,p)) = -605.8035181 Eh
0 1

C	-0.466780890791	1.750789695806	-0.418860165328
C	0.587042577498	2.339153030448	-1.302701303352
H	1.542452971709	1.903563095888	-1.032683384784
H	0.641832062495	3.411402370112	-1.159308363985
H	0.356560447849	2.101967045226	-2.330360256786

O	-1.313553619150	0.997842187278	-0.698999871613
O	-0.288930011917	2.240747737287	0.865665009682
O	-1.148889678198	1.709890797835	1.766454262933
O	1.143492596842	-1.702716939359	1.766028165919
O	0.285771071713	-2.236524755993	0.864870340662
C	0.466576565771	-1.750493429784	-0.420741375472
O	1.314109775178	-0.998561188344	-0.701270846784
C	-0.585332506398	-2.341418384592	-1.305144298542
H	-0.640053603385	-3.413322953213	-1.159128465946
H	-0.352886777570	-2.106824723617	-2.332959188975
H	-1.541410982653	-1.905493585029	-1.038048763183

AcO_4Ac

E(CASSCF(10,8)/6-311++G(d,p)) = -604.1888110 Eh
E(XMCQDPT2(10,8)/6-311++G(d,p)) = -605.8454819 Eh
0 1

C	0.113443984205	1.666193442663	-0.402438193154
C	1.082059557395	2.410551434616	-1.278222129610
H	1.846037951737	1.714726255226	-1.607207694426
H	1.560206242026	3.211765432651	-0.729093917810
H	0.547583247653	2.799955327798	-2.132263701432
O	-0.976093840720	1.328864525268	-0.682033447383
O	0.713136675658	1.415800545566	0.780731587551
O	-0.201132008554	0.637552035206	1.731686177455
O	0.201075327585	-0.637500362159	1.731737074411
O	-0.713151286665	-1.415803324540	0.780800199562
C	-0.113446085197	-1.666188873694	-0.402366706123
O	0.976072032690	-1.328811640247	-0.681978406387
C	-1.082038377393	-2.410580134597	-1.278152709598
H	-1.560244676011	-3.211774312616	-0.729045238795
H	-0.547517555642	-2.800002965816	-2.132158465434
H	-1.845991188714	-1.714747385221	-1.607181056437

$[AcO\cdots O_2\cdots OAc]^{\ddagger}$

E(CASSCF(10,8)/6-311++G(d,p)) = -604.1887278 Eh
E(XMCQDPT2(10,8)/6-311++G(d,p)) = -605.8470638 Eh
$\tilde{\nu}^{\ddagger}$ = -202.32 cm^{-1}
0 1

C	0.179912476018	1.673799265246	-0.376287101250
C	1.158271206899	2.382475413584	-1.272623449185
H	1.884836391533	1.656903865017	-1.621591613451
H	1.681833926828	3.161939248025	-0.733768298145
H	0.619999549925	2.795780176508	-2.113056697073
O	-0.923578632907	1.368796405158	-0.642020672485
O	0.785572159917	1.413436084355	0.797460036750
O	-0.175116371686	0.629336365583	1.782770302129
O	0.175795628735	-0.629565908598	1.782647062145
O	-0.785219115876	-1.413513699364	0.797564011772
C	-0.179800315048	-1.674076791249	-0.376263469253

O	0.923775321899	-1.369489915173	-0.642119825485
C	-1.158547494892	-2.382286949541	-1.272541650183
H	-1.683735676934	-3.160384540931	-0.733295332114
H	-0.620293495927	-2.797238766598	-2.112187359979
H	-1.883705556421	-1.655910249958	-1.622797285550

AcO…O_2…AcO

E(CASSCF(10,8)/6-311++G(d,p)) = -605.8190452 Eh
E(XMCQDPT2(10,8)/6-311++G(d,p)) = -605.8190452 Eh
0 1

C	0.134795624783	1.830329652555	-0.595945973176
C	1.348515921674	1.954829392582	-1.472985221679
H	1.794794068028	0.970167978257	-1.551337280388
H	2.071727391040	2.648927303881	-1.063474749009
H	1.028863073509	2.278798263043	-2.454163642787
O	-0.931320390436	1.429811917564	-0.911010223971
O	0.374677393114	2.210683586112	0.651186630145
O	-0.598087230334	0.103273136465	3.665269760463
O	0.599674719430	-0.102826236436	3.664782956448
O	-0.373941075093	-2.211080531174	0.650104988091
C	-0.135136294809	-1.827962599381	-0.596311429176
O	0.929262236331	-1.422213415036	-0.910466904937
C	-1.348383252651	-1.956663501718	-1.473309355709
H	-2.070161581967	-2.651250287015	-1.062037647907
H	-1.028684048507	-2.281946089307	-2.454049709761
H	-1.796432626115	-0.972933866450	-1.553413182515

AllylO_4Allyl (1,4-diallyltetraoxidane)

AllylO_2

E(CASSCF(5,4)/6-311++G(d,p)) = -266.2060603 Eh
0 2

C	2.228900211800	-0.125821129100	-0.194823662600
H	2.967822698400	-0.901746752200	-0.288098891000
H	2.547942769900	0.877479366500	-0.423735023900
C	0.996106610600	-0.394748409300	0.186037489900
H	0.700060896700	-1.407164702000	0.405507613700
C	-0.060912235900	0.651613518900	0.367134402800
H	0.286801781700	1.625813913100	0.052451740300
H	-0.403017908400	0.701575758900	1.392464661400
O	-1.202161792900	0.397516892900	-0.455406789800
O	-1.941046032000	-0.600687457800	0.052524459300

AllylO_2 + AllylO_2

E(CASSCF(10,8)/6-311++G(d,p)) = -532.4122385 Eh
E(XMCQDPT2(10,8)/6-311++G(d,p)) = -533.9507436 Eh
0 1

C	4.581432027400	-1.560496662500	4.171238539200
H	5.515493071600	-1.035880527300	4.076351514400
H	3.928971405400	-1.549697977600	3.314221425600
C	4.257745837400	-2.189445347100	5.283143640100

H	4.925559048100	-2.178661236700	6.129104237500
C	2.992135050700	-2.969593809600	5.454361900900
H	3.193541323000	-4.012460928100	5.664173490700
H	2.350218070700	-2.891444699200	4.587525149400
O	2.301307715500	-2.427768008600	6.583244781200
O	1.225897027200	-3.173555135400	6.876465054000
O	-1.973763703000	1.778249768000	-6.922521763000
O	-3.257675184000	2.102726938000	-6.712975212200
C	-3.458234614300	2.565612158400	-5.376180933700
H	-2.982559909900	1.873355840300	-4.695935522100
H	-4.533211970500	2.513760468400	-5.252436356300
C	-2.961813064600	3.966641287100	-5.192208612900
H	-3.359452917200	4.700776103000	-5.874509158100
C	-2.108358987000	4.322979477100	-4.253820241200
H	-1.797845663200	5.346242507700	-4.138431672300
H	-1.687633946900	3.607367284700	-3.567555351000

$AllylO_2 \ldots AllylO_2$

E(CASSCF(10,8)/6-311++G(d,p)) = -532.4160074 Eh

E(XMCQDPT2(10,8)/6-311++G(d,p)) = -533.9569517 Eh

0 1

C	3.415217909372	0.234302537974	-0.819804666371
H	4.349415264052	0.758227958900	-0.918925264560
H	2.748205511057	0.261564825952	-1.664896378583
C	3.107328050092	-0.413078786934	0.286273324741
H	3.787596152330	-0.418413871312	1.122539105310
C	1.840745230326	-1.190954878274	0.457872187147
H	2.037823995581	-2.228500424428	0.695012302333
H	1.205776984320	-1.126131325579	-0.413403642920
O	1.135218732221	-0.624402201235	1.571509727838
O	0.066214910816	-1.370656321263	1.880700153239
O	-0.816090563087	-0.008731710629	-1.926756862585
O	-2.100002044098	0.315745459874	-1.722934003807
C	-2.310085120321	0.781790392644	-0.384391653864
H	-1.844353963572	0.088597156419	0.300466614753
H	-3.386321891256	0.731640313017	-0.273123849764
C	-1.812548106287	2.181960097030	-0.199197755429
H	-2.216775717547	2.920005982481	-0.873661755291
C	-0.952303397993	2.530790194295	0.736440227319
H	-0.643660432598	3.554495013455	0.855429365987
H	-0.525524990079	1.810518227128	1.413261832372

$[AllylOO \cdots OOAllyl]^{\ddagger}$

E(CASSCF(10,8)/6-311++G(d,p)) = -532.4099656 Eh

E(XMCQDPT2(10,8)/6-311++G(d,p)) = -533.9676818 Eh

$\tilde{\nu}^{\ddagger}$ = -181.44 cm^{-1}

0 1

C	3.957919267663	0.275117438953	-0.217351898732
H	4.856811020788	0.652922540282	0.236857322175
H	3.739569109866	0.611310849288	-1.217128225151

C	3.167270504440	-0.564195050840	0.421209306487
H	3.405576754670	-0.878587859634	1.424523940174
C	1.928303384683	-1.157289335818	-0.175386121693
H	1.996556352616	-2.237507279074	-0.224691865258
H	1.732870586525	-0.763047819268	-1.164206411351
O	0.845444795254	-0.820381632400	0.683969057531
O	-0.273735457853	-1.538558665421	0.310818888532
O	-1.085165507589	-0.561440659658	-0.954754953154
O	-2.352226113397	-0.331642359044	-0.467982012919
C	-2.340734015531	0.784718903832	0.412343104878
H	-1.616269027076	0.609206660150	1.195441656596
H	-3.336644588691	0.764763575373	0.839877440852
C	-2.094938503744	2.080153347659	-0.300690461757
H	-2.750626481254	2.293408041109	-1.130203201844
C	-1.163534442289	2.943804828225	0.050416718654
H	-1.042446433507	3.879444763001	-0.466653251810
H	-0.488124691344	2.746568349945	0.865999974753

AllylO_4Allyl

E(CASSCF(10,8)/6-311++G(d,p)) = -532.4143240 Eh
E(XMCQDPT2(10,8)/6-311++G(d,p)) = -533.9735368 Eh
0 1

C	3.679544421532	0.484629091114	0.109488018925
H	4.528396705988	0.759163974973	0.710502374479
H	3.435931631857	1.134779031172	-0.714167798748
C	2.979368884816	-0.602081664631	0.367724412640
H	3.241115222745	-1.232102171252	1.202256717075
C	1.813886008404	-1.059308708722	-0.457652653150
H	2.010895347668	-2.036201097490	-0.886534435217
H	1.595586962567	-0.356167090820	-1.253157105746
O	0.709366724383	-1.158718512913	0.419653534371
O	-0.411773346825	-1.775583608618	-0.372857358029
O	-1.182909202662	-0.753615388584	-0.851440762695
O	-2.209372988006	-0.452547474802	0.201784613592
C	-1.885543025568	0.791150517290	0.787577414025
H	-0.892115970616	0.757282985866	1.213330357871
H	-2.608917898995	0.859331100231	1.594655385496
C	-2.051120169552	1.947162712022	-0.155028700230
H	-2.984541212158	1.983015389611	-0.694501863303
C	-1.150271393333	2.895182698691	-0.320347315197
H	-1.324705143936	3.727617656990	-0.979102721920
H	-0.206945044977	2.875779197325	0.200226892516

[AllylO···O_2···OAllyl]‡

E(CASSCF(10,8)/6-311++G(d,p)) = -532.4131972 Eh
E(XMCQDPT2(10,8)/6-311++G(d,p)) = -533.9730312 Eh
$\tilde{\nu}^‡$ = -257.06 cm^{-1}
0 1

C	4.592523937000	-0.876577640700	0.302996071300
H	5.331264196200	-0.630573000700	1.045272021600

H	4.944597392300	-1.362947176600	-0.591222455700
C	3.317336567300	-0.597930310900	0.486397258400
H	2.994642832400	-0.101818889500	1.387411980500
C	2.233625721000	-0.947371721000	-0.491998055700
H	1.503972247300	-1.603999947000	-0.029875808900
H	2.647476325500	-1.432836280500	-1.369199452000
O	1.607089368800	0.262323373100	-0.848914494000
O	0.329991371000	-0.204473263500	-1.755661656700
O	-0.667315199200	0.540090854400	-1.424679950200
O	-1.373850034500	-0.170515477300	-0.102379990400
C	-1.769748484500	0.903605689000	0.709200578600
H	-0.925388360700	1.552367755400	0.912176446700
H	-2.055976942200	0.414260866400	1.637350692000
C	-2.933779926900	1.670808505300	0.150337970900
H	-3.782001876100	1.077350568200	-0.152730410800
C	-2.965397452600	2.983590323600	0.037432290700
H	-3.832440714900	3.494297283300	-0.342820325300
H	-2.125418547400	3.596684821000	0.319611980100

AllylO…O_2…AllylO

E(CASSCF(10,8)/6-311++G(d,p)) = -532.4464683 Eh
E(XMCQDPT2(10,8)/6-311++G(d,p)) = -533.9448169 Eh
0 1

C	4.668339019300	-0.850064747000	0.265061123400
H	5.443308894200	-1.131835304500	0.955829039900
H	4.932903801400	-0.830329854800	-0.779465869200
C	3.453249881300	-0.547358422600	0.677462807000
H	3.215249269700	-0.562106008800	1.729353154600
C	2.325917747300	-0.169606389600	-0.241311007000
H	1.515431713500	-0.894199741600	-0.175197775800
H	2.652806908400	-0.132807739500	-1.276708442900
O	1.732912574800	1.032403543100	0.117907848700
O	0.101943776300	-0.385970958900	-3.459322086500
O	-0.841159517800	0.333449949100	-3.194460890500
O	-1.331675291000	-0.429059593000	0.319611080000
C	-1.672516169100	0.789902172100	0.891246283500
H	-0.898422827200	1.522904989600	0.689515432500
H	-1.664781415600	0.592722124400	1.963790570600
C	-3.026429791900	1.274724935800	0.456555145900
H	-3.836290787000	0.571325575100	0.569646297700
C	-3.251521128600	2.478596104500	-0.031424555600
H	-4.239807969000	2.795555989100	-0.314530912200
H	-2.455485033000	3.192465865600	-0.164956946100

AceO$_4$Ace (1,1’-tetraoxidanediylbis(propan-2-one))

AceO$_2$

E(CASSCF(5,4)/6-311++G(d,p)) = -341.1144991 Eh
0 2

C	-0.890044792300	1.394513323000	0.212743396200

H	-0.211206913600	1.872718059300	-0.485763506600
H	-0.509572667400	1.580884951900	1.212178925400
H	-1.880856755000	1.812121477800	0.103650239100
C	-0.949705659400	-0.085164212100	-0.061114903000
C	0.283002802300	-0.942245584800	0.206742788000
H	0.118310431800	-1.499803988700	1.120529711300
H	0.431236899900	-1.628783312500	-0.613309687100
O	1.469790606700	-0.201991200300	0.435373636900
O	1.962228660300	0.271328524100	-0.723543748100
O	-1.925549613400	-0.637179037800	-0.447023852300

$AceO_2$ + $AceO_2$

E(CASSCF(10,8)/6-311++G(d,p)) = -682.2282123 Eh
E(XMCQDPT2(10,8)/6-311++G(d,p)) = -684.1594969 Eh
0 1

C	4.947959259700	0.389979552100	-2.954053938600
O	3.884194699700	0.538983381000	-2.451741471700
C	5.030032962700	0.505051252400	-4.472581287200
H	4.226060106400	-0.058651422000	-4.921663881800
H	4.933958839500	1.549915209900	-4.740808473500
C	6.206792323700	0.139370787200	-2.166275912000
H	6.979255540900	0.847497156500	-2.449451492600
H	6.585376143200	-0.854733787600	-2.380357159600
H	5.984598560900	0.227019458300	-1.112254531200
O	6.270412324300	0.095384361000	-5.022426548500
O	6.353751812200	-1.246898235500	-5.053216942800
O	-3.956108768500	-0.661056162100	5.826285063900
O	-4.562861510700	0.131415550500	4.928306638400
C	-5.316678948300	-0.644791919700	4.008397459200
H	-4.661858767000	-1.346091414100	3.508315451300
H	-6.087748355500	-1.186356498900	4.539153652900
C	-5.965678261500	0.257627353700	2.967226318300
O	-6.678159312700	-0.254440605100	2.169471606300
C	-5.673148330600	1.734364921400	2.986180192200
H	-5.976187196500	2.162816046600	3.936131566500
H	-6.205657702100	2.209409943200	2.174472533100
H	-4.605773506200	1.904582937000	2.885950075200

$AceO_2$…$AceO_2$

E(CASSCF(10,8)/6-311++G(d,p)) = -682.2331323 Eh
E(XMCQDPT2(10,8)/6-311++G(d,p)) = -684.1681032 Eh
0 1

C	1.656680590001	0.323391190413	-0.517069462454
O	0.468390780912	0.303062949952	-0.539046534025
C	2.375819263075	0.541432773208	-1.843046428503
H	1.938562519403	-0.092644274703	-2.599896199340
H	2.255529254379	1.579581572429	-2.127708104138
C	2.461538216288	0.187663938604	0.746548927054
H	3.174731408976	1.001831048545	0.828729139021
H	3.029803774464	-0.736534249363	0.721781134272
H	1.793660967932	0.183190385269	1.595915604605

O	3.775114597469	0.332092021062	-1.787328344487
O	4.054905012690	-0.984116694293	-1.771895212341
O	-0.802991729156	-0.856014372018	2.859469613139
O	-1.658074512092	-0.042510047057	2.224479187145
C	-2.262685648894	-0.711107706526	1.124019578414
H	-1.487417613764	-1.146685192084	0.510335553953
H	-2.929381681184	-1.478769286124	1.490311185971
C	-3.045201767600	0.281349223387	0.277069524081
O	-3.836109967854	-0.153662020131	-0.493330430726
C	-2.755940047645	1.751121393842	0.422461335625
H	-2.994279734892	2.079127412623	1.429176006534
H	-3.347220600462	2.299755287574	-0.297354954526
H	-1.698470069046	1.930843122840	0.261954062966

[AceOO···OOAce]‡

E(CASSCF(10,8)/6-311++G(d,p)) = -682.2265497 Eh
E(XMCQDPT2(10,8)/6-311++G(d,p)) = -684.1826817 Eh
$\tilde{\nu}^{\ddagger}$ = -194.44 cm^{-1}
0 1

C	1.709758844842	1.347810283604	-0.296748890468
O	1.031470862697	2.234524419847	-0.701981226864
C	2.023944639602	0.193516674010	-1.241478562913
H	1.204536841244	0.044000337193	-1.927142127594
H	2.914422390117	0.460807046399	-1.801261236482
C	2.343902279785	1.346708150537	1.068234460393
H	3.419475665707	1.222289755531	0.975910641673
H	1.971417238804	0.512290195113	1.649321724492
H	2.120573695615	2.280362838181	1.565353834407
O	2.347255727025	-1.020743629954	-0.589798070745
O	1.249075489497	-1.847320187794	-0.520034782682
O	0.366483779546	-1.491321552031	1.019378288833
O	-0.547932478707	-0.497813874067	0.728062717725
C	-1.749393232712	-1.081920086381	0.257146962648
H	-1.570642185766	-1.586315007899	-0.682751803435
H	-2.116706247295	-1.795358166054	0.984398259294
C	-2.807805043360	-0.004407793326	0.050183660633
O	-3.822886597878	-0.333218806116	-0.468330165933
C	-2.535632458653	1.398224278265	0.522637616866
H	-2.297220129393	1.393669654922	1.581986984604
H	-3.410305658042	2.006414827343	0.339773939598
H	-1.676830412478	1.810199120123	0.002712960202

AceO$_4$Ace

E(CASSCF(10,8)/6-311++G(d,p)) = -682.2319766 Eh
E(XMCQDPT2(10,8)/6-311++G(d,p)) = -684.1908033 Eh
0 1

C	1.648835710432	1.340496091079	-0.282125324424
O	1.018295744743	2.151530582832	-0.879103709660
C	2.227647921361	0.153795701153	-1.043827578613

H	1.635251646470	-0.043254679141	-1.925097105446
H	3.232549742142	0.423927334726	-1.356844650281
C	1.987657762978	1.471958546642	1.180006615497
H	3.065731591042	1.435554772965	1.312676183104
H	1.564598169348	0.645881264785	1.737355648831
H	1.603028691095	2.411087957639	1.553020915486
O	2.391355349194	-1.012371811343	-0.273208909768
O	1.157639543839	-1.860645412748	-0.436837892631
O	0.428126676031	-1.774930495570	0.710752868467
O	-0.495881773899	-0.594483063042	0.563220290790
C	-1.703447268363	-1.089530683942	0.030117557172
H	-1.549250870229	-1.487758356763	-0.965206298915
H	-2.102924102340	-1.867770613270	0.669911903499
C	-2.739445262428	0.029280964137	-0.054728818953
O	-3.772592142240	-0.228074733535	-0.577352327795
C	-2.421590851402	1.378172396813	0.530685127453
H	-2.142395478164	1.274827273351	1.574696979078
H	-3.289272073236	2.015992391026	0.436786774637
H	-1.576955712209	1.818713051718	0.010676933758

[AceO···O_2···OAce]‡

E(CASSCF(10,8)/6-311++G(d,p)) = -682.2305255 Eh
E(XMCQDPT2(10,8)/6-311++G(d,p)) = -684.1896603 Eh
$\tilde{\nu}^‡$ = -289.88 cm^{-1}
0 1

C	1.743864957310	1.282950346904	-0.347572198176
O	1.154039900589	2.020982114382	-1.068243590394
C	2.507964301673	0.105031004600	-0.942891143303
H	2.160793700504	-0.085183489179	-1.949344143206
H	3.561106568927	0.373864808057	-0.991435572788
C	1.841361499355	1.489825705904	1.142356292736
H	2.885825845048	1.546535820041	1.437940578170
H	1.399920763374	0.650118517089	1.664178861542
H	1.334917977710	2.406172190297	1.412509145311
O	2.466084276644	-1.055042604404	-0.162721117801
O	1.065910369214	-1.833867298828	-0.633854841887
O	0.332745089077	-2.041091590850	0.405769734414
O	-0.630474837675	-0.741124774684	0.614798472541
C	-1.856124996427	-1.102492125838	0.031560088267
H	-1.727374085094	-1.324061069923	-1.023651462165
H	-2.285407439545	-1.962692133154	0.529132139338
C	-2.849256846358	0.055128657980	0.136682997885
O	-3.983775930531	-0.171802395460	-0.123520311945
C	-2.347893431066	1.418117734738	0.530091102390
H	-1.904940946971	1.375044859575	1.520406641103
H	-3.174411727920	2.114787307183	0.523361664907
H	-1.573995889021	1.746067356454	-0.156823723382

AceO…O_2…AceO

E(CASSCF(10,8)/6-311++G(d,p)) = -682.2684973 Eh
E(XMCQDPT2(10,8)/6-311++G(d,p)) = -684.1610708 Eh
0 1

C	1.838661968400	0.841870548900	-0.217401434600
O	0.800560606300	0.992477914800	-0.778713623100
C	2.938426208200	0.062722561600	-0.931488207300
H	2.554308686500	-0.918278891100	-1.199692224700
H	3.172866554600	0.570650794600	-1.864409190600
C	2.118107693900	1.383936496700	1.157925027200
H	2.986746015400	2.034308421700	1.132688270400
H	2.352294809500	0.568917565000	1.835413343000
H	1.253204942400	1.924903465700	1.514420046700
O	4.108401476400	-0.102539046400	-0.224305185200
O	0.784643362600	-3.379635968100	-0.603514850200
O	0.638017095200	-4.060044271400	0.392642067600
O	-1.304601187200	-0.735251047000	0.904908740600
C	-2.101710866500	-0.586818724700	-0.211072853000
H	-1.429923199600	-0.402783328600	-1.046775196300
H	-2.635633673300	-1.510352958100	-0.412863588700
C	-3.094687584500	0.568096721400	-0.149638341300
O	-4.045245763100	0.543982275600	-0.861695211900
C	-2.797418813600	1.708445213900	0.788559840200
H	-2.809556180900	1.356445856400	1.815066328100
H	-3.537693995300	2.484787803000	0.653380698000
H	-1.802280884300	2.094851256300	0.591905657100

$AceO_4$-*S*-BuOH (*S*-1-((1-hydroxybutan-2-yl)tetraoxidaneyl)propan-2-one)

$AceO_2$

-follow-this-link-

S-BuOH-O_2

E(CASSCF(5,4)/6-311++G(d,p)) = -381.3250273 Eh
0 2

C	-0.251361330500	1.146235246600	0.705052431700
H	0.583209334700	1.599693524100	1.224728225900
H	-1.141948320900	1.319940693300	1.304849876200
C	-0.000812159500	-0.357066371200	0.633851939600
H	0.292574951400	-0.689373502600	1.624001970200
C	1.025418325100	-0.815954768400	-0.394095025500
H	0.685815797600	-0.538584134800	-1.385112398900
H	1.061223585200	-1.901059008400	-0.358723249000
C	2.419683918400	-0.242257272600	-0.144038979500
H	3.128692641600	-0.668989713200	-0.845526039400
H	2.771633702700	-0.472958604400	0.858826035200
H	2.436259626200	0.834301006900	-0.272850910100
O	-0.338275332600	1.786965094000	-0.530251525100
H	-1.065338275700	1.437446235200	-1.015571260400
O	-1.232364497100	-1.085065240100	0.445807800400
O	-1.910034967600	-0.665724184400	-0.631159892200

$AceO_2$ + *S*-BuOH-O_2

E(CASSCF(10,8)/6-311++G(d,p)) = -722.4409972 Eh
E(XMCQDPT2(10,8)/6-311++G(d,p)) = -724.5522863 Eh
0 1

C	8.585921509500	-0.243102579500	2.654019242100
H	9.278839647400	-0.413966820800	1.836083911700
H	8.836113205300	-0.946275419700	3.441654783000
H	8.681654658600	0.768212278300	3.022922707000
C	7.167713859700	-0.465867447900	2.198838610700
O	6.302080295900	0.329784078800	2.352225133100
C	6.81693286700	-1.754095619100	1.462183829200
H	5.884422218200	-2.145444968900	1.842442677100
H	6.710072844300	-1.526240427100	0.408678325500
O	7.819123061600	-2.753541557400	1.523982765400
O	7.814581305400	-3.353708193600	2.727885780600
O	-5.568104814200	-0.546606258400	-3.283214566500
O	-5.536059485700	0.337219566800	-2.275739537000
C	-6.738729782800	1.126854831100	-2.218471194500
H	-6.990619806900	1.387335450000	-3.238368216900
C	-6.330136020300	2.375589674200	-1.455422117400
H	-7.192659586400	3.014885371500	-1.329030369000
H	-5.973587278200	2.093660595600	-0.466248878700
C	-7.849805872300	0.315647642000	-1.564150970100
H	-7.900609322200	-0.639816606000	-2.075068546800
H	-7.577766483400	0.106233538500	-0.532166198200
C	-9.220540364800	0.991677737100	-1.625224070100
H	-9.496866832100	1.227058952700	-2.649386208600
H	-9.253655918600	1.911068465000	-1.049700768700
H	-9.980051405100	0.329581689300	-1.223218882700
O	-5.373921559300	3.127503577200	-2.139772904000
H	-4.577865628500	2.631902889500	-2.224403978100

$AceO_2$…*S*-BuOH-O_2

E(CASSCF(10,8)/6-311++G(d,p)) = -722.4494579 Eh
E(XMCQDPT2(10,8)/6-311++G(d,p)) = -724.5647167 Eh
0 1

C	4.126064412843	0.668561023416	0.663442246060
H	4.827847191677	0.481869740906	-0.143098145358
H	4.357301008593	-0.030971323226	1.460077242773
H	4.225732688105	1.682314567834	1.024145890196
C	2.714504407624	0.452282620743	0.189633304757
O	1.859522245679	1.268219861866	0.328525149811
C	2.355662470646	-0.838613516763	-0.532747705585
H	1.433632119828	-1.237140382590	-0.140528654157
H	2.226854511306	-0.616442912645	-1.584798904815
O	3.373537597371	-1.823739656082	-0.481914930897
O	3.384978435159	-2.429819309017	0.718952625095
O	-1.155560323718	-1.496052731341	-1.315909534308
O	-1.099207749618	-0.623860023212	-0.300644956800
C	-2.285447240554	0.193602077027	-0.228380093521

H	-2.543634362257	0.453031933822	-1.246497530296
C	-1.854977353376	1.443922540565	0.522031828827
H	-2.729027906675	2.058956254142	0.693474073236
H	-1.454736928377	1.160816021083	1.494022865200
C	-3.400398399311	-0.601232334564	0.440878413926
H	-3.455687090281	-1.566616650452	-0.050866984674
H	-3.131193164809	-0.791299111320	1.477609768017
C	-4.769194943431	0.077374309600	0.365073440419
H	-5.044819516436	0.289565175961	-0.664311472133
H	-4.799173325610	1.010138784161	0.918418994547
H	-5.530578889620	-0.573120215495	0.782618386707
O	-0.945968835537	2.210165634088	-0.199566893443
H	-0.068233191944	1.881288006243	-0.082459757998

[AceOO···OO-*S*-BuOH]‡

E(CASSCF(10,8)/6-311++G(d,p)) = -722.4416474 Eh
E(XMCQDPT2(10,8)/6-311++G(d,p)) = -724.5804873 Eh
$\tilde{\nu}^{\ddagger}$ = -196.69 cm^{-1}
0 1

C	2.926439594959	-0.028866138070	1.407852436956
H	3.867363670114	-0.515455931329	1.166952429520
H	2.229004121475	-0.802299842127	1.706610999632
H	3.067027886174	0.679751297259	2.211972273210
C	2.390483033150	0.688068510863	0.200041905485
O	2.095328999776	1.841941065444	0.205714356922
C	2.291275404989	-0.081305935902	-1.110459319437
H	1.522053593264	0.344709172990	-1.735136499692
H	3.251003619482	0.001873251160	-1.611393831700
O	2.073403476208	-1.467851895335	-0.938250118967
O	0.736977489391	-1.773796449463	-1.103158119907
O	-0.081242004887	-1.598811817830	0.493119452731
O	-0.370381884850	-0.259879712809	0.650970085152
C	-1.638685465679	0.068089938953	0.062265820501
H	-1.643824006059	-0.349004338293	-0.937059655898
C	-1.639281652749	1.587376263982	-0.026732214938
H	-2.618985298721	1.910330485381	-0.353948044660
H	-1.467606051280	2.004650374179	0.963306841787
C	-2.773407862873	-0.504943387595	0.904900986564
H	-2.530605705326	-1.537645193357	1.128815723757
H	-2.812300588720	0.022005888606	1.855994016433
C	-4.137251885698	-0.453517805851	0.213989853526
H	-4.112711986908	-0.975095005645	-0.738914568535
H	-4.470412503801	0.562673155752	0.029820793142
H	-4.887654744033	-0.934099780639	0.833118259322
O	-0.715620806812	2.075609435339	-0.949954345788
H	0.132923244649	2.184683979239	-0.551072807906

$AceO_4$-*S*-BuOH

E(CASSCF(10,8)/6-311++G(d,p)) = -722.4468471 Eh

E(XMCQDPT2(10,8)/6-311++G(d,p)) = -724.5861936 Eh
0 1

C	2.770970522736	0.061042749424	1.557830496851
H	3.721348137562	-0.454045726886	1.449771163998
H	2.031966894199	-0.677268090049	1.844200708602
H	2.846151515144	0.826763727886	2.317256705822
C	2.371434437789	0.691466704060	0.251542198220
O	2.098253656966	1.846375640748	0.146681585624
C	2.400939140924	-0.176869127810	-0.999849016428
H	1.762775098682	0.247213977912	-1.760553211528
H	3.424138916016	-0.182105555186	-1.366753991007
O	2.085573316089	-1.528477593713	-0.772034973916
O	0.617088415700	-1.720083606589	-1.033997204891
O	-0.039102770850	-1.711297195966	0.166205950015
O	-0.324540276530	-0.277810002124	0.494605906808
C	-1.589477711136	0.079357919723	-0.061263777447
H	-1.617361225158	-0.272364331713	-1.086507246717
C	-1.583427138688	1.604017684161	-0.074624952428
H	-2.575773297567	1.945798661935	-0.338977255576
H	-1.364143631818	1.970975242766	0.925986756057
C	-2.724776379390	-0.526442993118	0.759080513984
H	-2.480179371627	-1.565778808400	0.950462646836
H	-2.764753440275	-0.033384375397	1.728145642192
C	-4.088959112168	-0.456052562016	0.070799892105
H	-4.065262590465	-0.951783688942	-0.895959633901
H	-4.420907437211	0.565047282915	-0.086884937283
H	-4.840438197592	-0.951984871972	0.676508857012
O	-0.703204695935	2.133299090537	-1.015603863576
H	0.169233102277	2.197764760183	-0.660972671884

[AceO···O_2···O-*S*-BuOH]‡

E(CASSCF(10,8)/6-311++G(d,p)) = -722.4446964 Eh
E(XMCQDPT2(10,8)/6-311++G(d,p)) = -724.5906935 Eh
$\tilde{\nu}^{\ddagger}$ = -318.16 cm^{-1}
0 1

C	2.728887997660	0.036760383685	1.535572056233
H	3.684650913880	-0.477771740589	1.492647495112
H	1.977034705179	-0.694865104325	1.805782181821
H	2.761861734052	0.824508719739	2.275294780823
C	2.393354968358	0.626199102331	0.192124441900
O	2.103330352349	1.772174098383	0.043544875129
C	2.504680898398	-0.278109426169	-1.029308490573
H	1.916741158819	0.126857937208	-1.841137102378
H	3.548914675055	-0.280985004339	-1.337609251911
O	2.186008578316	-1.616993085118	-0.787945089076
O	0.509258891880	-1.745573092415	-1.089624649946
O	-0.133423197661	-1.792679913847	0.025200687818
O	-0.412513775866	-0.262720193034	0.500994608278
C	-1.666748001740	0.130609535452	-0.036685458643
H	-1.711252343961	-0.189417662700	-1.072687616697

C	-1.629564360024	1.657438007060	-0.019045552380
H	-2.618892493679	2.025374064723	-0.258240533680
H	-1.384464739286	1.999191533817	0.984782939351
C	-2.813311297792	-0.470282368063	0.772539126955
H	-2.590264981634	-1.519612501048	0.936806669855
H	-2.836836680503	-0.003068931229	1.754888485126
C	-4.179591982719	-0.352310259542	0.095325366899
H	-4.172350159204	-0.821436881475	-0.884985198123
H	-4.490000205205	0.679659851250	-0.032570437340
H	-4.938589716712	-0.848224105446	0.691702504120
O	-0.758400262928	2.187959883490	-0.966147226971
H	0.131814609528	2.174411360501	-0.652081386215

AceO…O_2…*S*-BuOH-O

E(CASSCF(10,8)/6-311++G(d,p)) = -722.4810995 Eh
E(XMCQDPT2(10,8)/6-311++G(d,p)) = -724.5133795 Eh
0 1

C	3.549093962500	-0.175248716500	1.358640567000
H	4.558066066200	-0.050485644700	0.978306148100
H	3.372802371400	-1.242799394100	1.441825468600
H	3.452421839000	0.299419954800	2.324863715100
C	2.563071014900	0.436219573600	0.401369048400
O	1.837024894100	1.329371989300	0.709095389100
C	2.479654814700	-0.117573670300	-1.015104325900
H	1.513839034000	-0.605144114500	-1.126087999200
H	2.496395179200	0.707382454700	-1.722186994700
O	3.451032185000	-1.035693157600	-1.345744546100
O	0.091913824700	-3.849969981600	0.392901634300
O	0.659729248700	-3.035997136700	1.094142483400
O	-0.962840043300	-0.387589333700	-0.539496366900
C	-2.085512813500	0.439398561800	-0.469471153300
H	-2.272865136900	0.698138290900	-1.511886964000
C	-1.750181718300	1.723462047200	0.285549493100
H	-2.625727392800	2.360261455200	0.314008424900
H	-1.488304827200	1.477717769400	1.314108459100
C	-3.273861213900	-0.342334489700	0.094344896500
H	-3.318841950400	-1.286096776200	-0.440252634600
H	-3.075978146200	-0.586427076700	1.136603174600
C	-4.620129440000	0.374087180800	-0.023748253700
H	-4.822720212400	0.661823480400	-1.052289857800
H	-4.666088173800	1.268968819000	0.587866537400
H	-5.422888764700	-0.281295557300	0.298742227500
O	-0.734818353300	2.445892469400	-0.333504982900
H	0.108652622300	2.112066651900	-0.069630751000

R-BuOH-O_4-*R*-BuOH ((2*R*,2’*R*)-2,2’-tetraoxidanediylbis(butan-1-ol))

R-BuOH-O_2

E(CASSCF(5,4)/6-311++G(d,p)) = -381.3250273 Eh
0 2

C	0.251342344700	1.146222910300	0.705068520500
H	1.141929775800	1.319930412100	1.304853242800
H	-0.583216911100	1.599660679000	1.224769136800
C	0.000825439500	-0.357083978800	0.633848322300
H	-0.292545953400	-0.689403703000	1.624000242700
C	-1.025412591200	-0.815978036600	-0.394090402500
H	-1.061216598500	-1.901082927000	-0.358703549700
H	-0.685815400900	-0.538595818300	-1.385103671600
C	-2.419672381800	-0.242270328700	-0.144052816600
H	-3.128680234900	-0.669002585800	-0.845537454800
H	-2.436241066600	0.834281755000	-0.272898613800
H	-2.771625531100	-0.472954896700	0.858811995600
O	0.338218222500	1.786999560300	-0.530213477800
H	1.065310707700	1.437550901900	-1.015534826600
O	1.232388821000	-1.085052813900	0.445771682100
O	1.910033358400	-0.665673130100	-0.631200329500

R-BuOH-O_2 + *R*-BuOH-O_2

E(CASSCF(10,8)/6-311++G(d,p)) = -762.6526130 Eh
E(XMCQDPT2(10,8)/6-311++G(d,p)) = -764.9507522 Eh
0 1

H	6.921233439700	1.469250187600	1.942688589700
C	6.849538333300	0.612621143100	1.284291052000
H	7.003318596800	-0.282836482300	1.879457776200
C	7.937423782800	0.726045806400	0.228801554300
H	7.723134169900	1.578512204700	-0.404832299700
C	9.342151819000	0.826999181500	0.805308577500
H	9.544107551700	-0.048357780700	1.414038616600
H	9.350934685000	1.684485212800	1.473890825900
C	10.430762645600	0.994389233800	-0.253896152800
H	11.403095328000	1.094970909900	0.217331869800
H	10.259313497300	1.882395451600	-0.856341075100
H	10.469625208300	0.140114909300	-0.920399001700
O	5.566892364100	0.640451219700	0.732326042300
H	5.424505615800	-0.125470385300	0.203810360900
O	7.822404124100	-0.365251440400	-0.708441555600
O	8.076704962600	-1.551543983900	-0.132552825100
O	-6.798683995300	-2.963175227900	0.407668076600
O	-6.753448252300	-1.647716812300	0.153192867600
C	-8.024716741700	-1.013686101400	0.387485155800
H	-8.420059270900	-1.434461058300	1.303007660800
C	-7.678455924400	0.450021195700	0.602431499000
H	-8.588881125700	1.014709324400	0.746933293200
H	-7.180747647900	0.837158243300	-0.284990734100
C	-8.952535460300	-1.282061869200	-0.790573124800
H	-8.950609426100	-2.351697775900	-0.970559003700
H	-8.541968361000	-0.812635071900	-1.681602491000
C	-10.390166744800	-0.814186653800	-0.558291580900
H	-11.016852999800	-1.109281210000	-1.393178341600
H	-10.463238148300	0.264312247300	-0.462774013100
H	-10.807453974800	-1.259451051800	0.340750626500

O -6.900290525700 0.650183167900 1.743661163600
H -6.067719626500 0.222324119700 1.642241179000

R-BuOH-O_2…*R*-BuOH-O_2

E(CASSCF(10,8)/6-311++G(d,p)) = -762.6612510 Eh
E(XMCQDPT2(10,8)/6-311++G(d,p)) = -764.9636557 Eh
0 1
H 2.028844991957 0.986778766483 2.116645243318
C 1.952292800436 0.134878034774 1.452892371219
H 2.093894069677 -0.766015096500 2.040985330842
C 3.038765990099 0.247870953954 0.396633976741
H 2.824504563603 1.101125385784 -0.236053881090
C 4.442017485727 0.352246611532 0.977487550822
H 4.643491761363 -0.522102737838 1.587828228026
H 4.448483030242 1.210885055733 1.644653366141
C 5.532648998738 0.518998861596 -0.079704208764
H 6.503748918086 0.620808624949 0.393722066531
H 5.362013628399 1.406192261873 -0.683595965504
H 5.573366205711 -0.336162383350 -0.744845767962
O 0.667904365357 0.174010861621 0.896846491948
H 0.479770292772 -0.614464075489 0.414218953981
O 2.927908047061 -0.842182117999 -0.539222494033
O 3.180575255368 -2.027494153838 0.039856020902
O -1.936564682297 -2.525288080940 0.220569096959
O -1.863205441942 -1.210378630671 -0.030554164227
C -3.121525303113 -0.546317284590 0.209743520169
H -3.521487897092 -0.964912463881 1.123806658391
C -2.759010132850 0.914010693211 0.432050100297
H -3.673758458082 1.485428196609 0.520313610673
H -2.219498125786 1.285464591100 -0.437934527705
C -4.053609469625 -0.797872435799 -0.969541466209
H -4.047426195187 -1.863677501969 -1.170965675804
H -3.651257102459 -0.308347749315 -1.853743603291
C -5.493300740922 -0.344428472929 -0.721566422244
H -6.121263176361 -0.624059877214 -1.561017974049
H -5.572940154683 0.730803478942 -0.599877810461
H -5.903999412629 -0.813544906929 0.168316398209
O -2.035506540444 1.116393692869 1.602522155065
H -1.136559776328 0.848482608479 1.480417997217

[*R*-BuOH-OO···OO-*R*-BuOH]‡

E(CASSCF(10,8)/6-311++G(d,p)) = -762.6468544 Eh
E(XMCQDPT2(10,8)/6-311++G(d,p)) = -764.9743183 Eh
$\tilde{\nu}^‡$ = -238.99 cm^{-1}
0 1
H 2.056390777968 2.295654744296 0.763835335347
C 1.541790016653 1.484677530529 0.263723452084
H 0.804900682317 1.086455572717 0.952097466974
C 2.564485932770 0.418203024288 -0.080284665828

H	3.263647472371	0.822450589553	-0.804297454280
C	3.313988813060	-0.088388043404	1.147649549145
H	2.603124693543	-0.522307965845	1.845891760701
H	3.750550582643	0.774548134093	1.644885926154
C	4.411300926527	-1.100413591689	0.822818652597
H	4.922098245521	-1.408414945016	1.729716618311
H	5.152400337216	-0.672859829724	0.152637425078
H	4.003476799983	-1.985661878854	0.348657944264
O	0.924109633912	2.041479432847	-0.863181718545
H	0.520553659708	1.356280223265	-1.370075780218
O	1.999966509876	-0.665915187231	-0.830776261156
O	1.004847270809	-1.325058306989	-0.097277833036
O	-0.461324023413	-1.256136411970	-1.107807392263
O	-1.135917157288	-0.067733970890	-0.933203191605
C	-2.118377149435	-0.164632082948	0.111013399015
H	-1.601140961963	-0.474469258358	1.011812361293
C	-2.632015587636	1.259668023241	0.273695346799
H	-3.458510565500	1.249642326492	0.971008732319
H	-3.012056308164	1.608355001502	-0.684974612628
C	-3.209240885479	-1.161571476112	-0.267924372401
H	-2.729169633677	-2.061746726323	-0.631932366765
H	-3.788290906389	-0.756861798824	-1.095227889339
C	-4.131865061279	-1.527338268640	0.896373690915
H	-4.843758198872	-2.283876702434	0.583256895265
H	-4.700332598473	-0.677246166076	1.259303258241
H	-3.566544283316	-1.932588851967	1.731197691397
O	-1.682671662885	2.138412278882	0.791265478295
H	-1.030602368635	2.349850722188	0.141971909272

R-BuOH-O_4-*R*-BuOH

E(CASSCF(10,8)/6-311++G(d,p)) = -762.6574317 Eh
E(XMCQDPT2(10,8)/6-311++G(d,p)) = -764.9850935 Eh
0 1

H	2.264905256036	2.427417789840	0.734338114196
C	1.655420597910	1.616099465085	0.355508028762
H	1.001961495552	1.287197753220	1.157779733847
C	2.566998058934	0.476785711550	-0.079967557791
H	3.245893945086	0.847014121346	-0.843430403102
C	3.364197270675	-0.123937480998	1.073167151723
H	2.680036347126	-0.519108703605	1.818877666748
H	3.907738843055	0.687233371795	1.552525308448
C	4.342605618538	-1.211925776775	0.635368184042
H	4.894290628535	-1.592482024372	1.489222148877
H	5.063203675732	-0.826081182846	-0.080968439882
H	3.822728018908	-2.042447574954	0.171827080467
O	0.904724295550	2.128748139208	-0.710394397449
H	0.546113177539	1.408004738984	-1.203850541216
O	1.847875934873	-0.498087068066	-0.820375161409
O	0.806841993450	-1.125399889536	0.121149016780
O	-0.244771923705	-1.431966309748	-0.678847464158

O	-1.064704906140	-0.181215520118	-0.836339608574
C	-2.132923678491	-0.214790654546	0.107321213777
H	-1.718059069422	-0.460976495410	1.079002422162
C	-2.649106882879	1.219778445357	0.144800320498
H	-3.568395074447	1.239543572802	0.714689730767
H	-2.883450156865	1.539210912471	-0.869345223979
C	-3.190480893071	-1.232723204305	-0.311449370574
H	-2.680949996200	-2.145370128422	-0.600488902496
H	-3.700705216029	-0.868032199861	-1.200438279947
C	-4.203661923480	-1.556148167709	0.787839812067
H	-4.894496901509	-2.320790561122	0.447809232457
H	-4.792711985144	-0.691349706093	1.075914549934
H	-3.707711204559	-1.932838499007	1.678442235578
O	-1.772064521332	2.105124311484	0.767271191571
H	-1.036379998072	2.301812125720	0.208403049109

[*R*-BuOH-O···O_2···O-*R*-BuOH]‡

E(CASSCF(10,8)/6-311++G(d,p)) = -762.6556684 Eh
E(XMCQDPT2(10,8)/6-311++G(d,p)) = -764.9902806 Eh
$\tilde{\nu}^‡$ = -310.07 cm^{-1}
0 1

H	2.304949132956	2.383795935668	0.682622720435
C	1.704195949447	1.558661119920	0.319957684159
H	1.077491789025	1.218859514300	1.139568741561
C	2.627315362284	0.433491815401	-0.134999804756
H	3.299532641020	0.827179023915	-0.895780472900
C	3.440539381209	-0.166172220046	1.008090803012
H	2.764942991248	-0.600764891504	1.740002425046
H	3.957015263638	0.648477711957	1.511241635486
C	4.453425073562	-1.213226859903	0.549876770855
H	5.007935204731	-1.603600684128	1.397549538220
H	5.169740485469	-0.786463908991	-0.147427243676
H	3.961228054936	-2.043626031044	0.056697430129
O	0.916221704345	2.052309049640	-0.726979519678
H	0.556320268311	1.322565994808	-1.206140133371
O	1.922372507261	-0.537542849937	-0.877023440550
O	0.730203316904	-1.205408768291	0.167280165119
O	-0.255628527532	-1.527152478635	-0.591314826834
O	-1.170279782765	-0.190693032788	-0.826094709834
C	-2.206539999821	-0.201957156649	0.142760758348
H	-1.774231491530	-0.448966407526	1.107517929239
C	-2.688544562760	1.246526959272	0.195659765191
H	-3.584654051667	1.290707324470	0.800206097942
H	-2.952308094834	1.571018912817	-0.809701530670
C	-3.296233270774	-1.200351429948	-0.239496230372
H	-2.811317125609	-2.127024300080	-0.528652109268
H	-3.815434793338	-0.836256763547	-1.123499787393
C	-4.294808473107	-1.489162510866	0.882410403931
H	-5.005116125546	-2.246668878747	0.567331528105
H	-4.863283194254	-0.609506046147	1.166813576532

H	-3.789874546513	-1.860371064772	1.770415154218
O	-1.765350071895	2.108092934678	0.783023810723
H	-1.030507116624	2.260360843750	0.208967457127

R-BuOH-O…O_2…*R*-BuOH-O

E(CASSCF(10,8)/6-311++G(d,p)) = -762.6922657 Eh
E(XMCQDPT2(10,8)/6-311++G(d,p)) = -764.9092768 Eh
0 1

H	2.325714925900	2.072199715000	0.936438061000
C	2.059920364500	1.028453268700	1.049361830500
H	2.157556765900	0.769677421500	2.102613390400
C	3.000832378500	0.162465948400	0.219861764400
H	2.863167994000	0.413903124200	-0.831857626300
C	4.469044331100	0.323939262500	0.609578864900
H	4.599166435800	-0.028293447400	1.630813670700
H	4.705303284100	1.385506209300	0.615353052600
C	5.428731386800	-0.415031126900	-0.320070534900
H	6.455083638300	-0.285212434000	0.008650629000
H	5.355786558500	-0.037608391100	-1.336827439300
H	5.212560653100	-1.477036431200	-0.340088844200
O	0.733251037300	0.898673117500	0.629870938900
H	0.447810578500	0.001637330000	0.707140927000
O	2.604860022200	-1.171770362200	0.295371085300
O	0.097147386800	-3.812005023100	-0.358077598300
O	0.026442072300	-3.015378701800	-1.272830635300
O	-1.724796855700	-0.838033945000	0.803504972700
C	-2.956572693200	-0.180385789100	0.731844015000
H	-3.283453398600	-0.134635919400	1.770541422600
C	-2.771248983700	1.246281844500	0.222264135800
H	-3.733978806200	1.739023820000	0.170873835400
H	-2.361695176300	1.214830237700	-0.787486274900
C	-3.934532294600	-1.030454890300	-0.082538071000
H	-3.873889619200	-2.043872974000	0.301297571900
H	-3.595434383100	-1.069935811500	-1.116004141200
C	-5.386187174900	-0.551524465900	-0.030957263300
H	-6.031439215500	-1.258924940800	-0.541992946700
H	-5.519363686000	0.413289305900	-0.508821776400
H	-5.735798791000	-0.467613795100	0.994848863800
O	-1.971309912400	2.009916935100	1.067310861500
H	-1.064306854500	1.759236892600	0.973009907300

R-BuOH-O_4-*S*-BuOH ((2*R*,2'S)-2,2'-tetraoxidanediylbis(butan-1-ol))

R-BuOH-O_2

-follow-this-link-

S-BuOH-O_2

-follow-this-link-

R-BuOH-O_2 + *S*-BuOH-O_2

E(CASSCF(10,8)/6-311++G(d,p)) = -762.6530270 Eh

E(XMCQDPT2(10,8)/6-311++G(d,p)) = -764.9502682 Eh
0 1

H	9.427644106100	0.009885439800	2.620972150100
C	9.377458044100	-0.698236560500	1.796894757200
H	9.689743774000	-0.162652764200	0.903449897200
C	7.919993558400	-1.108029088300	1.638483986600
H	7.575048606800	-1.693500225600	2.479648012900
C	6.992059403400	0.074951752100	1.417410986200
H	7.087942832700	0.755129453800	2.254181967700
O	7.769748903900	-1.936025147500	0.469148280000
O	7.702833877400	-3.241479983700	0.770086574700
O	-6.783392359000	-1.040390637300	-2.082190083900
O	-6.805541189300	0.238601644800	-1.680973910600
C	-8.013971841800	0.553404435400	-0.964292398300
H	-8.216506766200	-0.284255186400	-0.309448685000
C	-7.650683176100	1.775477660100	-0.137830572200
H	-7.340857680300	2.580728688000	-0.801689792900
H	-8.522271478800	2.109782549100	0.407330145700
C	-9.151600956500	0.769454901600	-1.954146388900
H	-9.171241948700	-0.086048695900	-2.620654727300
H	-8.930242740300	1.640327089200	-2.566898037000
C	-10.521484473700	0.921167651200	-1.290799288600
H	-10.747967815400	0.067266868900	-0.658176851900
H	-10.588433617000	1.816538894500	-0.681328186500
H	-11.296185882700	0.984927993900	-2.047520211200
O	-6.667110853700	1.500797035100	0.813520668100
H	-5.865588606500	1.256749986400	0.383943029000
H	7.289486462500	0.608602963200	0.516589024900
O	5.650730320200	-0.303289591500	1.357456775600
H	5.503054216400	-0.841830627700	0.599358230900
C	10.331531611000	-1.860599804000	2.066187862300
H	10.339601771100	-2.567146785200	1.244015686800
H	10.051385887900	-2.398530773700	2.967218732100
H	11.343568877400	-1.491411598300	2.198201147300

R-BuOH-O_2…*S*-BuOH-O_2

E(CASSCF(10,8)/6-311++G(d,p)) = -762.6612226 Eh
E(XMCQDPT2(10,8)/6-311++G(d,p)) = -764.9626106 Eh
0 1

H	4.525650943781	0.735005624252	1.658637886150
C	4.478197511357	0.031475929293	0.830451414165
H	4.792209230138	0.571951420435	-0.059514300316
C	3.021337271862	-0.379726662509	0.665935114247
H	2.673373071616	-0.963618084794	1.507097579180
C	2.098923750042	0.806298632399	0.442610612044
H	2.202465054539	1.493945144197	1.272069858132
O	2.872491031084	-1.204848620255	-0.503070929460
O	2.825236283656	-2.511785670932	-0.203238175704
O	-1.936824657289	-1.801349323486	-1.146744239057
O	-1.918543231988	-0.525987927090	-0.732864185109

C	-3.112923132482	-0.182419225212	-0.000837065061
H	-3.322234305631	-1.014784831499	0.658154490660
C	-2.731161847851	1.038463918240	0.821961303537
H	-2.369355927611	1.819374309757	0.154553836199
H	-3.619081920148	1.412483373321	1.314639806202
C	-4.255601070263	0.047522324423	-0.983011628208
H	-4.274022983604	-0.793221522464	-1.667967836792
H	-4.042861100855	0.933330215629	-1.577676800297
C	-5.624592171425	0.175563246738	-0.312725785628
H	-5.845616628423	-0.697020218466	0.295821278417
H	-5.693712577394	1.052460712252	0.322564075365
H	-6.401512902671	0.257439801664	-1.065700563188
O	-1.803324956632	0.750628357377	1.816969720611
H	-0.943012910322	0.639177636282	1.439969535290
H	2.385843260795	1.329768825315	-0.466434101784
O	0.752420261505	0.433030134340	0.394682522603
H	0.572755682486	-0.103509568521	-0.360247084082
C	5.432955434518	-1.131185691957	1.096001200395
H	5.445661747468	-1.832128064717	0.269244367477
H	5.150037251019	-1.675376470364	1.992302944318
H	6.443575243709	-0.760590337111	1.234514590422

[*R*-BuOH-OO···OO-*S*-BuOH]‡

E(CASSCF(10,8)/6-311++G(d,p)) = -762.6531091 Eh
E(XMCQDPT2(10,8)/6-311++G(d,p)) = -764.981310 Eh
$\tilde{\nu}^{\ddagger} = -212.28\ \mathrm{cm}^{-1}$
0 1

H	3.542530032586	0.165047248958	1.811166836190
C	3.297499690834	-0.475837333473	0.967037038039
H	4.152519926765	-0.453556530830	0.295108661399
C	2.097625427949	0.138659054055	0.256830507615
H	1.220388208417	0.118743248575	0.887133155247
C	2.366359261410	1.561865531108	-0.205153104864
H	2.700502337610	2.152680729950	0.637943207209
O	1.798105580246	-0.605267219858	-0.935241078750
O	0.793142116454	-1.535972500269	-0.711219183534
O	-0.617601280743	-0.982896280215	-1.583753243692
O	-1.054413240355	0.204566488812	-1.029887915583
C	-1.970171977684	-0.033234105410	0.047981247466
H	-1.492303025118	-0.728888932800	0.727898534715
C	-2.114521140181	1.322756695789	0.724770895504
H	-2.486494496080	2.044541162079	-0.000600121026
H	-2.850811721519	1.239645354807	1.512967829611
C	-3.283074487781	-0.600338656481	-0.482172290907
H	-3.045062184559	-1.413986426418	-1.157429387816
H	-3.786034773211	0.161905472724	-1.073370058759
C	-4.214103238263	-1.119795358104	0.614945834541
H	-3.722412163623	-1.876963863934	1.219842196346
H	-4.553550548813	-0.330939118994	1.278409786604
H	-5.095107970040	-1.573386426958	0.172815828524

O	-0.933318489590	1.765725572896	1.316050574332
H	-0.326201965642	2.062214065347	0.653896849368
H	3.158070689755	1.561097581093	-0.950831752889
O	1.218407364250	2.189402090570	-0.705538246104
H	0.871000211114	1.686977807189	-1.424485138199
C	3.067275136148	-1.901982182773	1.463383275995
H	2.862949770389	-2.579596302830	0.642060187526
H	2.229714550504	-1.947291749037	2.153904977018
H	3.947843102962	-2.265527729086	1.983683536690

R-BuOH-O_4-*S*-BuOH

E(CASSCF(10,8)/6-311++G(d,p)) = -762.6587190 Eh

E(XMCQDPT2(10,8)/6-311++G(d,p)) = -764.9853308 Eh

0 1

H	3.944887879753	0.152154417010	1.507681144208
C	3.525037130335	-0.485702430166	0.732619470054
H	4.230239267393	-0.481768132895	-0.095410233912
C	2.216241985511	0.138452435052	0.266346631280
H	1.479193828150	0.120834789742	1.058626780703
C	2.379973471383	1.571335113792	-0.220904230996
H	2.807352525320	2.174620392499	0.570148459307
O	1.684481231003	-0.593111247966	-0.839886412841
O	0.500970266283	-1.397882856279	-0.348400916259
O	-0.504422619555	-1.174227620042	-1.241759847926
O	-1.117190008901	0.148349315771	-0.904498807515
C	-2.086707219159	-0.029095496084	0.124702521013
H	-1.660499705283	-0.678256698138	0.881422797838
C	-2.252868454173	1.363019329716	0.723922269455
H	-2.529962325276	2.061131575287	-0.064633346706
H	-3.063879542903	1.338418567923	1.439798184310
C	-3.371327025190	-0.622052441044	-0.445554748249
H	-3.096776226325	-1.460967131841	-1.076424095977
H	-3.843485968387	0.112416848148	-1.094501215256
C	-4.358272354710	-1.100947421729	0.620343279917
H	-3.900356383490	-1.837066814068	1.275832176396
H	-4.725850452327	-0.287999642853	1.238351268672
H	-5.218944841001	-1.567701944570	0.152348463026
O	-1.119792082127	1.795301808042	1.408804681044
H	-0.433048196377	2.027076345804	0.801286023027
H	3.060910376721	1.596587896624	-1.069101573461
O	1.151888421420	2.151875182845	-0.559201703520
H	0.732966524073	1.625513705725	-1.221307819466
C	3.364956750731	-1.906894198142	1.268458170860
H	2.963392908625	-2.568254279050	0.508670433867
H	2.695792307239	-1.930353946840	2.124574452892
H	4.323849241204	-2.304438038911	1.585797184889

[*R*-BuOH-O···O_2···O-*S*-BuOH]‡

E(CASSCF(10,8)/6-311++G(d,p)) = -762.6565582 Eh

E(XMCQDPT2(10,8)/6-311++G(d,p)) = -764.9911462 Eh

$\tilde{\nu}^{\ddagger}$ = -310.26 cm^{-1}

0 1

H	4.150421177622	0.130629022457	1.371892167366
C	3.698438289911	-0.522203165837	0.627967129506
H	4.338075726240	-0.486543722242	-0.251100889212
C	2.330961472839	0.045042635236	0.264495822157
H	1.655581721905	-0.032334441326	1.108404626310
C	2.388010512992	1.501862283766	-0.180294897069
H	2.797662790665	2.110526013880	0.616273546662
O	1.767183551998	-0.694132516263	-0.808857609589
O	0.383860978779	-1.454656022362	-0.202528876680
O	-0.535876440828	-1.340597076090	-1.089123144910
O	-1.251192152614	0.136457938907	-0.902808404399
C	-2.216788075582	0.019228787411	0.129471703400
H	-1.805793303804	-0.605937179898	0.915512245297
C	-2.354127825508	1.433883090870	0.688755807914
H	-2.577283091694	2.121218293671	-0.126213389159
H	-3.190682744134	1.456989028537	1.374806239583
C	-3.514723636571	-0.571207315359	-0.414350829026
H	-3.254937774775	-1.434709312930	-1.018715846813
H	-3.976562785037	0.147696032698	-1.087862564798
C	-4.507204331462	-0.997939911287	0.668193259410
H	-4.060242984101	-1.719364005542	1.347324800573
H	-4.859201217953	-0.158874370525	1.259757741275
H	-5.377076591480	-1.465393228138	0.218076297776
O	-1.232640999266	1.840780324356	1.405715115818
H	-0.500962309309	1.993963228412	0.825976096817
H	3.041246326311	1.596055504623	-1.045655969724
O	1.116294566857	2.008838755483	-0.470366469092
H	0.705198194105	1.463150382971	-1.121913171282
C	3.635827356360	-1.950239290285	1.165012291362
H	3.195187433412	-2.621191447880	0.436006096587
H	3.041547655289	-2.001189390978	2.073794744204
H	4.631021337427	-2.315091097673	1.399094994343

R-BuOH-O…O_2…*S*-BuOH-O

E(CASSCF(10,8)/6-311++G(d,p)) = -762.6919787 Eh

E(XMCQDPT2(10,8)/6-311++G(d,p)) = -764.9555286 Eh

0 1

H	4.425519871700	0.401016086700	1.145947168300
C	4.053534775900	-0.363409981000	0.467694131300
H	4.632621576100	-0.274049165600	-0.449273705300
C	2.590138420500	-0.056034286800	0.157498234700
H	1.977637149400	-0.224842583700	1.043138246600
C	2.362863323300	1.373263117200	-0.322115961500
H	2.726569149700	2.073650295100	0.419336384500
O	2.072092050300	-0.949951103700	-0.778661263700
O	-0.271796103100	-3.219278872800	-0.078870028900
O	-0.151915298100	-3.736298933000	-1.171939694200
O	-1.643595112000	0.146206849200	-0.934335033200

C	-2.540347174200	0.103163738000	0.136207949600
H	-2.261752369300	-0.808601147700	0.664320051800
C	-2.303641386400	1.288405475100	1.069096462800
H	-2.508699334900	2.214305901500	0.531151232500
H	-2.990644380000	1.235136556800	1.903902941500
C	-3.969338916900	-0.019883386800	-0.395785598500
H	-3.969405771200	-0.822341271400	-1.126779149800
H	-4.226552565100	0.889586467900	-0.935566215100
C	-5.023811455200	-0.304247932100	0.674826948200
H	-4.768665423500	-1.189350532300	1.252126242800
H	-5.142045197100	0.522847854400	1.367009105600
H	-5.989468836600	-0.480221596500	0.211811635600
O	-1.018599772600	1.291699233500	1.604236140800
H	-0.385211941900	1.497729354100	0.931994323300
H	2.917321302600	1.541999271200	-1.243802613400
O	1.004736672100	1.650601643600	-0.509380789100
H	0.624088474000	1.030474230200	-1.111417361600
C	4.263894723000	-1.745619020400	1.081391073000
H	3.911335861300	-2.525851891400	0.416528801100
H	3.729864165600	-1.839541203800	2.023375388400
H	5.317117490400	-1.919481009700	1.278288228700

R-$PrNO_3$-O_4-*R*-$PrNO_3$ ((2*R*,2'*R*)-tetraoxidanediylbis(propane-2,1-diyl) dinitrate)

R-$PrNO_3$-O_2

E(CASSCF(5,4)/6-311++G(d,p)) = -545.7630878 Eh

0 2

H	2.747268176600	1.108061688400	0.874900867400
C	2.171551980400	1.440208602400	0.018964348400
H	1.754549404100	2.418285616000	0.234637744400
H	2.837797542800	1.532927489700	-0.830317441300
C	1.051979927900	0.466806512300	-0.308983539500
C	0.145462678800	0.228534180300	0.884761019100
H	-0.205535563900	1.165267200500	1.287452185000
H	0.659849749700	-0.330635826500	1.650048194700
O	-0.968738361800	-0.615485814700	0.577809670700
N	-2.001544788000	-0.060718061100	-0.054734887000
O	-1.938827608400	1.091559136900	-0.317809845100
O	-2.874954284900	-0.802681971800	-0.277615558400
H	0.476313500500	0.831008697600	-1.147350006900
O	1.566730887900	-0.773462078100	-0.807763137100
O	2.330380758200	-1.399987371900	0.100953385600

R-$PrNO_3$-O_2 + *R*-$PrNO_3$-O_2

E(CASSCF(10,8)/6-311++G(d,p)) = -1091.5278667 Eh

E(XMCQDPT2(10,8)/6-311++G(d,p)) = -1094.6721885 Eh

0 1

C	-6.290700803800	-3.544426092700	-0.597905671300
H	-5.744128521000	-4.472392050500	-0.641727596200
H	-7.287534897100	-3.723619941200	-0.222304119500
C	-6.350499800900	-2.885590668700	-1.962064082800

H	-5.403344407900	-2.434608412300	-2.217117527400
C	-6.791049091500	-3.866379899300	-3.034622440900
H	-6.041483148800	-4.640375647700	-3.161795230500
H	-6.911713589500	-3.350049029300	-3.978296126700
H	-7.736483088400	-4.331784424400	-2.772283284700
O	-7.309142875000	-1.830824812600	-1.828583181600
O	-7.173531033000	-0.930205479700	-2.812626994000
O	5.146632721000	6.391806795000	1.504823273000
O	5.702651864000	5.219404423000	1.167848572500
C	5.408520216200	4.214156415800	2.142217759000
H	4.360790936000	4.301685463300	2.393824146200
C	6.287622164800	4.396397131200	3.367606295700
H	6.132085334800	5.382376743000	3.786117769300
H	7.337493072300	4.290515459600	3.112682978400
H	6.034828263800	3.660319280700	4.123678276000
C	5.671704048900	2.891920715200	1.455887346400
H	6.627996494800	2.895476937300	0.953602386100
H	5.640117613800	2.081948521200	2.169997469200
O	-5.729055977400	-2.696719645800	0.407832742300
N	-4.400051656400	-2.605049767000	0.454497117900
O	-3.772370710000	-3.223335305000	-0.334944728800
O	-4.003711042000	-1.903884314000	1.299808370800
O	4.623130486100	2.724359570200	0.499037764100
N	4.682261107000	1.630435974000	-0.257401028900
O	5.578108974000	0.882210556400	-0.072934889500
O	3.811571574400	1.540279287600	-1.031547390700

R-PrNO_3-O_2…*R*-PrNO_3-O_2

E(CASSCF(10,8)/6-311++G(d,p)) = -1091.5362492 Eh
E(XMCQDPT2(10,8)/6-311++G(d,p)) = -1094.6872057 Eh
0 1

C	-2.908148049672	-1.536769076338	0.578215957871
H	-2.363668495219	-2.465210860546	0.539999161131
H	-3.902215240658	-1.706515488589	0.964161016841
C	-2.969032024066	-0.875453747421	-0.784571517808
H	-2.024187499626	-0.421782759573	-1.042944019562
C	-3.403626962519	-1.855067024366	-1.860083489746
H	-2.645429190641	-2.619352601708	-1.992536063301
H	-3.527003624484	-1.333529815608	-2.800273345809
H	-4.345207926719	-2.330004710795	-1.600018456895
O	-3.933730343937	0.173040330543	-0.639622928353
O	-3.824035021995	1.070839324553	-1.629910302084
O	1.762052108626	4.384236806538	0.322106580308
O	2.318071251904	3.211834434665	-0.014350234036
C	2.021623657445	2.204341696667	0.959160954474
H	0.974694318611	2.288596950795	1.210031307671
C	2.895466908731	2.382261117542	2.188371878512
H	2.740855981504	3.368198820976	2.607569716870
H	3.946622906852	2.272609023613	1.939414945474
H	2.631911404648	1.645606235187	2.939599584899

C	2.292439371042	0.885938655190	0.269340918512
H	3.244541810006	0.899093465121	-0.240312752401
H	2.262548204894	0.072341791258	0.977888949810
O	-2.333343280004	-0.676705806995	1.576244383155
N	-1.012551303345	-0.606807139940	1.631248785131
O	-0.379505931513	-1.330957260418	0.942323874235
O	-0.604279531795	0.187493326568	2.390256645143
O	1.237306730622	0.710967223513	-0.684982942619
N	1.287491789235	-0.389486755195	-1.423783656129
O	2.228370849398	-1.090186594962	-1.316234769344
O	0.355177708703	-0.535724428789	-2.122643748746

[*R*-PrNO_3-OO···OO-*R*-PrNO_3]‡

E(CASSCF(10,8)/6-311++G(d,p)) = -1091.5274150 Eh

E(XMCQDPT2(10,8)/6-311++G(d,p)) = -1094.7087535 Eh

$\tilde{\nu}^‡$ = -189.53 cm^{-1}

0 1

C	-2.479959607649	-1.279172693665	0.706968722224
H	-2.832785722191	-2.257767196560	0.426869988917
H	-3.096919763326	-0.878794209656	1.498242541517
C	-2.466602420653	-0.338686710543	-0.482585165951
H	-1.662309949424	-0.594754640087	-1.158514288914
C	-3.802952683476	-0.340442797680	-1.204694063245
H	-3.979574185279	-1.310454191913	-1.658221299087
H	-3.800636106297	0.405608476378	-1.988390091002
H	-4.616048805340	-0.119211935625	-0.519492460618
O	-2.178998477815	0.938241528939	0.082779254001
O	-1.895278633276	1.852937999193	-0.913961778183
O	-0.145096320502	1.652803598710	-1.322250788792
O	0.577352920844	2.454084170736	-0.468570504958
C	1.008632098050	1.731650208431	0.683998822534
H	0.227519145473	1.045043838702	0.967845081114
C	1.227282620876	2.772429864840	1.769334159136
H	0.303598448012	3.306601433523	1.952696367464
H	1.986701234883	3.489853753802	1.475034923818
H	1.540196133562	2.293332706113	2.690750724884
C	2.292052157020	0.978502942900	0.390331293298
H	3.037448528036	1.631950682183	-0.039718051852
H	2.676619793859	0.524306506969	1.292092178579
O	-1.195293437594	-1.384139912246	1.334623950691
N	-0.327461416703	-2.256874458454	0.819370456330
O	-0.670593144593	-2.907272868609	-0.100815716327
O	0.704873646047	-2.271168640501	1.372944280430
O	1.959969350946	-0.048611634520	-0.548494452722
N	2.958798210319	-0.830383525169	-0.949753365804
O	4.031582902995	-0.631629356750	-0.497323972036
O	2.641094055310	-1.644137806104	-1.727430315098

R-PrNO3-O_4-*R*-$PrNO_3$

E(CASSCF(10,8)/6-311++G(d,p)) = -1091.5333497 Eh
E(XMCQDPT2(10,8)/6-311++G(d,p)) = -1094.7168563 Eh
0 1

C	-2.452329214610	-1.295405912812	0.702977425896
H	-2.783960187665	-2.286655134615	0.441659101978
H	-3.118932799899	-0.866429140783	1.436825239061
C	-2.363452560209	-0.403946793246	-0.523358108888
H	-1.511014141446	-0.694453904286	-1.124150214443
C	-3.643272867871	-0.462580745507	-1.341178825141
H	-3.774994085447	-1.453586854286	-1.764088435756
H	-3.596097197462	0.249610986064	-2.155128164090
H	-4.506755756428	-0.223577217202	-0.727446694710
O	-2.134360453620	0.891295007572	0.004929694335
O	-1.672284359108	1.778831207866	-1.111490339496
O	-0.324452283509	1.595913585582	-1.241382108929
O	0.340760036689	2.518165036392	-0.261200787936
C	0.917166406439	1.752740241981	0.781750104633
H	0.202967063700	1.016251384596	1.118162327010
C	1.197394179725	2.756242472654	1.892403985082
H	0.275864023965	3.244413267984	2.182823500090
H	1.900991896715	3.515158615612	1.565323710357
H	1.609313271543	2.248938860917	2.757958523775
C	2.203499354598	1.073908473803	0.346483454087
H	2.859143939097	1.769039657127	-0.157563284412
H	2.708099619167	0.640358300364	1.197689006755
O	-1.209179039559	-1.367286879059	1.413293696357
N	-0.299569906707	-2.238824220162	0.975172274625
O	-0.588375587635	-2.942641768123	0.075769676485
O	0.710088907445	-2.198929092267	1.568548959592
O	1.835874720974	0.024700834768	-0.557907306388
N	2.830102341992	-0.695564989392	-1.068039835351
O	3.931610600798	-0.434163227451	-0.732435650071
O	2.479364651604	-1.527676919642	-1.812734493309

[*R*-$PrNO_3$-O···O_2···O-*R*-$PrNO_3$]‡

E(CASSCF(10,8)/6-311++G(d,p)) = -1091.5310300 Eh
E(XMCQDPT2(10,8)/6-311++G(d,p)) = -1094.7249951 Eh
$\tilde{\nu}^{\ddagger}$ = -318.49 cm^{-1}
0 1

C	-2.458788097101	-1.361761783641	0.727257205704
H	-2.727296873541	-2.370801585720	0.461147824376
H	-3.162124372060	-0.972654922464	1.448879425931
C	-2.397131453649	-0.465435180717	-0.500704080284
H	-1.517466007893	-0.720300699163	-1.080039113255
C	-3.653283696644	-0.604580812777	-1.346497026542
H	-3.723613318718	-1.604591556250	-1.763175654691
H	-3.627189736727	0.103171311491	-2.165808670853
H	-4.541937665976	-0.405734105386	-0.754749668693
O	-2.251193028043	0.836344350584	0.019827182420

O	-1.664270322527	1.785479817334	-1.224969049741
O	-0.402593140168	1.576564634188	-1.332395145496
O	0.386939623003	2.605227161691	-0.285843093689
C	0.931956130489	1.822531697990	0.751137443407
H	0.197431534283	1.100654271715	1.079588584181
C	1.225552716794	2.805562821193	1.879998520194
H	0.312700861644	3.310143941770	2.169759874173
H	1.949087266175	3.552645061334	1.569454424676
H	1.618584646232	2.276736490900	2.741601544569
C	2.205966070793	1.114461065746	0.325537487561
H	2.891061098392	1.800531816392	-0.150877778907
H	2.682180440304	0.642230748497	1.172484327935
O	-1.226303595815	-1.360740919552	1.459669666751
N	-0.257319604662	-2.170606103208	1.033309162864
O	-0.491485161612	-2.900704799124	0.139045987084
O	0.745161031975	-2.056237562951	1.630278590081
O	1.819422595805	0.101454273349	-0.612603512359
N	2.797936636663	-0.628723353534	-1.137929924442
O	3.903221546710	-0.407994360537	-0.786932595989
O	2.430896329078	-1.427669196437	-1.911263263420

R-PrNO$_3$-O…O$_2$…*R*-PrNO$_3$-O

E(CASSCF(10,8)/6-311++G(d,p)) = -1091.5692303 Eh

E(XMCQDPT2(10,8)/6-311++G(d,p)) = -1094.6362833 Eh

0 1

C	-2.848477045000	-1.641705706500	0.535872892800
H	-2.252713479000	-2.522572479400	0.713025095700
H	-3.867108026400	-1.812493990800	0.851262095400
C	-2.808803234300	-1.197224070300	-0.919985123600
H	-1.823668476800	-0.821153799300	-1.174068216300
C	-3.154751737300	-2.352609945900	-1.856120730700
H	-2.435597228400	-3.156316958600	-1.742998371700
H	-3.129861937400	-2.007997152400	-2.881841314900
H	-4.147260612000	-2.741611393000	-1.647009632000
O	-3.694845034400	-0.152865650900	-1.141739897400
O	-1.993660099600	3.254445805800	-1.459505854200
O	-1.782897067800	2.476254064800	-0.550496507400
O	2.199738323500	3.379756281100	-0.040716051900
C	1.982205470300	2.396457275400	0.914145255200
H	0.931416018400	2.429659247000	1.183160828300
C	2.830281683500	2.736035418600	2.138413853800
H	2.572783888200	3.724060412200	2.498569379100
H	3.888359127100	2.722464824600	1.895587814300
H	2.645641700400	2.018218763400	2.929584621100
C	2.324622512800	1.017714422500	0.377473256700
H	3.313298125500	1.002679909000	-0.057280690400
H	2.238335877600	0.259174588600	1.141490574700
O	-2.429190121600	-0.593454700600	1.431302018400
N	-1.129828810400	-0.417303512600	1.597445982400
O	-0.384217231700	-1.167426493800	1.065346589300

O	-0.850194010200	0.489969902700	2.285665211400
O	1.351098693100	0.752426991500	-0.644295070600
N	1.453042104300	-0.406663756700	-1.275210237000
O	2.377161051200	-1.096128802600	-1.033163539400
O	0.578345415500	-0.616199435500	-2.032650961200

R-$PrNO_3$-O_4-*S*-$PrNO_3$ ((2*R*,2'*S*)-tetraoxidanediylbis(propane-2,1-diyl) dinitrate)

R-$PrNO_3$-O_2

-follow-this-link-

S-$PrNO_3$-O_2

E(CASSCF(5,4)/6-311++G(d,p)) = -545.7630878 Eh

0 2

H	-1.754750063300	2.418278016500	0.234763387100
C	-2.171641684300	1.440173840000	0.019003737200
H	-2.747341811900	1.107893869200	0.874903918400
H	-2.837882198100	1.532880986500	-0.830283591300
C	-1.051958260600	0.466911139200	-0.308980991400
H	-0.476296972500	0.831225518500	-1.147302813800
C	-0.145449977400	0.228672269900	0.884780135000
H	-0.659870684800	-0.330400513400	1.650110062100
H	0.205600442900	1.165422905800	1.287393335900
O	-1.566536117000	-0.773387527600	-0.807850405600
O	-2.330116003600	-1.400083835600	0.100809983900
O	0.968691201000	-0.615451477100	0.577912925700
N	2.001519034000	-0.060871617500	-0.054751813900
O	1.938909552500	1.091378628300	-0.317978428700
O	2.874839543300	-0.802955202700	-0.277576440500

R-$PrNO_3$-O_2 + *S*-$PrNO_3$-O_2

E(CASSCF(10,8)/6-311++G(d,p)) = -1091.5277077 Eh

E(XMCQDPT2(10,8)/6-311++G(d,p)) = -1094.6720507 Eh

0 1

C	-6.198256437200	3.561101392700	3.195289370900
H	-7.276285925400	3.569466621200	3.126348410000
H	-5.898990219700	3.169061002900	4.156354486900
C	-5.641736108000	4.950270807200	2.973137336400
H	-5.701704933100	5.213392358500	1.926367761700
C	-6.340857808100	5.982056583200	3.841543121600
H	-7.380257345400	6.077848181800	3.545142839100
H	-5.862249360500	6.945686062300	3.722994394600
H	-6.299556057700	5.701860622800	4.889485128200
O	-4.256104762100	4.868698042400	3.317191864300
O	-3.571945986300	5.898328359400	2.798371455000
O	6.661860020000	-1.644951029000	-5.126266189000
O	7.015564541000	-2.583234284000	-4.236854721200
C	5.994885106700	-3.578543906700	-4.105852413700
H	5.047786395200	-3.066278517100	-4.024825981000
C	6.325124329500	-4.318255997500	-2.824431995800
H	7.374302721400	-4.574301373500	-2.797923528600
H	5.728419114000	-5.211026979300	-2.730743982400

C	6.006857602400	-4.512103737800	-5.303640760800
H	6.953042423700	-5.040020624400	-5.375506323000
H	5.205519649000	-5.238214865200	-5.216762287700
O	-5.661096993800	2.746497724400	2.150663626500
N	-5.991399709700	1.457169874500	2.175991287600
O	-6.705388212600	1.089221168700	3.042616224100
O	-5.523761664800	0.829757935300	1.307962326900
H	5.857879693300	-3.946226150700	-6.214172565000
O	6.173611056100	-3.507392967900	-1.656344540600
N	4.937668839600	-3.347853917900	-1.182506353000
O	4.879342002700	-2.676269005300	-0.229442669900
O	4.050953549600	-3.885806460300	-1.751848173500

R-$PrNO_3$-O_2…*S*-$PrNO_3$-O_2

E(CASSCF(10,8)/6-311++G(d,p)) = -1091.5362144 Eh
E(XMCQDPT2(10,8)/6-311++G(d,p)) = -1094.6894280 Eh
0 1

C	-2.350834185299	0.706189851156	0.151464913977
H	-3.424923370063	0.727979172755	0.038878293808
H	-2.088361835288	0.309525404442	1.120073311153
C	-1.770298926203	2.087285514164	-0.057893854168
H	-1.863783295984	2.379765138374	-1.095097222300
C	-2.415222321958	3.108265682168	0.863243791512
H	-3.468260092210	3.214008875788	0.623180890149
H	-1.938449454386	4.071294647901	0.736057733294
H	-2.319669747039	2.807709715359	1.901584878717
O	-0.372547505011	1.964767702323	0.225928213339
O	0.301305432820	3.040048065180	-0.210779153278
O	2.768071557524	1.223100045599	-2.117115580343
O	3.121776079100	0.284816790610	-1.227259514880
C	2.101706704254	-0.712599003633	-1.098996697591
H	1.153479265552	-0.203504048748	-1.023353492140
C	2.430544882041	-1.447974203899	0.184944940384
H	3.480151502095	-1.700662209190	0.217138049738
H	1.834853229916	-2.340330080514	0.282980110520
C	2.123945836834	-1.651687931618	-2.292301311026
H	3.072351001515	-2.177670081732	-2.355284621580
H	1.319304521538	-2.373096775884	-2.210852470120
O	-1.779420297875	-0.121226683754	-0.868432826885
N	-2.063057963410	-1.417292038680	-0.801586583058
O	-2.878409580476	-1.766338204943	-0.026983225932
O	-1.453986117303	-2.075967976368	-1.558202618843
H	1.982119456597	-1.086901548750	-3.204674657120
O	2.276122290848	-0.629771234609	1.353609655029
N	1.054961668437	-0.503124219462	1.855247969382
O	0.993259097956	0.176457374781	2.802450591994
O	0.167050225116	-1.081336114310	1.322031955743

[*R*-$PrNO_3$-OO···OO-*S*-$PrNO_3$]‡

E(CASSCF(10,8)/6-311++G(d,p)) = -1091.5297631 Eh

E(XMCQDPT2(10,8)/6-311++G(d,p)) = -1094.7139069 Eh
$\tilde{\nu}^{\ddagger}$ = -211.31 cm^{-1}
0 1

C	-2.295556386279	0.646819772860	0.032004042314
H	-3.326914774998	0.826287894826	-0.234872595012
H	-2.232080913684	0.337149466404	1.064533097104
C	-1.448969096974	1.875087690816	-0.229416975593
H	-1.298391720032	1.996225933600	-1.294091968719
C	-2.061458341338	3.123008461210	0.383317570790
H	-3.003244575510	3.358469921278	-0.102314332432
H	-1.391115915740	3.962782843047	0.251277505189
H	-2.237826923087	2.986216723327	1.445491832683
O	-0.185470930451	1.586852607957	0.362319637259
O	0.781726990647	2.446021195155	-0.133511649691
O	1.507637011196	1.645866730202	-1.531726975971
O	2.417761282119	0.740275338623	-1.035569314031
C	1.842956665489	-0.567686788129	-0.956424877270
H	0.780445997522	-0.461817741447	-0.840827577915
C	2.450681810532	-1.220340437417	0.270256978571
H	3.528224552552	-1.146960841095	0.247692487921
H	2.155979562182	-2.255786594386	0.338590344509
C	2.167238073960	-1.354208515079	-2.215664424475
H	3.239902849674	-1.469760703449	-2.340506188533
H	1.706733567614	-2.335779055205	-2.175690880608
O	-1.758081677333	-0.380011690499	-0.809982692650
N	-2.244124502563	-1.606443900358	-0.641591647481
O	-3.155613556590	-1.743325619275	0.092077749659
O	-1.693944125681	-2.423590264557	-1.276970151485
H	1.776947528687	-0.832578904301	-3.081344143168
O	2.131009178335	-0.543788236395	1.492695740096
N	0.925144324027	-0.742367006793	2.008556610503
O	0.733488964155	-0.173166251551	3.010338655065
O	0.180693146077	-1.461720408882	1.431355612691

R-PrNO$_3$-O$_4$-*S*-PrNO$_3$

E(CASSCF(10,8)/6-311++G(d,p)) = -1091.5341106 Eh
E(XMCQDPT2(10,8)/6-311++G(d,p)) = -1094.7173119 Eh
0 1

C	-2.335193405166	0.631505732742	-0.006836082521
H	-3.370984868177	0.810258210690	-0.257103366645
H	-2.251220715073	0.363623568313	1.036011214024
C	-1.482929892395	1.840891494336	-0.339877746605
H	-1.370213589245	1.907248539144	-1.415283664491
C	-2.073833361231	3.121900907145	0.225401270329
H	-3.024619137084	3.344726969254	-0.249042570057
H	-1.400252626408	3.949852696111	0.041444439021
H	-2.225963541234	3.036491968931	1.296575034904
O	-0.217471663767	1.560341688012	0.232395202825
O	0.803527126196	2.419172483222	-0.438285502769
O	1.348913820684	1.667875620830	-1.449970146021

O	2.380457766410	0.798770175847	-0.818916964339
C	1.908252574233	-0.542084553259	-0.855326706977
H	0.834119829397	-0.530555330434	-0.801351010068
C	2.479738841621	-1.209496623592	0.383809922805
H	3.552931583350	-1.092532367115	0.424167455729
H	2.225029651183	-2.257808356548	0.404785014295
C	2.367106462437	-1.249109616456	-2.120764174606
H	3.451061929991	-1.281302313793	-2.179911795888
H	1.980229655419	-2.262791706916	-2.152013427861
O	-1.824995038193	-0.431898096302	-0.819118801326
N	-2.327382469565	-1.644035203081	-0.602043224614
O	-3.223001152448	-1.742365858225	0.157680730434
O	-1.807903077932	-2.489267267281	-1.225491745786
H	1.995272494525	-0.719764564133	-2.990169262608
O	2.068226122793	-0.584282547297	1.606055716354
N	0.832202359286	-0.810527198735	2.029351714999
O	0.552186013002	-0.245185406773	3.013179011250
O	0.150486368880	-1.547921423095	1.400650934472

[*R*-PrNO$_3$-O···O$_2$···O-*S*-PrNO$_3$]‡

E(CASSCF(10,8)/6-311++G(d,p)) = -1091.5306378 Eh

E(XMCQDPT2(10,8)/6-311++G(d,p)) = -1094.7246206 Eh

$\tilde{\nu}^‡$ = -337.24 cm^{-1}

0 1

C	-2.390881074191	0.600700692513	0.017813073273
H	-3.431174197523	0.747980686164	-0.235338411065
H	-2.303118124799	0.317219938987	1.056134282516
C	-1.574403064032	1.844597506605	-0.290912928077
H	-1.465628135158	1.921248557168	-1.366971078988
C	-2.217317691625	3.100158607548	0.276678322021
H	-3.174504062947	3.292302294460	-0.198751608418
H	-1.572332977880	3.952818080317	0.100629922301
H	-2.368690266553	3.005054234646	1.347205682563
O	-0.310588568474	1.604501609241	0.283131798490
O	0.818095153236	2.508308129669	-0.603262841698
O	1.267995367837	1.758685554406	-1.534450449126
O	2.491129595429	0.813935591930	-0.884364812042
C	1.995605583557	-0.510553337961	-0.893412465725
H	0.920110648638	-0.479110032709	-0.836919649599
C	2.543931843270	-1.174947091123	0.360776057122
H	3.618632577119	-1.076169834934	0.410857776785
H	2.271416713503	-2.218493671695	0.390929740325
C	2.434887604345	-1.258495494147	-2.143416064276
H	3.517834723805	-1.320262869632	-2.201478645446
H	2.022298511476	-2.262470137861	-2.154821287060
O	-1.840356150320	-0.429697120116	-0.810633848695
N	-2.285256113532	-1.665444398617	-0.604965556811
O	-3.168675805535	-1.814337708441	0.160983322682
O	-1.734130517602	-2.478801717556	-1.243824931078
H	2.078834081577	-0.737158388369	-3.024281850036

O	2.129737773267	-0.529625219343	1.571552732856
N	0.893043812701	-0.750485566358	1.996480845637
O	0.614336226477	-0.177346217825	2.976182993562
O	0.210032876194	-1.491628374027	1.373780370482

R-PrNO$_3$-O…O$_2$…*S*-PrNO$_3$-O

E(CASSCF(10,8)/6-311++G(d,p)) = -1091.5686081 Eh
E(XMCQDPT2(10,8)/6-311++G(d,p)) = -1094.6773246 Eh
0 1

C	-2.404965109400	0.532995921900	-0.005433122100
H	-3.456809713000	0.524268230600	-0.255669388100
H	-2.246009133800	0.067872477100	0.955045045400
C	-1.834519434700	1.940614337300	-0.042636996700
H	-1.927596439700	2.343552234700	-1.047971102600
C	-2.552353616300	2.856970008400	0.945551034100
H	-3.612829528300	2.895460663800	0.720780731700
H	-2.145978611300	3.858227985100	0.878613285100
H	-2.426043469000	2.500233423600	1.962413975900
O	-0.480397813400	1.948058553100	0.262357033100
O	1.487775552800	3.285627368300	-2.883264989500
O	0.761940789800	2.313009343000	-2.947097614500
O	3.042746908900	0.563081227400	-0.792726083100
C	2.236432821500	-0.566167386500	-0.750097749300
H	1.210931993800	-0.219609677300	-0.809051393300
C	2.455439069200	-1.343174216500	0.540142055200
H	3.509893396200	-1.508052662600	0.705743266300
H	1.927391695600	-2.283116512600	0.546389638800
C	2.545177944600	-1.431536205500	-1.969748409000
H	3.572810348900	-1.783259794300	-1.947629115300
H	1.880903855700	-2.287113575000	-1.998040536100
O	-1.674974692400	-0.193626753400	-1.004094036700
N	-1.883780285000	-1.502762749000	-1.058405441400
O	-2.763963458500	-1.951679271300	-0.417372609600
O	-1.147617417400	-2.072550505600	-1.774532618900
H	2.399758440600	-0.852613311100	-2.872812068000
O	2.084126385200	-0.577427155800	1.700973307400
N	0.801839963900	-0.549035931000	2.028784459000
O	0.555307524000	0.124365248600	2.952738912000
O	0.042652603900	-1.195951298700	1.388149495600

References

1. Vaucher, A. C.; Reiher, M., Steering Orbital Optimization out of Local Minima and Saddle Points Toward Lower Energy. *Journal of Chemical Theory and Computation* **2017**, *13*, 1219-1228.
2. Werner, H.-J.; Knowles, P. J.; Knizia, G.; Manby, F. R.; Schütz, M., Molpro: A General-purpose Quantum Chemistry Program Package. *WIREs Computational Molecular Science* **2012**, *2*, 242-253.
3. Neese, F., The ORCA Program System. *WIREs Computational Molecular Science* **2012**, *2*, 73-78.
4. Neese, F., Software Update: The ORCA Program System, Version 4.0. *WIREs Computational Molecular Science* **2018**, *8*, e1327.
5. Granovsky, A. A. *Firefly*, 8.2.0.
6. Schmidt, M. W.; Baldridge, K. K.; Boatz, J. A.; Elbert, S. T.; Gordon, M. S.; Jensen, J. H.; Koseki, S.; Matsunaga, N.; Nguyen, K. A.; Su, S., et al., General Atomic and Molecular Electronic Structure System. *Journal of Computational Chemistry* **1993**, *14*, 1347-1363.
7. Andrienko, G. A. *ChemCraft - Graphical Software for Visualization of Quantum Chemistry Computations*, 1.8 (Build 562b); 2019.
8. Atkinson, R.; Baulch, D. L.; Cox, R. A.; Crowley, J. N.; Hampson, R. F.; Hynes, R. G.; Jenkin, M. E.; Rossi, M. J.; Troe, J.; Subcommittee, I., Evaluated Kinetic and Photochemical Data for Atmospheric Chemistry: Volume II - Gas Phase Reactions of Organic Species. *Atmos. Chem. Phys.* **2006**, *6*, 3625-4055.
9. Glowacki, D. R.; Liang, C.-H.; Morley, C.; Pilling, M. J.; Robertson, S. H., MESMER: An Open-Source Master Equation Solver for Multi-Energy Well Reactions. *The Journal of Physical Chemistry A* **2012**, *116*, 9545-9560.
10. Halkiadakis, E. A.; Bowrey, R. G., The Estimation of Molecular Parameters for the Stockmayer (12-6-3) Potential Using Critical Properties. *Chemical Engineering Science* **1975**, *30*, 53-60.
11. Joback, K. G.; Reid, R. C., Estimation of Pure-component Properties from Group-contributions. *Chemical Engineering Communications* **1987**, *57*, 233-243.
12. Georgievskii, Y.; Klippenstein, S. J., Long-range Transition State Theory. *The Journal of Chemical Physics* **2005**, *122*, 194103.
13. Epifanovsky, E.; Gilbert, A. T. B.; Feng, X.; Lee, J.; Mao, Y.; Mardirossian, N.; Pokhilko, P.; White, A. F.; Coons, M. P.; Dempwolff, A. L., et al., Software for the Frontiers of Quantum Chemistry: An Overview of Developments in the Q-Chem 5 Package. *The Journal of Chemical Physics* **2021**, *155*, 084801.
